# How (5′S) and (5′R) 5′,8-Cyclo-2′-Deoxypurines Affect Base Excision Repair of Clustered DNA Damage in Nuclear Extracts of xrs5 Cells? A Biochemical Study

**DOI:** 10.3390/cells10040725

**Published:** 2021-03-24

**Authors:** Karolina Boguszewska, Michał Szewczuk, Julia Kaźmierczak-Barańska, Bolesław T. Karwowski

**Affiliations:** DNA Damage Laboratory of Food Science Department, Faculty of Pharmacy, Medical University of Lodz, ul. Muszynskiego 1, 90-151 Lodz, Poland; karolina.boguszewska@umed.lodz.pl (K.B.); michal.szewczuk@umed.lodz.pl (M.S.); julia.kazmierczak-baranska@umed.lodz.pl (J.K.-B.)

**Keywords:** BER, 5′,8-cyclo-2′-deoxyadenosine (cdA), 5′,8-cyclo-2′-deoxyguanosine (cdG), DNA repair, clustered DNA damage

## Abstract

The clustered DNA lesions (CDLs) are a characteristic feature of ionizing radiation’s impact on the human genetic material. CDLs impair the efficiency of cellular repair machinery, especially base excision repair (BER). When CDLs contain a lesion repaired by BER (e.g., apurinic/apyrimidinic (AP) sites) and a bulkier 5′,8-cyclo-2′-deoxypurine (cdPu), which is not a substrate for BER, the repair efficiency of the first one may be affected. The cdPus’ influence on the efficiency of nuclear BER in xrs5 cells have been investigated using synthetic oligonucleotides with bi-stranded CDL (containing (5′S) 5′,8-cyclo-2′-deoxyadenosine (ScdA), (5′R) 5′,8-cyclo-2′-deoxyadenosine (RcdA), (5′S) 5′,8-cyclo-2′-deoxyguanosine (ScdG) or (5′R) 5′,8-cyclo-2′-deoxyguanosine (RcdG) in one strand and an AP site in the other strand at different interlesion distances). Here, for the first time, the impact of ScdG and RcdG was experimentally tested in the context of nuclear BER. This study shows that the presence of RcdA inhibits BER more than ScdA; however, ScdG decreases repair level more than RcdG. Moreover, AP sites located ≤10 base pairs to the cdPu on its 5′-end side were repaired less efficiently than AP sites located ≤10 base pairs on the 3′-end side of cdPu. The strand with an AP site placed opposite cdPu or one base in the 5′-end direction was not reconstituted for cdA nor cdG. CdPus affect the repair of the other lesion within the CDL. It may translate to a prolonged lifetime of unrepaired lesions leading to mutations and impaired cellular processes. Therefore, future research should focus on exploring this subject in more detail.

## 1. Introduction

**Figure 1:**
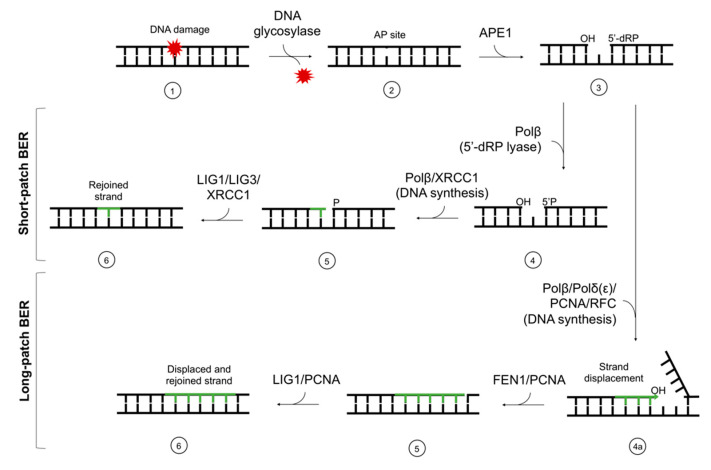
The scheme of short-patch and long-patch base excision repair (BER). The main stages of the repair are (**1**) damage recognition by DNA glycosylases, (**2**) excision of the damaged base and AP site formation, (**3**) strand incision, (**4**) end processing, (**4a**) end processing and strand displacement, (**5**) DNA synthesis (gap-filling), and (**6**) ligation. AP site—apurinic/apyrimidinic site; APE1—AP endonuclease 1; Polβ,δ,ε—polymerases β, δ, ε; XRCC1—X-ray repair cross-complementing protein 1; FEN1—flap structure-specific endonuclease 1; PCNA—proliferating cell nuclear antigen; RFC—replication factor C; LIG1—ligase I; LIG3—ligase III.

Every living cell is constantly exposed to DNA damaging factors, e.g., reactive oxygen species (ROS), endogenous metabolites, replication errors, chemotherapeutics, ionizing radiation, etc. Approximately 10^2^–10^5^ lesions form daily in every human cell [1]. When undetected and/or unrepaired by the cellular machinery, those lesions may lead to mutagenesis, cell death, or carcinogenesis. To prevent mutations and their consequences, cells have specialized repair mechanisms such as direct enzymatic repair (e.g., photolyases acting upon cyclobutane pyrimidine dimers [2]) or systems excising the damage followed by insertion of new nucleotide or fragment of nucleotide chain. To manage various lesion types effectively, a number of repair systems have developed, e.g., mismatch repair (MMR), base excision repair (BER), nucleotide excision repair (NER), non-homologous end-joining (NHEJ), or homologous recombination (HR) [3]. While NER removes bulky DNA damage (including 5′,8-cyclo-2′-deoxypurines (cdPus)); the most common repair mechanism is BER. It corrects a single nucleotide lesion (short-patch BER, (SP–BER)) or a fragment of 2–12 nucleotides (long-patch BER, (LP–BER)) [4]. The ability to excise a single base (not only a nucleotide or longer fragment of nucleotides) is its distinctive characteristic [4]. Moreover, BER is the most evolutionary conserved repair pathway in living organisms [4,5]. BER includes the following steps: damage recognition, damaged base excision, filling a gap with the correct nucleotide (or fragment of nucleotide chain), and strand ligation (Figure 1).

Approximately 70 types of DNA lesions are described. The most common ones have the chemical structure of nucleobases modified (alkylation, deamination, oxidation), which may lead to helix distortion [4]. One of the most frequently occurring lesions is 8-oxo-7,8-dihydroguanine (8-oxo-dG; 1 in every 10^6^ deoxyguanosine) or apurinic/apyrimidinic sites (AP sites), which are chemically unstable and thus highly mutagenic (1.6–3.3 in every 10^7^ nucleotides in mammalian tissues) [6,7]. Isolated DNA lesions are detected by glycosylases, such as evolutionary conserved uracil-DNA glycosylase (UDG). UDG recognizes and excises uracil from single-stranded DNA (ssDNA) and double-stranded DNA (dsDNA) through hydrolyzing β-N-glycosidic bond between base and sugar residue in DNA leading to AP site formation [8]. In this study, synthetic oligonucleotides with AP sites obtained from 2′-deoxyuridine (dU) excision by UDG enzyme were used as a substrate for repair assays. AP sites are mainly incised by AP endonuclease 1 (APE1), which is the main human endonuclease.

**Figure 2:**
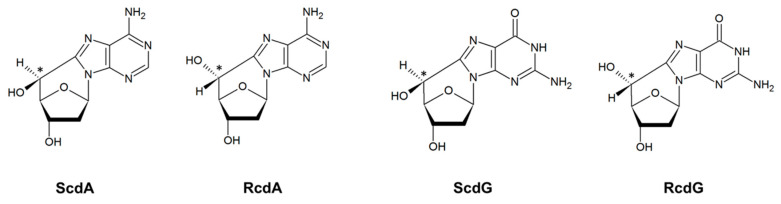
The structures of 5′,8-cyclo-2′-deoxypurines: (5′S)-5′,8-cyclo-2′-deoxyadenosine (ScdA), (5′R)-5′,8-cyclo-2′-deoxyadenosine (RcdA), (5′S)-5′,8-cyclo-2′-deoxyguanosine (ScdG), and (5′R)-5′,8-cyclo-2′-deoxyguanosine (RcdG).

Other types of lesions include single- and double-stranded breaks (SSB and DSB), tandem lesions, or clustered DNA lesions (CDL) [9]. CDL are defined as the presence of two or more lesions within 1–2 turns of the DNA helix [10]. They are characteristic for ionizing radiation impact on DNA and are more difficult to repair than individual lesions [10,11]. CDL may also be a result of chemotherapeutics’ action [9]. Tandem lesions originate from the covalent bonding between adjacent nucleosides (e.g., pyrimidine dimers) or the formation of more than one modification within one nucleotide (e.g., cdPus) resulting from a single radiation track [12]. CdPus arise from the action of oxidative radicals or radiation—the C5′ has an additional bond to the nucleobase, which increases the rigidity and subsequently distorts the DNA helix (Figure 2) [13].

CdPus are repaired by NER, which is inhibited or not active in some scenarios, e.g., in mitochondria or diseases with defective NER such as *Xeroderma pigmentosum*, trichothiodystrophy, or Cockayne syndrome [14,15,16,17,18,19]. Interestingly, 5′R diastereomer is repaired more efficiently than 5′S for both, 5′,8-cyclo-2′-deoxyadenosine (cdA) and 5′,8-cyclo-2′-deoxyguanosine (cdG), indicating the biological importance of stereochemistry of cdPus [15].

CdA and cdG are not suitable substrates for BER since there are no known glycosylases detecting these structures [20]. CdPus are found in the DNA of various cells and organisms [13]. The level of RcdA and ScdA in humans ranges from 0.01 to 0.1 lesions per 10^6^ DNA nucleosides, while RcdG and ScdG amount to 2 and 10 lesions per 10^6^ DNA nucleosides, respectively [21,22]. Their occurrence influences the repair of other lesions in a cluster, especially when NER is inactive. That being the case, lesions are usually repaired by BER in a sequence and one at a time to avoid the risk of errors, strand breaks, and further mutations [9]. CdPus affect the geometry of the DNA helix in the 5′ direction from the lesion and inhibit the activity of the BER system [23,24]. It impacts the ability of particular repair enzymes to form complexes with DNA leading to a decline in repair capacity [10,25,26]. Depending on the type and location of the lesion within the cluster, only the first one may be corrected. In nucleosomal-bound DNA, the cleavage of two AP sites in +1 position is reduced due to major structural changes needed in APE1 to incise damaged DNA [27]. However, AP sites in the −1 position do not require major changes in enzyme’s conformation, hence allowing the action of APE1. Studies show that in clusters containing ScdA and AP sites, the repair is inhibited for AP sites located closer than eight nucleobases to ScdA [12]. What is more, the activity of enzymes involved in the first two steps of the BER pathway (UDG and APE1) is reduced for CDL containing cdA [25].

This study shows how the presence of complex lesions (RcdA, ScdA, RcdG, or ScdG) and its relative distance to the single lesion (AP site) on the opposing strand influence the repairability of the latter. The repair of bi-stranded clustered DNA damage was tested in nuclear extracts (NE) of xrs5 cells (X-ray sensitive Chinese hamster ovarian mutant cell line), which is an established model to study BER [12,28,29,30,31]. Knowing the nature, interdependence, and implications of this type of damage is particularly important for its diagnostic potential. CdPus are considered biomarkers of oxidative DNA damage in the case of atherosclerosis, prediabetes, or inflammatory bowel disease [32,33,34]. Additionally, understanding the impact of radiation on genetic material is crucial for its new applications in medicine, pharmacy, and food science [35].

## 2. Materials and Methods

### 2.1. Substrate Oligonucleotides

The substrate oligonucleotides used in the experiments were synthesized and purified in the Bioorganic Chemistry Department of the Polish Academy of Science (Lodz, Poland) on a Geneworld synthesizer (K&A Laborgeraete GbR, Schaafheim, Germany) from nucleotide phosphoramidites bought from the ChemGenes Corporation (Wilmington, MA, USA). The phosphoramidite derivatives of cdPus were synthesized as previously described by Romieu et al. [36]. The crude oligonucleotides were purified on HPLC (C-18 column) using Varian analytics with UV detection (λ = 260 nm), Phenomenex, Warsaw, Poland (Synergi 4 μm Fusion-RP 80Å, 250 × 4.6 mm). The oligonucleotides’ concentration was determined by Varian Cary 1.3E spectrophotometer (Varian, Brunn am Gebirge, Austria) from a measurement of maximum absorbance (λ = 260 nm). The sequences of double-stranded oligonucleotides are presented in Table 1. Each dU residue has a number assigned—it describes the distance (number of base pairs) between cdA/cdG and dU located in the 5′ (positive number) or 3′ (negative number) direction on the opposing strand. Previous studies show that the melting temperatures of oligonucleotides containing cdPu are above 70 °C; therefore, they are stable in experimental conditions [25].

### 2.2. Mass Spectrometry of Oligonucleotides

Oligonucleotides were analyzed on a Waters Synapt G2-Si HDMS quadrupole time of flight hybrid mass spectrometer (Waters, Manchester, UK) in the negative-ion mode. Samples were dissolved in 10 mM ammonium acetate with 50% acetonitrile to a concentration of 0.1 OD/mL. Analysis parameters were as follows: flow rate, 10 μL/min; capillary voltage, 2.6 kV; cone voltage, 40 V; the source temperature, 120 °C; the desolvation temperature, 400 °C; cone gas, 30 L/h; and desolvation gas, 600 L/h. The data were obtained in full-scan negative ion mode (mass range of 50–2000 m/z) and processed with Waters MassLynx 4.1 software (deconvolution with MaxEnt1 function, Waters Corporation, Milford, MA, USA). Calculated and found masses are presented in Table 2 and mass spectra are presented in Figure S14.

### 2.3. Preparation of 5′-^32^P-End-Labeled Oligonucleotides

The 40-mer single-stranded oligonucleotides (230 pmol) were 5′-^32^P-end-labeled using 5U of T4 polynucleotide kinase (New England BioLabs, Ipswich, MA, USA) with 2 μCi (0.2 μL) [γ-^32^P]ATP (3000 Ci/mmol, 10 mCi/mL, Hartmann Analytic GmbH, Braunschweig, Germany) in 20 μL of buffer (pH 7.6 at 25 °C, 70 mM Tris-HCl, 10 mM MgCl_2_, 5 mM DTT) for 30 min at 37 °C. The proteins in the sample were denaturated (5 min, 95 °C). The purity of the single-stranded ^32^P-oligonucleotides was examined on 15% native polyacrylamide gel (Figure S1).

### 2.4. Hybridization of Oligonucleotides

The 5′-^32^P-end-labeled oligonucleotides were hybridized (10 min, 90 °C, followed by slow cooling over 3–4 h) with a two-fold excess of the purified complementary strand (non-radiolabeled) in pure H_2_O. Obtained duplexes were precipitated with 250 μL of cold ethanol (placed on dry ice, 30 min) and centrifuged (13,000× *g* rpm, 4 °C, 30 min). Ethanol was removed, and samples were dried under reduced pressure at room temperature. Efficient annealing and the purity of single-stranded oligonucleotides were verified on the 15% native polyacrylamide gel (Figure S1).

### 2.5. Preparation of AP Sites

The dry 5′-^32^P-end-labeled double-stranded oligonucleotides containing uracil were treated with 5U of UDG (New England BioLabs Ipswich, MA, USA) in 20 μL of the reaction buffer (pH 8.0 at 25 °C, 20 mM Tris-HCl, 1 mM EDTA, 1 mM DTT) at 37 °C for 30 min. Obtained AP sites were precipitated with 250 μL of cold ethanol (placed on dry ice, 30 min) and centrifuged (13,000× *g* rpm, 4 °C, 30 min). Formation of AP sites was confirmed by treatment with 5U APE1 (New England BioLabs Ipswich, MA, USA) in 10 μL of the reaction buffer (pH 7.9 at 25 °C, 50 mM potassium acetate, Tris-acetate, 10 mM magnesium acetate, 1 mM DTT) at 37 °C for 30 min to produce SSB. The purity of the double-stranded ^32^P-oligonucleotides containing AP sites and SSB were examined on 15% denaturing polyacrylamide gel (Figure S2).

### 2.6. The Stability of 5′-^32^P-End-Labeled “Matrix” Double-Stranded Oligonucleotides

To ensure the stability of “matrix” oligonucleotides (Control 2 and 3; Table 1), the 5′-^32^P-end-labeled double-stranded oligonucleotides (200 cps) were treated with:

- nuclear extract (NE);
- formamidopyrimidine-DNA glycosylase (FPG), which releases damaged purines from dsDNA generating 1 base gap;
- endonuclease III (Nth), which releases damaged pyrimidines from dsDNA generating 1 base gap;
- UDG, which releases uracil from ss- and dsDNA generating AP site;
- 1M piperidine, which reveals any DNA lesions;
- UDG with subsequent 1M piperidine treatment, which releases uracil from ss- and dsDNA, generating AP site and as a result of subsequent piperidine action 1 base gap.

#### 2.6.1. Treatment with Nuclear Extracts

A total of 10 μg of xrs5 NE was incubated with dsDNA in 8 μL of repair buffer (70 mM Tris-HCl (pH 7.5), 5 mM MgCl_2_, 10 mM DTT, 4 mM ATP, 40 mM phosphocreatine, 1.6 μg/mL phosphocreatine kinase, 0.1 mM dATP, 0.1 mM dCTP, 0.1 mM dGTP, and 0.1 mM dTTP) at 37 °C for 0, 1, and 120 min. After required time, reactions were stopped with 8 μL of denaturing stop solution and samples were examined on 15% denaturing polyacrylamide gel (Figure S3).

#### 2.6.2. Treatment with FGP, Nth, and UDG

Overall, 5U of FPG was incubated with dsDNA in 5 μL of reaction buffer (10 mM Bis-Tris Propane-HCl (pH 7.0), 10 mM MgCl_2_, 1 mM DTT, and 100 μg/mL BSA) at 37 °C for 0, 1 and 120 min.

A total of 5U of Nth was incubated with dsDNA in 5 μL of reaction buffer (20 mM Tris-HCl (pH 8.0), 1 mM EDTA and 1mM DTT) at 37 °C for 0, 1 and 120 min.

A total of 5U of UDG was incubated with dsDNA in 5 μL of reaction buffer (20 mM Tris-HCl (pH 8.0), 1 mM EDTA and 1mM DTT) at 37 °C for 0, 1 and 120 min.

After the required time, reactions were stopped with 5 μL of denaturing stop solution, and samples were examined on 15% denaturing polyacrylamide gel (Figure S3).

#### 2.6.3. Treatment with Piperidine

DsDNA was incubated with 100 μL of 1M piperidine at 80 °C for 30 min. After the required time, samples were precipitated with 250 μL of cold ethanol and 2 μL of glycogen (placed on dry ice, 30 min), centrifuged (13,000× *g* rpm, 4 °C, 30 min), and dried under reduced pressure at room temperature. The residues were resuspended in 5 μL of denaturing stop solution and samples were examined on 15% denaturing polyacrylamide gel (Figure S3).

#### 2.6.4. Treatment with UDG and Piperidine

A total of 5U of UDG was incubated with dsDNA in 5 μL of reaction buffer (20 mM Tris-HCl (pH 8.0), 1 mM EDTA and 1mM DTT) at 37 °C for 0, 1, and 120 min. After the required time, reactions were stopped by placing samples on ice (4 °C) and subsequently incubated with 100 μL of 1M piperidine at 80 °C for 30 min. After this time, samples were precipitated with 250 μL of cold ethanol and 2 μL of glycogen (placed on dry ice, 30 min), centrifuged (13,000× *g* rpm, 4 °C, 30 min) and dried under reduced pressure at room temperature. The residues were resuspended in 5 μL of denaturing stop solution and samples were examined on 15% denaturing polyacrylamide gel (Figure S3).

### 2.7. PAGE Electrophoresis

The reactions were stopped with denaturing stop solution (95% formamide, 2 mM EDTA, 0.025% bromophenol blue, and 0.025% xylene cyanole). Samples were subjected to electrophoresis on a 15% polyacrylamide gel containing 8M urea in 1× TBE (89 mM Tris-HCl, 89 mM boric acid, 2 mM EDTA) for 120 min at a constant power of 45 W. The results of PAGE electrophoresis were visualized by autoradiography.

### 2.8. Preparation of Nuclear Extracts

The NE was prepared from xrs5 cell line (ATCC, CRL-2348, VA, USA), Ku80 deficient (it allows the avoidance of interfering action of Ku80 binding to linear DNA termini or SSB). The cells were harvested in exponential phase, and the pelleted cells were treated using NE-PER™ Nuclear and Cytoplasmic Extraction Reagents kit (ThermoFisher Scientific, Waltham, MA, USA) according to the manufacturer’s protocol. The concentration of NE was determined using colorimetric Pierce™ 660 nm Protein Assay (ThermoFisher Scientific, Waltham, MA, USA) and was found between 3.4 and 7.0 mg/mL. Aliquots of NE were stored at −80 °C for no longer than six months.

### 2.9. Repair Assays

The 5′-^32^P-end-labeled double-stranded oligonucleotides (200 cps) were incubated with 10 μg of xrs5 NE in 8 μL of repair buffer (70 mM Tris-HCl (pH 7.5), 5 mM MgCl_2_,10 mM DTT, 4 mM ATP, 40 mM phosphocreatine, 1.6 μg/mL phosphocreatine kinase, 0.1 mM dATP, 0.1 mM dCTP, 0.1 mM dGTP, and 0.1 mM dTTP) at 37 °C for 0, 1, 5, 15, 30, 60, 90, and 120 min. The amount of NE used in repair assay was optimized from titration studies (data not shown). After required time, reactions were stopped with 8 μL of denaturing stop solution and samples were examined on 15% denaturing polyacrylamide gel.

Experiments were performed three times to confirm consistency and reliability of results and quantified using Quantity One software (Bio-Rad, Hercules, CA, USA). The time dependence of an AP site repair was analyzed as the intensity of the bands (representing ssDNA, SSB, ssDNA with one or more bases added (before ligation) or rejoined strand) and expressed as a percentage of the total intensity of all bands for one sample (each lane). As the repair activity of different batches of NE varies slightly, results for the AP site rejoining (AP site as a part of clustered damage) were compared with the control (AP site as single damage) for each experiment.

## 3. Results and Discussion

In this study, the influence of the distance between lesions within clustered DNA damage on its nuclear repair process was examined. The experimental model was synthetic double-stranded oligonucleotides with dU (as a precursor of an AP site) in one strand and 5′,8-cyclo-2′-deoxypurines in opposite strand: ScdA, RcdA, ScdG, and RcdG (Table 1). Efficient 5′-^32^P-end-labeling of single-stranded oligonucleotides and annealing to duplex was verified on native polyacrylamide gels (Figure S1). AP sites were obtained by treatment with UDG, which releases uracil from DNA due to instability of the A:::U pair. The purity of the double-stranded ^32^P-oligonucleotides containing AP sites and efficient AP sites’ formation was verified on denaturing polyacrylamide gels (Figure S2). As previously shown, ScdA and ScdG are stable during treatment with NE and chosen glycosylases [12,20]. The stability of dsDNA containing ScdA, RcdA, ScdG, and RcdG as single lesions (Control 2) was tested under the influence of NE, FPG, Nth, UDG, and UDG with subsequent 1M piperidine and 1M piperidine. To verify if no additional interactions between enzymes and dsDNA occurred, a native strand (Control 3) was also tested. It was confirmed that dsDNA containing cdPu is stable in experimental conditions up to 120 min (Figure S3). It is in agreement with previous studies that cdPus are not excised from DNA by BER machinery [20].

NE obtained from xrs5 cell line (X-ray sensitive Chinese hamster ovarian mutant cell line, Ku80 deficient) was used to study the AP sites’ repair efficiency within CDL. The activity of proteins involved in BER in NE was confirmed for the control oligonucleotide (Control 1) containing a single AP site (Table 1). Strand incision (endonuclease activity) was observed after 1 min (Table S14), while polymerase was active after 5 min (Table S15). A rejoined strand was observed after 30 min of incubation with NE, increasing with time and reaching 78.31% after 120 min (Table S13).

**Figure 3:**
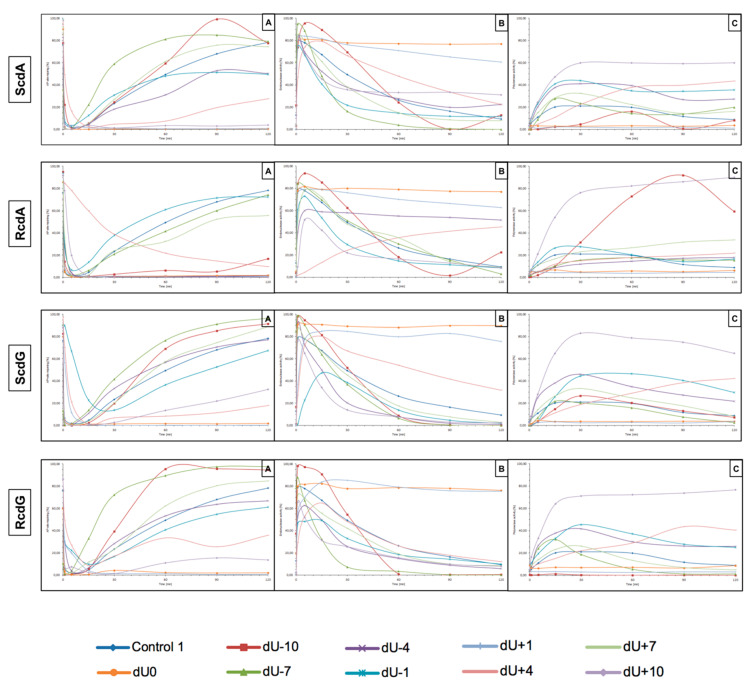
Graphical representation of DNA repair assays’ results. (**A**) AP site rejoining efficiency, (**B**) endonuclease activity, and (**C**) polymerase activity. Graphs with marked SD are presented in higher resolution in Supplementary Materials.

The presented study examines how (5′S) and (5′R) 5′,8-cyclo-2′-deoxypurines affect BER repair of clustered DNA damage in NE from xrs5 cells. Experiments were performed three times, as described in the Materials and Methods Section. DsDNA (40-mer) containing cdPu in one strand and AP site in opposite strand located 1 to 10 nucleobases (with intervals of 3 bases) in both, 3′ and 5′ direction was incubated with NE (10 μg). The distances between lesions were chosen to complete and compare results with previous studies [12,27]. As mentioned before, cdPus are not a substrate for BER. Therefore, this study focuses on the other lesion in CDL (AP site) and its incision efficiency, subsequent DNA synthesis, and strand reconstitution by nuclear BER proteins. The results are presented in Figure 3 (individual graphs and autoradiograms showing the repair are presented in Supplementary Materials).

### 3.1. The Influence of 5′,8-Cyclo-2′-Deoxyadenosine (cdA) on the BER in xrs5 Nuclear Extract

The ScdA is one of the most studied cdPu in the context of clustered DNA damage [12,20,25,32,37,38,39]. The RcdA is present in DNA in lower quantities than ScdA [21]. Interestingly, sunlight causes irreversible photoisomerization of ScdA to RcdA [40].

**Figure 4:**
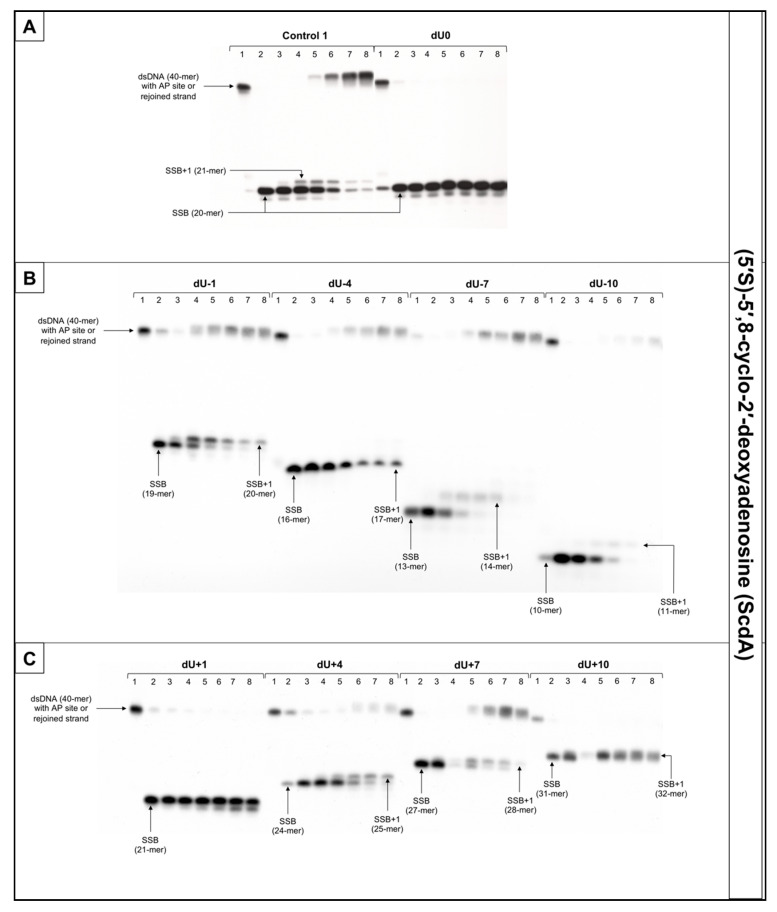
The representative autoradiograms of denaturing PAGE presenting repair of dsDNA containing clustered damage with AP site in one strand and ScdA in the opposing strand: (**A**) Controls: dsDNA with a single lesion in one strand (Control 1); dsDNA with clustered lesions in two strands opposite to each other (dU0); (**B**) dsDNA with clustered lesions in two strands where AP site is located 1–10 base pairs in 3′ direction (negative numbers); (**C**) dsDNA with clustered lesions in two strands where AP site is located 1–10 base pairs in 5′ direction (positive numbers). Each lane corresponds with different assay time: lane 1–0 min; lane 2–1 min; lane 3–5 min; lane 4–15 min; lane 5–30 min; lane 6–60 min; lane 7–90 min; lane 8–120 min. Each experiment was performed in triplicate to ensure results’ consistency.

**Figure 5:**
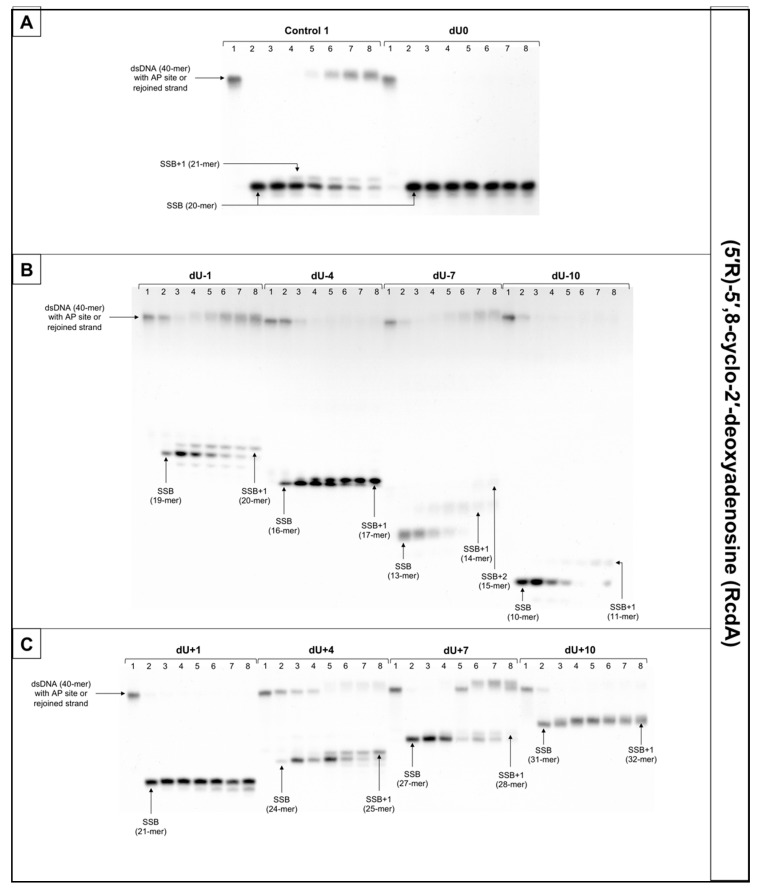
The representative autoradiograms of denaturing PAGE presenting repair of dsDNA containing clustered damage with AP site in one strand and RcdA in the opposing strand: (**A**) Controls: dsDNA with a single lesion in one strand (Control 1); dsDNA with clustered lesions in two strands opposite to each other (dU0); (**B**) dsDNA with clustered lesions in two strands where AP site is located 1–10 base pairs in 3′ direction (negative numbers); (**C**) dsDNA with clustered lesions in two strands where AP site is located 1–10 base pairs in 5′ direction (positive numbers). Each lane corresponds with different assay time: lane 1–0 min; lane 2–1 min; lane 3–5 min; lane 4–15 min; lane 5–30 min; lane 6–60 min; lane 7–90 min; lane 8–120 min. Each experiment was performed in triplicate to ensure results’ consistency.

The incision rate at which dsDNA with an AP site in one strand and cdA in the complementary strand was examined. Endonucleases present in NE produce SSBs (observed as bands corresponding to 10-mer for −10 position up to 31-mer for +10 position). SSBs were formed for all positions of AP sites after 1 min of incubation with NE (minimum of 74.5%) except ScdA/dU+4 in the case of which it took 15 min to reach 79.16%. Other incision yields differed depending on the position with the lowest of 74.5% for ScdA/dU-1 and the highest of 94.93% for ScdA/dU-7 (Figure 4, Figure S5C, and Table S2), compared to the control with 78.67% after 1 min (Table S14). The results seem to align with the fact that APE1 must be in direct contact to incise the dsDNA. It is also consistent with previous results in which the AP site in +5 position to ScdA inhibited endonuclease activity [12]. Surprisingly, in previous studies, position -5 inhibited incision, whereas here, ScdA/dU-4 showed 83.37% incision efficiency. The overall endonuclease activity for RcdA was lower than for ScdA. After 1 min of incubation with NE, substrates containing RcdA in positions 0, −1, −7, −10, +1, and +7 were incised with a minimum of 50% efficiency. RcdA/dU+10 and RcdA/dU-4 needed 5 min to reach this threshold and RcdA/dU+4 reached only 13.24% after 15 min (Figure 5, Figure S7C, Table S5). These results indicate that RcdA decreases APE1 activity stronger than ScdA. What is more, when CDL are located on the same strand in dsDNA in +1 position, APE1 is stopped by RcdA [25]. However, in the bi-stranded model of CDL, RcdA/dU+1 showed 75.49% incision efficiency after 1 min (Table S5). Another noteworthy fact is RcdA/dU-4, RcdA/dU+7 and RcdA/dU+10 incision rates were about 20–40% lower than control, while the same positions for ScdA were incised at a level comparable to Control 1 (Tables S2, S5, and S14).

The influence of cdA on polymerase activity was also considered. Polymerases play an important role in BER repairing lesions located opposite to cdPus within a cluster. Polymerase β (Polβ) bypasses RcdA during replication and repair but cannot bypass ScdA [41]. When ScdA was present, only one nucleotide was inserted indicating the action of the SP–BER mechanism for all tested substrates. Single nucleotide was incorporated with the efficiency increasing in the following order (data compared for the 30 min reaction time): −10 < +4 < −7 < +7 < −4 < −1 < +10 (Figure 4, Figure S5E, and Table S3). Interestingly, the AP site located ScdA/dU-10 showed only 4.62% incorporation efficiency after 30 min reaching its highest point at 60 min with 16.06%. However, some loss of band intensity was noted suggesting the activity of exonucleases in the NE. For the complementary lesions (ScdA/dU0) and for ScdA/dU+1 no DNA synthesis was observed (no SSB+1 bands), which is consistent with results obtained previously [12]. Polymerase activity is approximately 8–20% lower for RcdA than ScdA (Tables S3 and S6). The yield of DNA synthesis increased in the following order (data compared for the 30 min reaction time): −4 < +4 < −7 < +7 < −1 < −10 < +10 (Figure 5, Figure S7E, and Table S6). In positions 0 and +1, no incorporation was observed, which confirms that cdA in those positions blocks repair of lesion located in the opposite strand. For ScdA/dU-10, the activity of polymerase was inhibited (4.62% vs. 21.06% for Control 1). However, for RcdA/dU-10, incorporation efficiency reaches 42.48% after 30 min and 91.26% after 120 min (Table S6). These results seem to align with the ability of Polβ to bypass RcdA, but not ScdA. It is also noteworthy that, in individual experiments, the presence of more than one band was observed for RcdA/dU-7 and RcdA/dU+10 (Figure S6). Bands corresponding to SSB+2 resulting from the polymerase activity suggest the involvement of the LP–BER. However, this phenomenon occurred randomly and was not replicated.

The strand reconstitution was not observed for positions 0 and +1 for both isomers of cdA, which is consistent with previous studies [12]. ScdA/dU-1 and RcdA/dU-1 were repaired by NE on the control level (47.96% and 60.96% after 60 min, respectively, vs. 49.4% for Control 1), which contradicts past results (Figure S8 and Tables S1 and S4) [12]. Moreover, ScdA/dU+10 showed only 3.43% rejoining efficiency after 60 min, while ScdA/dU-10 showed 59.42% (Table S1). For the ScdA, the strand rejoining efficiency was found to increase in the following order (data compared for 60 min reaction time): +4 < −4 < −1 < −10 < +7 < −7 (Figure S5A and Table S1). The situation is different when RcdA is present in complementary strand—the reconstitution was not observed for 0, +1, −4, and +10, while the rejoining efficiency increased in the following order (data compared for 60 min reaction time): +4 < +7 < −7 < −1 (Figure 5, Figure S7A, and Table S4). The RcdA/dU-10 showed only 6.23% rejoining efficiency after 60 min, which is lower than ScdA (59.42%) (Tables S1 and S4). It may be a consequence of inhibited polymerase activity, which was not able to insert a nucleotide, thus preventing the final step of BER (strand ligation). On the other hand, for lesions located 10 bases in the 5′ direction from cdA, the BER pathway was not activated. While polymerase activity was the highest, in this case, the descending strand (7-mer) could have dissociated from the template leaving space for the polymerase to act but preventing strand rejoining. ScdA/dU-7 and ScdA/dU+7 are repaired with the highest rate (81.13% and 61.04%, respectively), which seems consistent with past studies in which -8 and +8 positions were explored [12]. The trend is also correct for RcdA—RcdA/dU-7 and RcdA/dU+7 are repaired with one of the highest rates (41.48% and 32.38%, respectively). Surprisingly, ScdA/dU-4 was repaired with efficiency higher approximately four-fold than ScdA/dU+4, while for 5′R isomer, the trend was inversed—RcdA/dU+4 was repaired in 21,84% after 60 min, and no strand reconstitution was observed for RcdA/dU-4.

Lesions located in the 5′ direction (positive numbers) from cdA (5′R and 5′S isomer) were repaired less efficiently than those located in the 3′ direction (negative numbers). Nevertheless, RcdA showed overall activity of BER 30–50% lower for the majority of tested substrates (except RcdA/dU-1 and RcdA/dU+4).

### 3.2. The Influence of 5′,8-Cyclo-2′-deoxyguanosine (cdG) on the BER in xrs5 Nuclear Extract

From previous studies, it is known that ScdG blocks replication and repair in *Escherichia coli* due to the inability of the polymerase to bypass its complex structure and inefficient action of NER [20,42]. RcdG is less investigated due to the problems during its synthesis and incorporation into model oligonucleotides [20]. Therefore, studies exploring the impact of cdG located within CDL on the repair of the other lesion by BER are highly demanded. Diastereomers of cdA influence the repair differently; therefore, it may be assumed that 5′S and 5′R cdG also impacts BER in a different manner.

**Figure 6:**
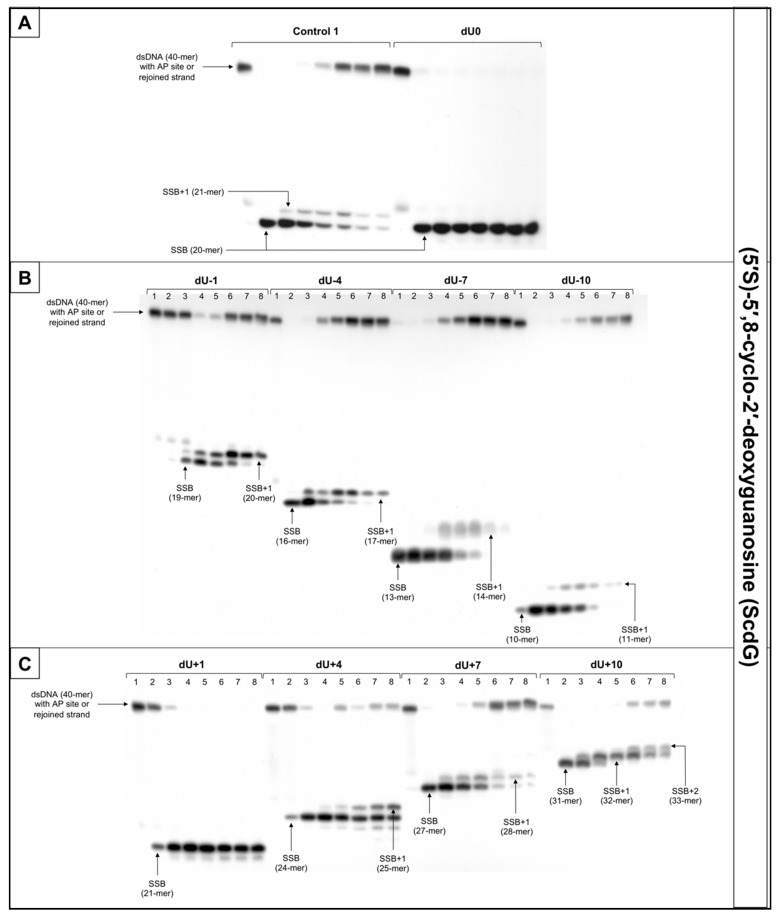
The representative autoradiograms of denaturing PAGE presenting repair of dsDNA containing clustered damage with AP site in one strand and ScdG in the opposing strand: (**A**) Controls: dsDNA with a single lesion in one strand (Control 1); dsDNA with clustered lesions in two strands opposite to each other (dU0); (**B**) dsDNA with clustered lesions in two strands where AP site is located 1–10 base pairs in 3′ direction (negative numbers); (**C**) dsDNA with clustered lesions in two strands where AP site is located 1–10 base pairs in 5′ direction (positive numbers). Each lane corresponds with different assay time: lane 1–0 min; lane 2–1 min; lane 3–5 min; lane 4–15 min; lane 5–30 min; lane 6–60 min; lane 7–90 min; lane 8–120 min. Each experiment was performed in triplicate to ensure results’ consistency.

**Figure 7:**
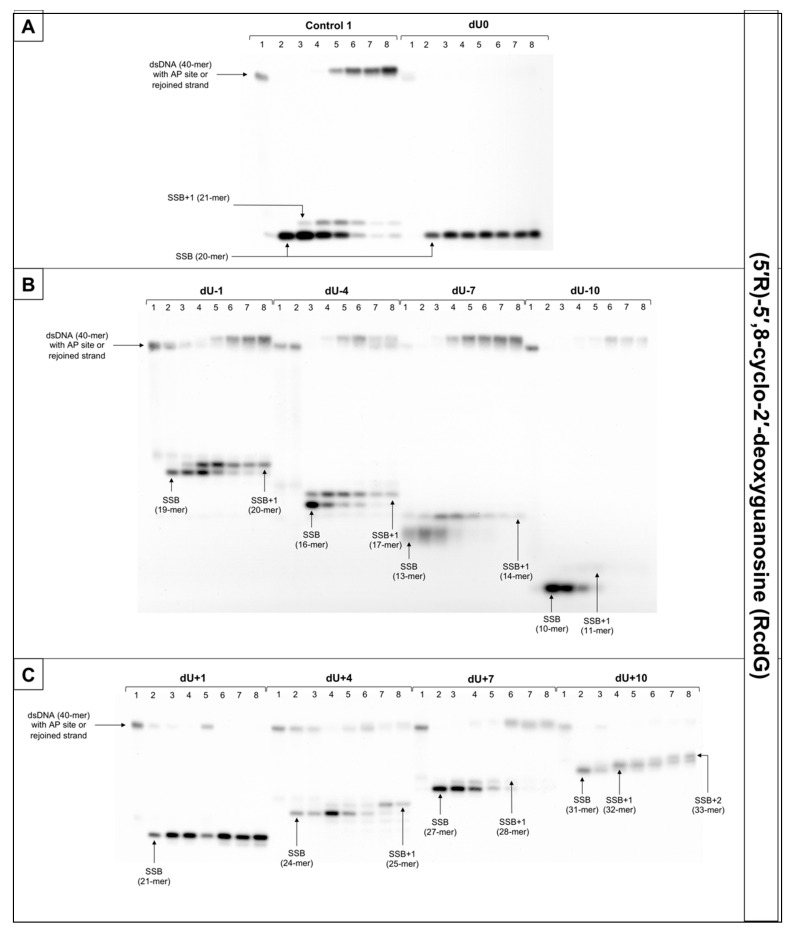
The representative autoradiograms of denaturing PAGE presenting repair of dsDNA containing clustered damage with AP site in one strand and RcdG in the opposing strand: (**A**) Controls: dsDNA with a single lesion in one strand (Control 1); dsDNA with clustered lesions in two strands opposite to each other (dU0); (**B**) dsDNA with clustered lesions in two strands where AP site is located 1–10 base pairs in 3′ direction (negative numbers); (**C**) dsDNA with clustered lesions in two strands where AP site is located 1–10 base pairs in 5′ direction (positive numbers). Each lane corresponds with different assay time: lane 1–0 min; lane 2–1 min; lane 3–5 min; lane 4–15 min; lane 5–30 min; lane 6–60 min; lane 7–90 min; lane 8–120 min. Each experiment was performed in triplicate to ensure results’ consistency.

For that reason, this study examined dsDNA with an AP site in one strand and cdG in opposing strand and its influence on the main steps of the BER mechanism—incision, DNA synthesis, and strand rejoining. SSBs formed as a result of the endonucleolytic activity of NE (observed as bands corresponding to 10-mer for −10 position up to 31-mer for +10 position) were formed for the majority of substrate oligonucleotides after 1 min of incubation with NE. SSB formation rate differed depending on the interlesion distance with the lowest of 76.09% for ScdG/dU+10 and the highest of 98.9% for ScdG/dU-7 (Figure 6, Figure S10C, and Table S8), compared to Control 1 with 78.67% after 1 min (Table S14). However, for ScdG/dU+4, ScdG/dU+1 and ScdG/dU-1 endonucleases were less active—the highest incision yield was reached after 15 min (Table S8). The overall endonuclease activity for RcdG was lower than for ScdG. After 1 min incubation with NE substrates containing RcdG in positions 0, −4, −7, −10, +1, +7, and +10 were incised with a minimum of 50% efficiency (Figure 7, Figure S12C, and Table S11). RcdG/dU-1 reached 46.6% after 1 min and a maximum of 49.18% after 15 min while RcdG/dU+4 reached only 29.25% after 1 min peaking at 15 min with 64.87%. Similar to cdA, results indicate that RcdG has a stronger inhibitory effect on the activity of APE1. Interestingly, the incision of both diastereomers in positions −1, +1, and +4 was reduced compared to Control 1 with a single lesion, the same as for cdA. On the other hand, RcdG/dU-4 showed endonuclease activity about 25% lower than the Control 1 for 5′R isomer and about 10% higher than the Control 1 for 5′S isomer, which was also observed for cdA. APE1 was inhibited more by RcdG/dU+7 and RcdG/dU +10, compared to their corresponding substrates with ScdG.

For ScdG, polymerase activity was observed with the efficiency increasing in the following order (data compared for the 30 min reaction time): +4 < −7 < −10 < +7 < −1 < −4 < +10 (Figure 6, Figure S10E, and Table S9). For RcdG, the yield of DNA synthesis increased in the following order (data compared for the 30 min reaction time): −7 < +4 < +7 < −4 < −1 < +10 (Figure 7, Figure S12E, and Table S12). In positions 0 and +1, no DNA synthesis was observed (no SSB+1 bands). It is consistent throughout this study and confirms that both diastereomers of cdG in those positions block polymerases. Polymerase activity is comparable for RcdG and ScdG containing DNA with lesions in corresponding positions (Tables S9 and S12). Conversely, AP site located RcdG/dU-10 showed no SSB+1 while for ScdG/dU-10 activity of polymerase was at control level (26.62% vs. 21.06% for Control 1) (Tables S9, S12, and S15). Polβ, which can bypass RcdA and not ScdA seems to show an inversed affinity for cdG. Moreover, the SSB+2 bands were observed for RcdG and ScdG/dU+10 (Figure 6 and Figure 7) and the polymerase activity was the highest (71.09% and 82.94%, respectively). In the case of cdA, this phenomenon occurred randomly, but cdG showed consistent activity of the LP–BER for lesions located +10 bases from cdG. Additionally, SSB+2 was observed for RcdG/dU-4, but it was incidental (Figure S11). As suggested for cdA, the descending strand (7-mer) could have dissociated from the template leaving space for the polymerase to incorporate more than one nucleotide. However, no strand rejoining was observed for cdA but when cdG was present, DNA was reconstituted to some extent.

The repair activity was not observed for positions 0 and +1 for both isomers of cdG. ScdG/dU-1 and RcdG/dU-1 showed strand rejoining by BER enzymes in NE with a lower rate than the control (36.52% and 40.72% after 60 min, respectively, vs. 49.4% for Control 1) (Figure S11 and Tables S7 and S10), which is consistent with results for cdA. For the ScdG, the DNA reconstitution efficiency was found to increase in the following order (data compared for 60-min reaction time): +4 < +10 <−1 < −4 < +7 < −10 < −7 (Figure S10A and Table S7). The situation was similar when RcdG was present in complementary strand—the rejoining efficiency increased in the following order (data compared for 60-min reaction time): +10 < +4 < −1 < −4 < +7 < −7 < −10 (Figure 7, Figure S12A, and Table S10). Moreover, ScdG/dU+10 and RcdG/dU+10 showed similarly low strand rejoining after 60 min, but after 120 min, repair level was two-fold higher for 5′S (Figure S13). The RcdG/dU-10 showed higher rejoining efficiency after 60 min (95.42%) than ScdG/dU-10 (68.92%), which is contrary to what was observed for cdA. Surprisingly, cdG showed an inversed trend for the majority of tested substrates—a higher level of repair was observed for RcdG than ScdG (for positions −1, −7, −10, +4, +7) (Figure S13), but at the same time, ScdA showed higher strand rejoining rate than RcdA (for positions −4, −7, −10, +4, +7, +10) (Figure S8).

What is important, the lesions located in the 5′ direction (positive numbers) from cdG (5′R and 5′S isomer) were repaired less efficiently than those located in the 3′ direction (negative numbers), which was also observed for cdA.

## 4. Conclusions

The cell has to efficiently repair numerous DNA lesions forming as a result of endo- and exogenous factors, e.g., radiation, metabolites, pollutants, etc. [43]. Approximately 3 × 10^17^ lesions appear every hour in the human body [25]. Therefore, it is crucial for the survival of the whole organism to recognize and repair DNA damage when it occurs. Clustered DNA damage is a particular type of damage when two or more lesions are present within 1–2 helical turns. Moreover, this type of DNA damage is a characteristic feature of ionizing radiation’s impact on genetic material. Due to their complex structures, cdPus influence DNA conformation (increased rigidity) [12]. Subsequently, the other lesions in CDL are less susceptible to the action of BER [12,25]. Presented findings complement previous studies in the field and show that the process of CDL repair needs further exploration.

This study shows that the efficient repair (including DNA incision, gap filling, and final strand reconstitution) of CDL containing cdPu depends on the distance and the relative position between lesions. Moreover, differences in the BER activity were observed between 5′S and 5′R diastereomers and between cdA and cdG lesions.

The results of the presented study were as follows:

- APE1 is active for bi-stranded CDL containing two lesions (cdPu and AP site) distanced up to 10 bases in 3′ and 5′ direction;
- incision efficiency of the repaired strand (containing AP site) is lower when RcdA or RcdG is present in dsDNA comparing to ScdA or ScdG for which incision was comparable or higher than Control 1 (containing single lesion);
- when a gap is located opposite to cdPus (position 0) or 1 base in the 5′-end direction (position +1), polymerase activity is blocked; hence, no subsequent repair is observed;
- the repair is more efficient for 5′S than 5′R diastereomers of both cdPus;
- strand reconstitution is reduced for gaps located ≤10 base pairs on the 5′-end side of cdPus, compared to ones located ≤10 base pairs on the 3′-end side of cdPu.

The one nucleotide shift of relative position of lesions within a cluster may change the course of repair or stop the process completely. It is of high importance to study those mechanisms in more detail. Additionally, not only the type and relative position of lesions matters, but also their orientation toward each other or more complex cellular structures (e.g., histones) [27]. Further studies are highly demanded to fully understand the repair of CDL, which is crucial in the context of radio- and chemotherapy. Moreover, in the future, cdPus may serve as therapeutics (e.g., in cancer treatment) or as a diagnostic tool for oxidatively induced damage detection.

## Figures and Tables

**Table 1:** The sequences of double-stranded substrate oligonucleotides containing 2′-deoxyuridine (dU) and 5′,8-cyclo-2′-deoxypurines (cdPus).

Oligonucleotide		Sequence
S/RcdA	Control 1	5 ′ -CTCTTGTCAGGAATATTGTC**U**CTATGCTCCCACCAAAGGC-3 ′ 3 ′ -GAGAACAGTCCTTATAACAGAGATACGAGGGTGGTTTCCG-5 ′
	Control 2	5 ′ -GCCTTTGGTGGGAGCATAG**X**GACAATATTCCTGACAAGAG-3 ′ 3 ′ -CGGAAACCACCCTCGTATCTCTGTTATAAGGACTGTTCTC-5 ′
	Control 3	5 ′ -CTCTTGTCAGGAATATTGTCTCTATGCTCCCACCAAAGGC-3 ′ 3 ′ -GAGAACAGTCCTTATAACAG**X**GATACGAGGGTGGTTTCCG-5 ′
	dU −10	5 ′ -CTCTTGTCAG**U**AATATTGTCTCTATGCTCCCACCAAAGGC-3 ′ 3 ′ -GAGAACAGTCCTTATAACAG**X**GATACGAGGGTGGTTTCCG-5 ′
	dU −7	5 ′ -CTCTTGTCAGGAA**U**ATTGTCTCTATGCTCCCACCAAAGGC-3 ′ 3 ′ -GAGAACAGTCCTTATAACAG**X**GATACGAGGGTGGTTTCCG-5 ′
	dU −4	5 ′ -CTCTTGTCAGGAATAT**U**GTCTCTATGCTCCCACCAAAGGC-3 ′ 3 ′ -GAGAACAGTCCTTATAACAG**X**GATACGAGGGTGGTTTCCG-5 ′
	dU −1	5 ′ -CTCTTGTCAGGAATATTGT**U**TCTATGCTCCCACCAAAGGC-3 ′ 3 ′ -GAGAACAGTCCTTATAACAG**X**GATACGAGGGTGGTTTCCG-5 ′
	dU 0	5 ′ -CTCTTGTCAGGAATATTGTC**U**CTATGCTCCCACCAAAGGC-3 ′ 3 ′ -GAGAACAGTCCTTATAACAG**X**GATACGAGGGTGGTTTCCG-5 ′
	dU +1	5 ′ -CTCTTGTCAGGAATATTGTCT**U**TATGCTCCCACCAAAGGC-3 ′ 3 ′ -GAGAACAGTCCTTATAACAG**X**GATACGAGGGTGGTTTCCG-5 ′
	dU +4	5 ′ -CTCTTGTCAGGAATATTGTCTCTA**U**GCTCCCACCAAAGGC-3 ′ 3 ′ -GAGAACAGTCCTTATAACAG**X**GATACGAGGGTGGTTTCCG-5 ′
	dU +7	5 ′ -CTCTTGTCAGGAATATTGTCTCTATGC**U**CCCACCAAAGGC-3 ′ 3 ′ -GAGAACAGTCCTTATAACAG**X**GATACGAGGGTGGTTTCCG-5 ′
	dU +10	5 ′ -CTCTTGTCAGGAATATTGTCTCTATGCTCC**U**ACCAAAGGC-3 ′ 3 ′ -GAGAACAGTCCTTATAACAG**X**GATACGAGGGTGGTTTCCG-5 ′
S/RcdG	Control 1	5 ′ -CTCTTGTCAGGAATATTGTC**U**CTATGCTCCCACCAAAGGC-3 ′ 3 ′ -GAGAACAGTCCTTATAACAGAGATACGAGGGTGGTTTCCG-5 ′
	Control 2	5 ′ -GCCTTTGGTGGGAGCATAG**Y**GACAATATTCCTGACAAGAG-3 ′ 3 ′ -CGGAAACCACCCTCGTATCTCTGTTATAAGGACTGTTCTC-5 ′
	Control 3	5 ′ -CTCTTGTCAGGAATATTGTCTCTATGCTCCCACCAAAGGC-3 ′ 3 ′ -GAGAACAGTCCTTATAACAG**Y**GATACGAGGGTGGTTTCCG-5 ′
	dU −10	5 ′ -CTCTTGTCAG**U**AATATTGTCCCTATGCTCCCACCAAAGGC-3 ′ 3 ′ -GAGAACAGTCCTTATAACAG**Y**GATACGAGGGTGGTTTCCG-5 ′
	dU −7	5 ′ -CTCTTGTCAGGAA**U**ATTGTCCCTATGCTCCCACCAAAGGC-3 ′ 3 ′ -GAGAACAGTCCTTATAACAG**Y**GATACGAGGGTGGTTTCCG-5 ′
	dU −4	5 ′ -CTCTTGTCAGGAATAT**U**GTCCCTATGCTCCCACCAAAGGC-3 ′ 3 ′ -GAGAACAGTCCTTATAACAG**Y**GATACGAGGGTGGTTTCCG-5 ′
	dU −1	5 ′ -CTCTTGTCAGGAATATTGT**U**CCTATGCTCCCACCAAAGGC-3 ′ 3 ′ -GAGAACAGTCCTTATAACAG**Y**GATACGAGGGTGGTTTCCG-5 ′
	dU 0	5 ′ -CTCTTGTCAGGAATATTGTC**U**CTATGCTCCCACCAAAGGC-3 ′ 3 ′ -GAGAACAGTCCTTATAACAG**Y**GATACGAGGGTGGTTTCCG-5 ′
	dU +1	5 ′ -CTCTTGTCAGGAATATTGTCC**U**TATGCTCCCACCAAAGGC-3 ′ 3 ′ -GAGAACAGTCCTTATAACAG**Y**GATACGAGGGTGGTTTCCG-5 ′
	dU +4	5 ′ -CTCTTGTCAGGAATATTGTCCCTA**U**GCTCCCACCAAAGGC-3 ′ 3 ′ -GAGAACAGTCCTTATAACAG**Y**GATACGAGGGTGGTTTCCG-5 ′
	dU +7	5 ′ -CTCTTGTCAGGAATATTGTCCCTATGC**U**CCCACCAAAGGC-3 ′ 3 ′ -GAGAACAGTCCTTATAACAG**Y**GATACGAGGGTGGTTTCCG-5 ′
	dU +10	5 ′ -CTCTTGTCAGGAATATTGTCCCTATGCTCC**U**ACCAAAGGC-3 ′ 3 ′ -GAGAACAGTCCTTATAACAG**Y**GATACGAGGGTGGTTTCCG-5 ′

U—represents 2′-deoxyuridine as an AP site (after treatment with UDG; see 2.5); X—represents (5′S)-5′,8-cyclo-2′-deoxyadenosine (ScdA) or (5′R)-5′,8-cyclo-2′-deoxyadenosine (RcdA); Y—represents (5′S)-5′,8-cyclo-2′-deoxyguanosine (ScdG) or (5′R)-5′,8-cyclo-2′-deoxyguanosine (RcdG).

**Table 2:** The calculated and found masses of chosen substrate oligonucleotides.

Oligonucleotide	Calculated Mass	Found Mass
Control 1 (dU strand)	12,167.90	12,168.25
Control 1 (native strand)	12,181.98	12,182.42
Mtx-ScdA	12,407.00	12,408.30
Mtx-RcdA	12,407.00	12,407.30
Mtx-ScdG	12,423.00	12,423.30
Mtx-RcdG	12,423.00	12,424.00

## Data Availability

Not applicable.

## Supplementary Materials

The following are available online at https://www.mdpi.com/2073-4409/10/4/725/s1, Figure S1: Verification of efficient annealing of single-stranded (ssDNA) and double-stranded (dsDNA) oligonucleotides on the 15% native polyacrylamide gel, Figure S2: Verification of AP sites stability/purity and AP sites’ formation by APE1 treatment (SSBs) on the 15% denaturing polyacrylamide gel, Figure S3: The stability of “matrix” oligonucleotides (Control 2 and Control 3) after treatment with FPG, Nth, NE, 1M piperidine, UDG, and UDG with subsequent 1M piperidine, Figure S4: The autoradiograms of denaturing PAGE presenting repair of dsDNA containing clustered damage with AP site in one strand and ScdA in the opposing strand—three repeats, Figure S5: Graphical representation of DNA repair assays’ results for ScdA, Figure S6: The autoradiograms of denaturing PAGE presenting repair of dsDNA containing clustered damage with AP site in one strand and RcdA in the opposing strand—three repeats, Figure S7: Graphical representation of DNA repair assays’ results for RcdA, Figure S8: AP site rejoining [%] of ScdA vs. RcdA—comparison of individual strands, Figure S9: The autoradiograms of denaturing PAGE presenting repair of dsDNA containing clustered damage with AP site in one strand and ScdG in the opposing strand—three repeats, Figure S10: Graphical representation of DNA repair assays’ results for ScdG, Figure S11: The autoradiograms of denaturing PAGE presenting repair of dsDNA containing clustered damage with AP site in one strand and RcdG in the opposing strand—three repeats, Figure S12: Graphical representation of DNA repair assays’ results for RcdG, Figure S13: AP site rejoining [%] of ScdG vs. RcdG—comparison of individual strands, Figure S14: Mass spectra of substrate oligonucleotides containing cdPu, Table S1: AP site rejoining—ScdA. Raw numerical data of densitometry, Table S2: Endonuclease activity—ScdA. Raw numerical data of densitometry, Table S3: Polymerase activity—ScdA. Raw numerical data of densitometry, Table S4: AP site rejoining—RcdA. Raw numerical data of densitometry, Table S5: Endonuclease activity—RcdA. Raw numerical data of densitometry, Table S6: Polymerase activity—RcdA. Raw numerical data of densitometry, Table S7: AP site rejoining—ScdG. Raw numerical data of densitometry, Table S8: Endonuclease activity—ScdG. Raw numerical data of densitometry, Table S9: Polymerase activity—ScdG. Raw numerical data of densitometry, Table S10: AP site rejoining—RcdG. Raw numerical data of densitometry, Table S11: Endonuclease activity—RcdG. Raw numerical data of densitometry, Table S12: Polymerase activity—RcdG. Raw numerical data of densitometry.
