# Supplementary material for: How (5′S) and (5′R) 5′,8-Cyclo-2′-Deoxypurines Affect Base Excision Repair of Clustered DNA Damage in Nuclear Extracts of xrs5 Cells? A Biochemical Study
Source: Cells. 2021 Mar 24;10(4):725. doi: 10.3390/cells10040725 (PMC8064110; doi:10.3390/cells10040725)
Supplement: Supplementary file 1 [file cells-10-00725-s001.pdf]

How (5′S) and (5′R) 5′,8-cyclo-2′-deoxypurines affect base excision repair of clustered DNA damage in nuclear extracts of xrs5 cells? The biochemical study.

Supplementary Materials

Page	Item	Title
2	**Figure S1**	Verification of efficient annealing of single-stranded (ssDNA) and double-stranded (dsDNA) oligonucleotides on the 15% native polyacrylamide gel
3-4	**Figure S2**	Verification of AP sites stability/purity and AP sites' formation by APE1 treatment (SSBs) on the 15% denaturing polyacrylamide gel
5	**Figure S3**	The stability of „matrix" oligonucleotides (Control 2 and Control 3) after treatment with FPG, Nth, NE, 1M piperidine, UDG, UDG with subsequent 1M piperidine
6-8	**Figure S4**	The autoradiograms of denaturing PAGE presenting repair of dsDNA containing clustered damage with AP site in one strand and **ScdA** in the opposing strand – 3 repeats
9-11	**Figure S5**	Graphical representation of DNA repair assays' results for ScdA
12	**Table S1**	AP site rejoining - ScdA. Raw numerical data of densitometry
13	**Table S2**	Endonuclease activity - ScdA. Raw numerical data of densitometry
14	**Table S3**	Polymerase activity - ScdA. Raw numerical data of densitometry
15-17	**Figure S6**	The autoradiograms of denaturing PAGE presenting repair of dsDNA containing clustered damage with AP site in one strand and R**cdA** in the opposing strand – 3 repeats
18-20	**Figure S7**	Graphical representation of DNA repair assays' results for RcdA
21	**Table S4**	AP site rejoining - RcdA. Raw numerical data of densitometry
22	**Table S5**	Endonuclease activity - RcdA. Raw numerical data of densitometry
23	**Table S6**	Polymerase activity - RcdA. Raw numerical data of densitometry
24-27	**Figure S8**	AP site rejoining [%] of ScdA vs. RcdA – comparison of individual strands
28-30	**Figure S9**	The autoradiograms of denaturing PAGE presenting repair of dsDNA containing clustered damage with AP site in one strand and **ScdG** in the opposing strand – 3 repeats
31-33	**Figure S10**	Graphical representation of DNA repair assays' results for ScdG
34	**Table S7**	AP site rejoining - ScdG. Raw numerical data of densitometry
35	**Table S8**	Endonuclease activity - ScdG. Raw numerical data of densitometry
36	**Table S9**	Polymerase activity - ScdG. Raw numerical data of densitometry
37-39	**Figure S11**	The autoradiograms of denaturing PAGE presenting repair of dsDNA containing clustered damage with AP site in one strand and R**cdG** in the opposing strand – 3 repeats
40-42	**Figure S12**	Graphical representation of DNA repair assays' results for RcdG
43	**Table S10**	AP site rejoining - RcdG. Raw numerical data of densitometry
44	**Table S11**	Endonuclease activity - RcdG. Raw numerical data of densitometry
45	**Table S12**	Polymerase activity - RcdG. Raw numerical data of densitometry
46-49	**Figure S13**	AP site rejoining [%] of ScdG vs. RcdG – comparison of individual strands
50	**Table S13**	AP site rejoining – Control 1. Raw numerical data of densitometry
50	**Table S14**	Endonuclease activity - Control 1. Raw numerical data of densitometry
51	**Table S15**	Polymerase activity - Control 1. Raw numerical data of densitometry
52-54	**Figure S14**	Mass spectra of substrate oligonucleotides containing cdPu

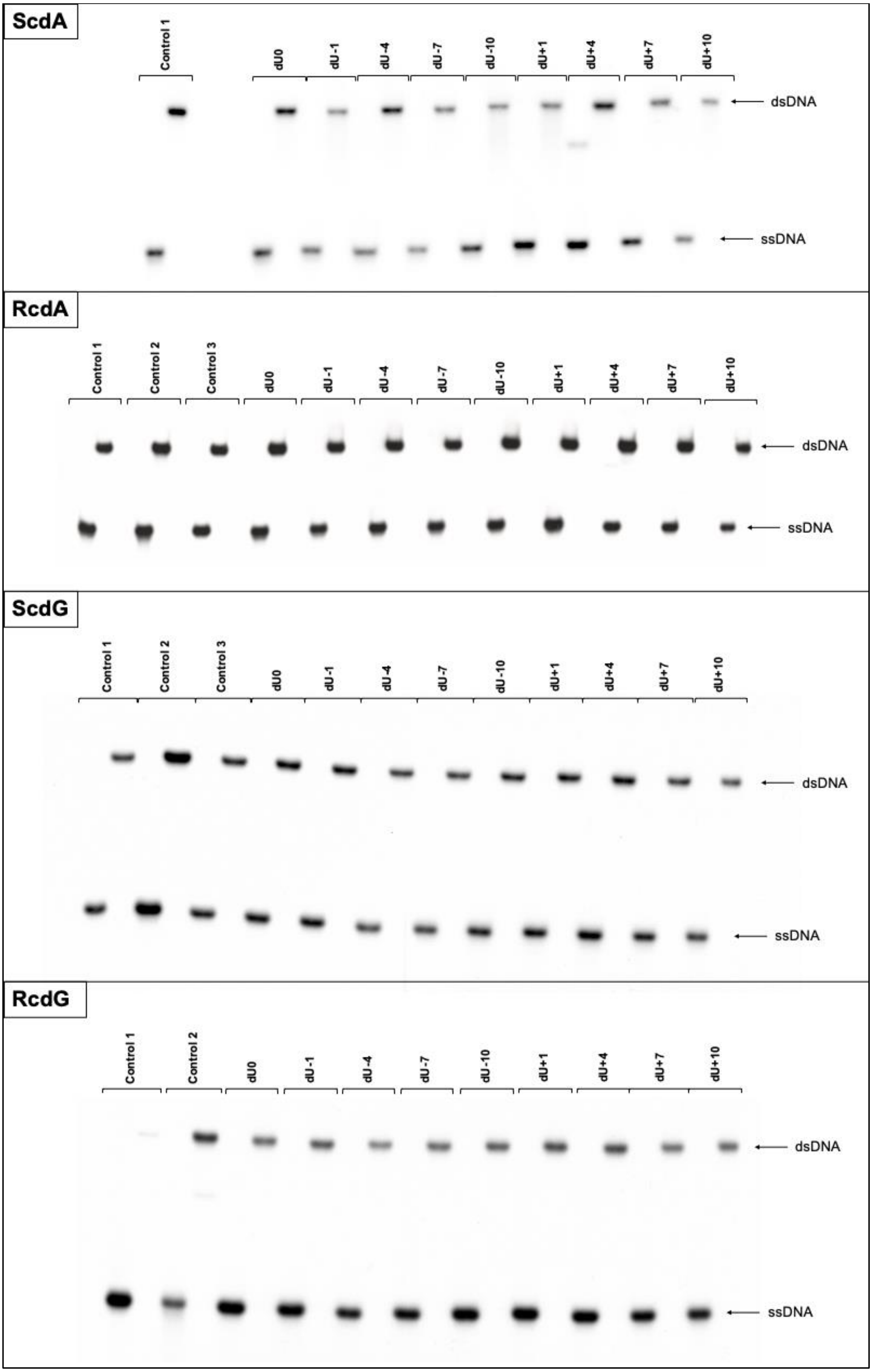

Figure S1. Verification of efficient annealing of single-stranded (ssDNA) and double-stranded (dsDNA) oligonucleotides on the 15% native polyacrylamide gel

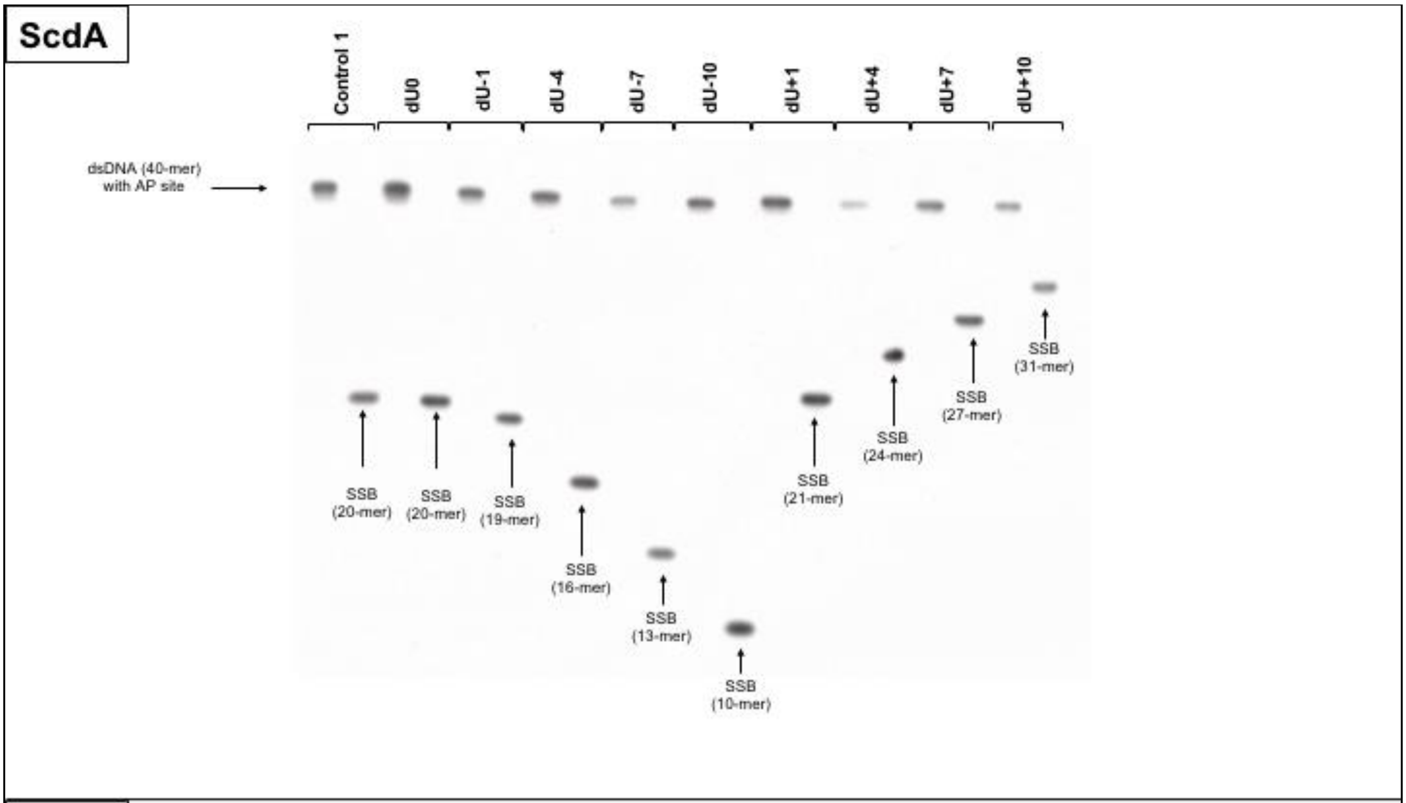
ScdA
Control 1
dU0
dU-1
dU-4
dU-7
dU-10
dU+1
dU+4
dU+7
dU+10
dsDNA (40-mer) with AP site
SSB (20-mer)
SSB (20-mer)
SSB (19-mer)
SSB (16-mer)
SSB (13-mer)
SSB (10-mer)
SSB (21-mer)
SSB (24-mer)
SSB (27-mer)
SSB (31-mer)

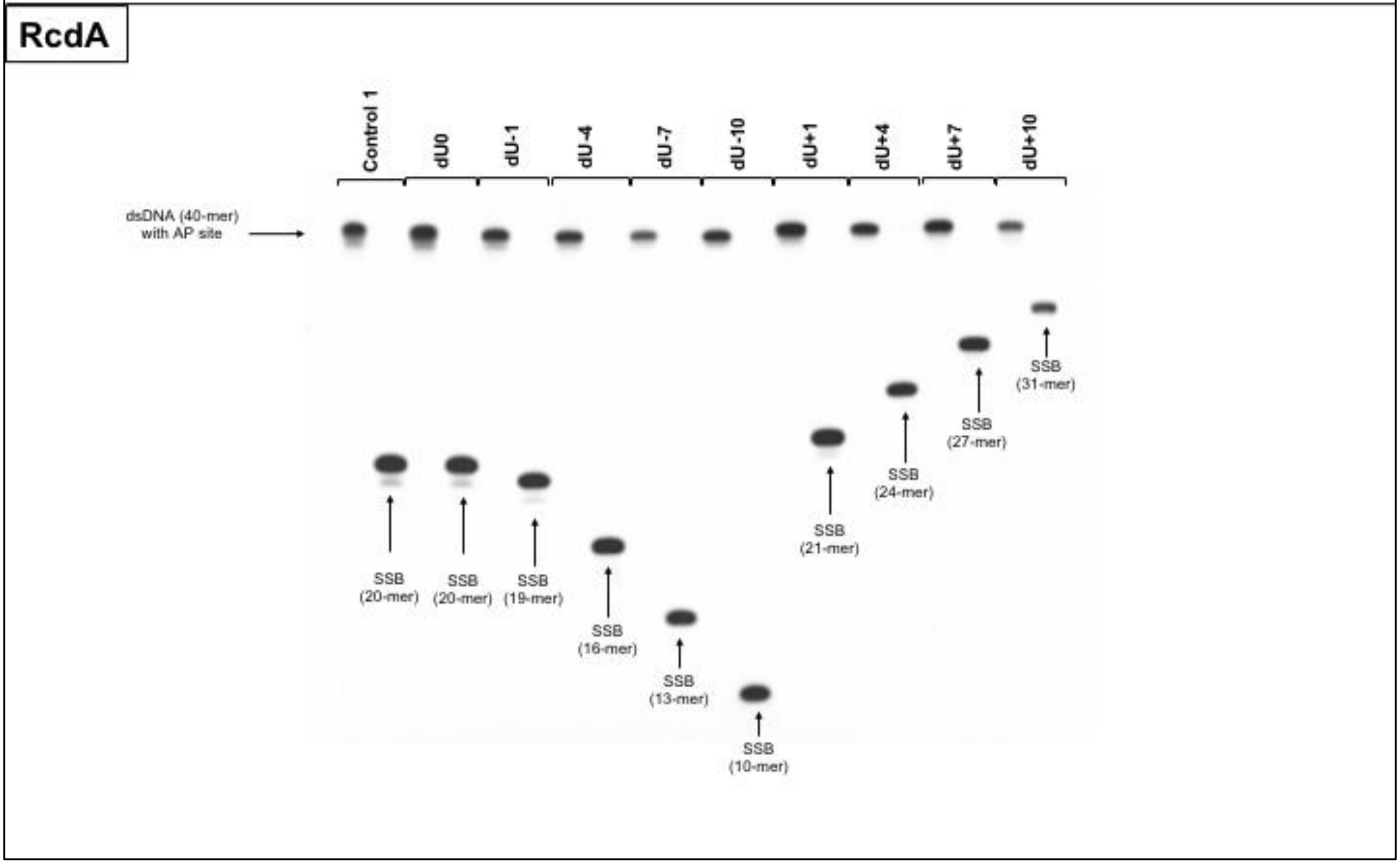
RcdA
Control 1
dU0
dU-1
dU-4
dU-7
dU-10
dU+1
dU+4
dU+7
dU+10
dsDNA (40-mer) with AP site
SSB (20-mer)
SSB (20-mer)
SSB (19-mer)
SSB (16-mer)
SSB (13-mer)
SSB (10-mer)
SSB (21-mer)
SSB (24-mer)
SSB (27-mer)
SSB (31-mer)

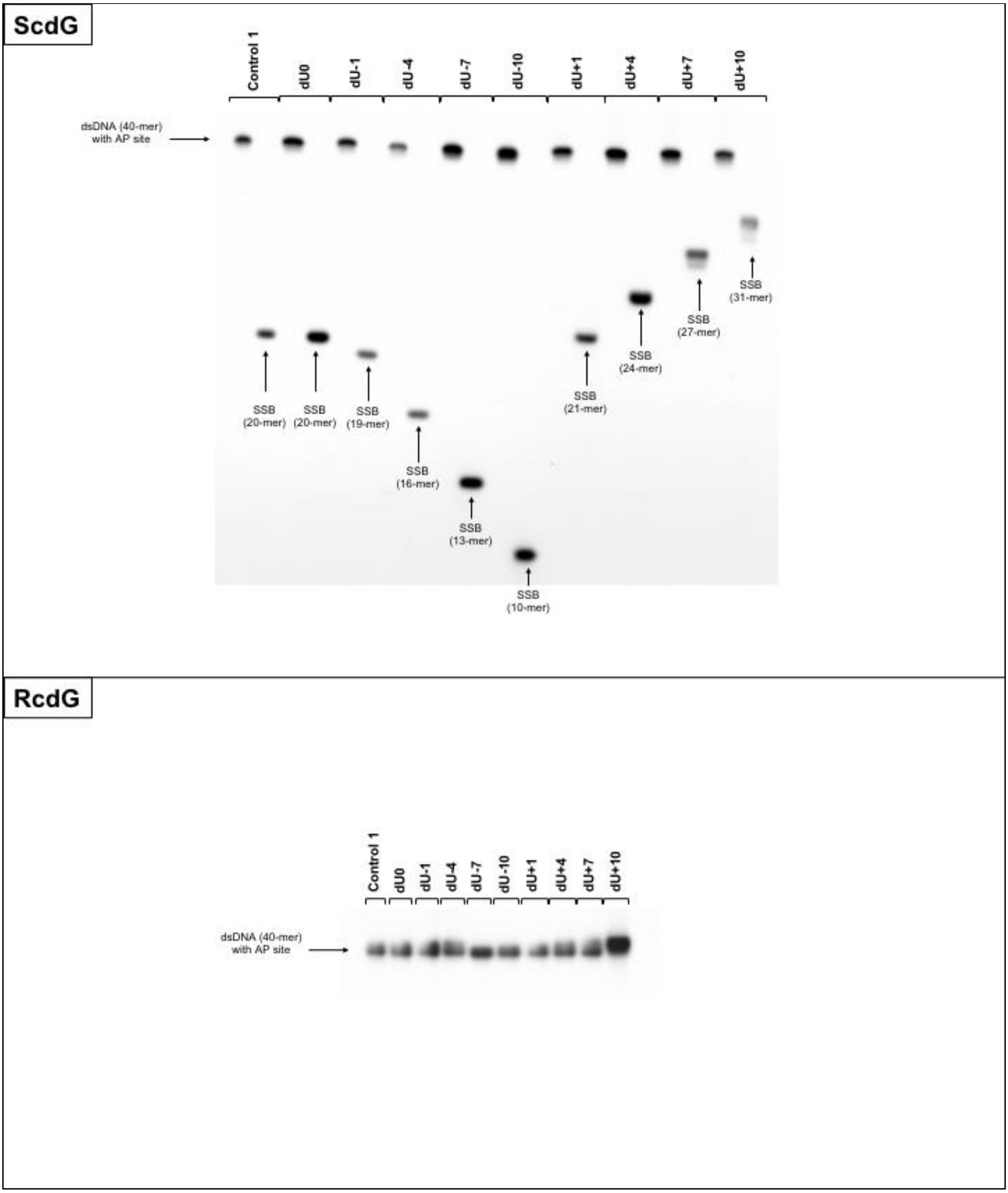

Figure S2. Verification of AP sites' stability/purity and AP sites' formation by APE1 treatment (SSBs) on the 15% denaturing polyacrylamide gel

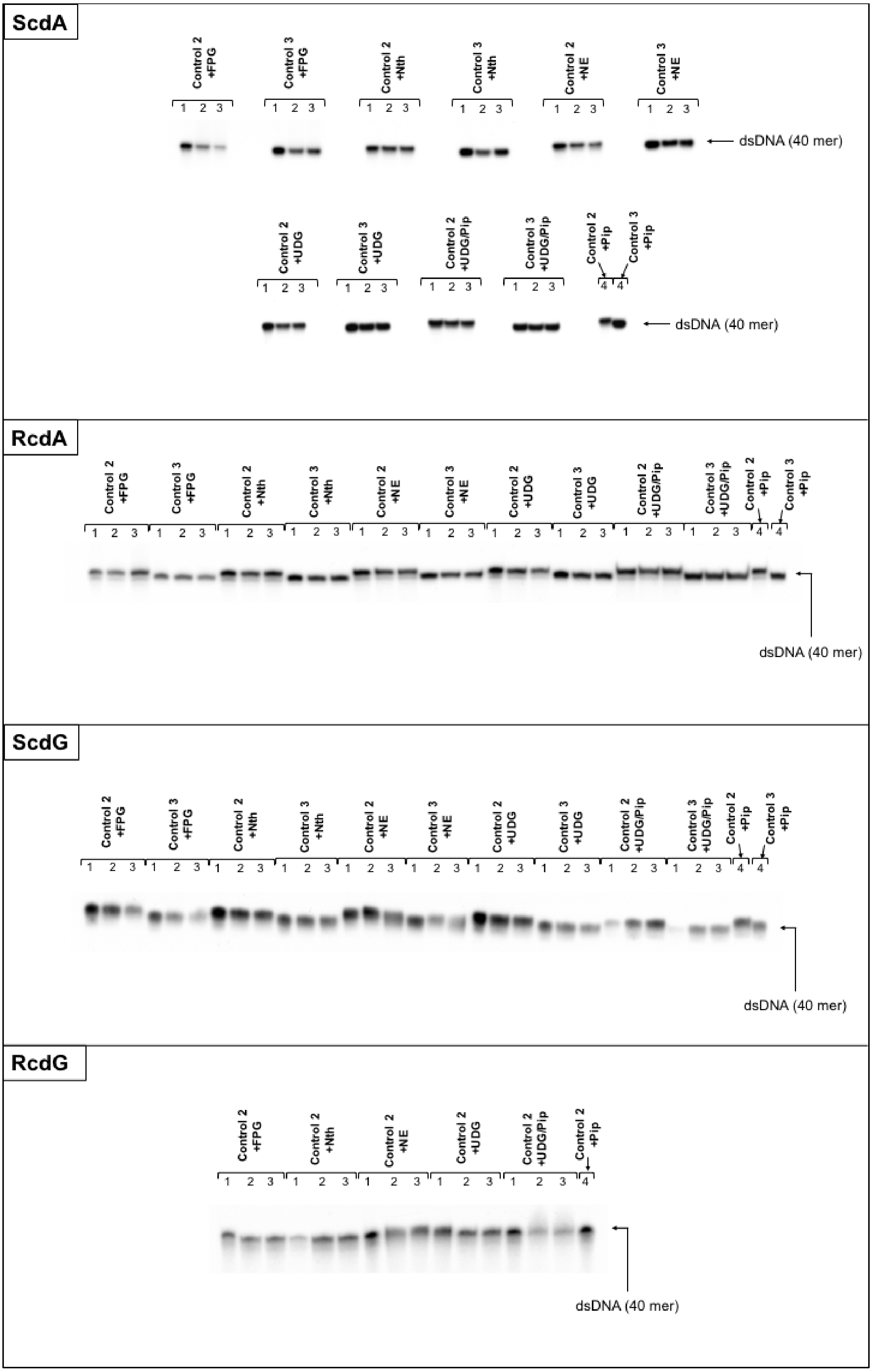

Figure S3. The stability of „matrix" oligonucleotides (Control 2 and Control 3) after treatment with FPG, Nth, NE, 1M piperidine, UDG, UDG with subsequent 1M piperidine. Each lane number corresponds with different assay time: lane 1 - 0 min; lane 2 - 1 min; lane 3 – 120 min, lane 4 – 30 min

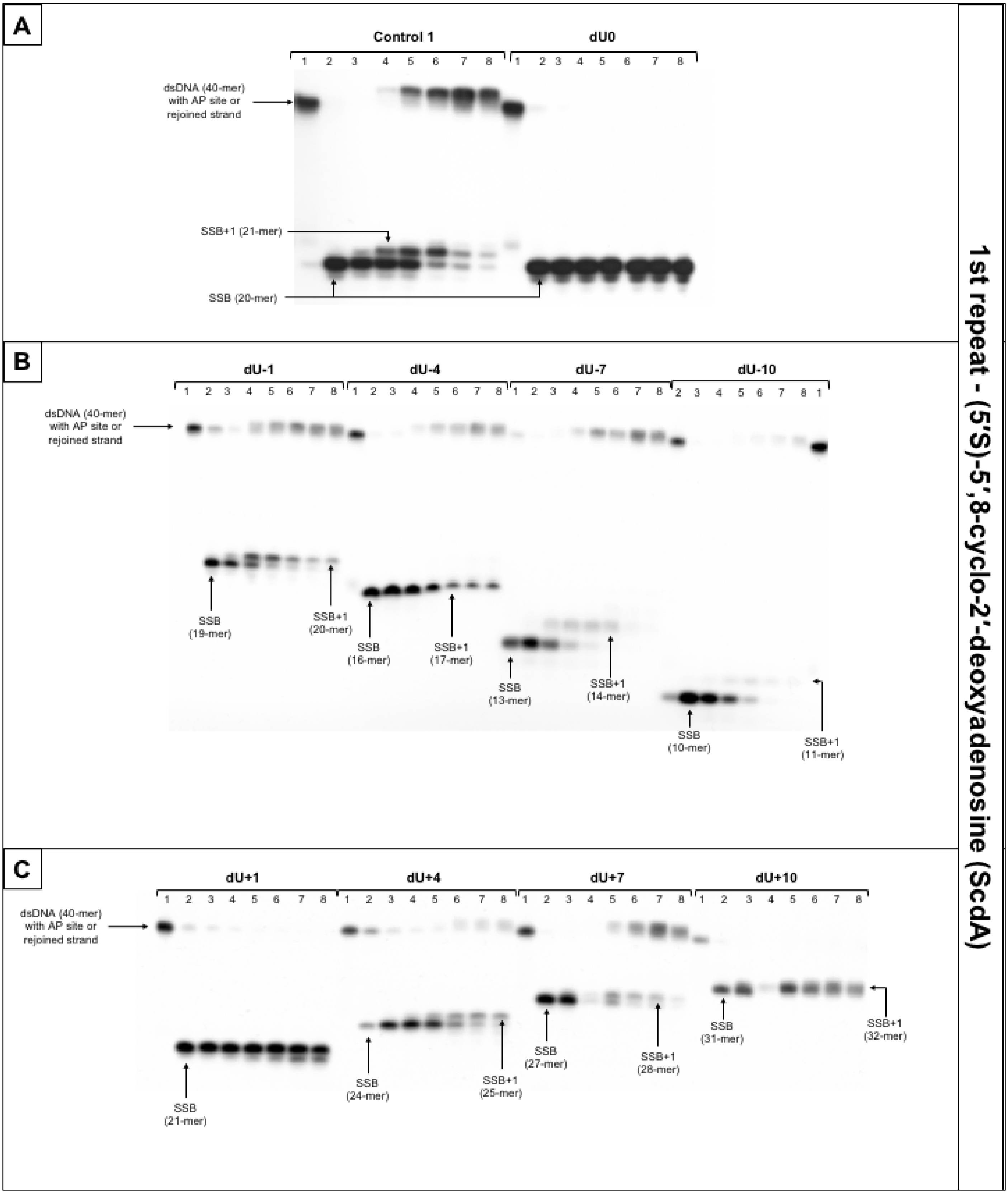
A
Control 1
dU0
dsDNA (40-mer) with AP site or rejoined strand
SSB+1 (21-mer)
SSB (20-mer)
B
dU-1
dU-4
dU-7
dU-10
dsDNA (40-mer) with AP site or rejoined strand
SSB (19-mer)
SSB+1 (20-mer)
SSB (16-mer)
SSB+1 (17-mer)
SSB (13-mer)
SSB+1 (14-mer)
SSB (10-mer)
SSB+1 (11-mer)
C
dU+1
dU+4
dU+7
dU+10
dsDNA (40-mer) with AP site or rejoined strand
SSB (21-mer)
SSB (24-mer)
SSB+1 (25-mer)
SSB (27-mer)
SSB+1 (28-mer)
SSB (31-mer)
SSB+1 (32-mer)
1st repeat - (5'S)-5',8-cyclo-2'-deoxyadenosine (ScdA)

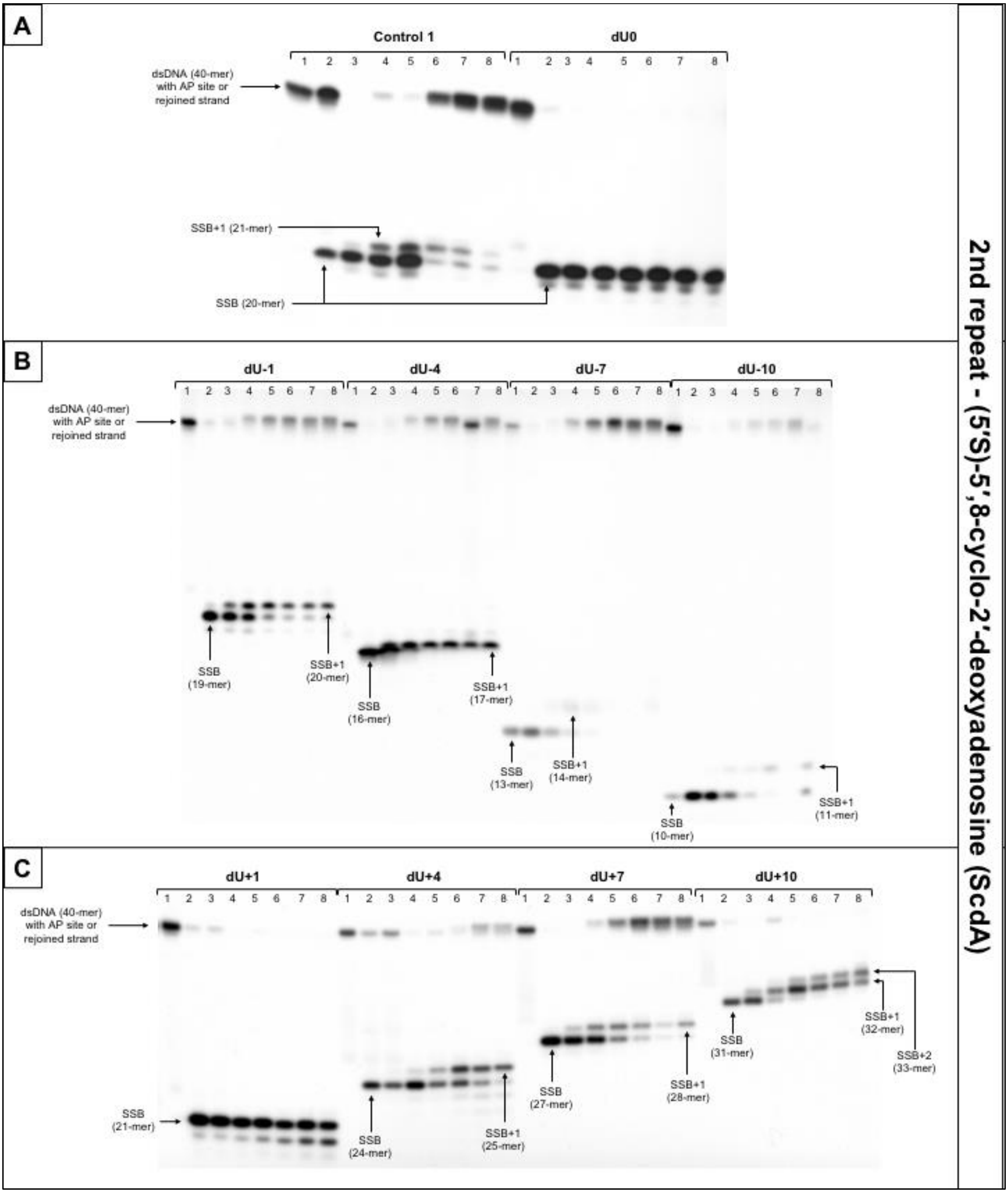
A
Control 1
dU0
dsDNA (40-mer) with AP site or rejoined strand
SSB+1 (21-mer)
SSB (20-mer)
B
dU-1
dU-4
dU-7
dU-10
SSB (19-mer)
SSB+1 (20-mer)
SSB (16-mer)
SSB+1 (17-mer)
SSB (13-mer)
SSB+1 (14-mer)
SSB (10-mer)
SSB+1 (11-mer)
C
dU+1
dU+4
dU+7
dU+10
SSB (21-mer)
SSB (24-mer)
SSB+1 (25-mer)
SSB (27-mer)
SSB+1 (28-mer)
SSB (31-mer)
SSB+1 (32-mer)
SSB+2 (33-mer)
2nd repeat - (5'S)-5',8-cyclo-2'-deoxyadenosine (ScdA)

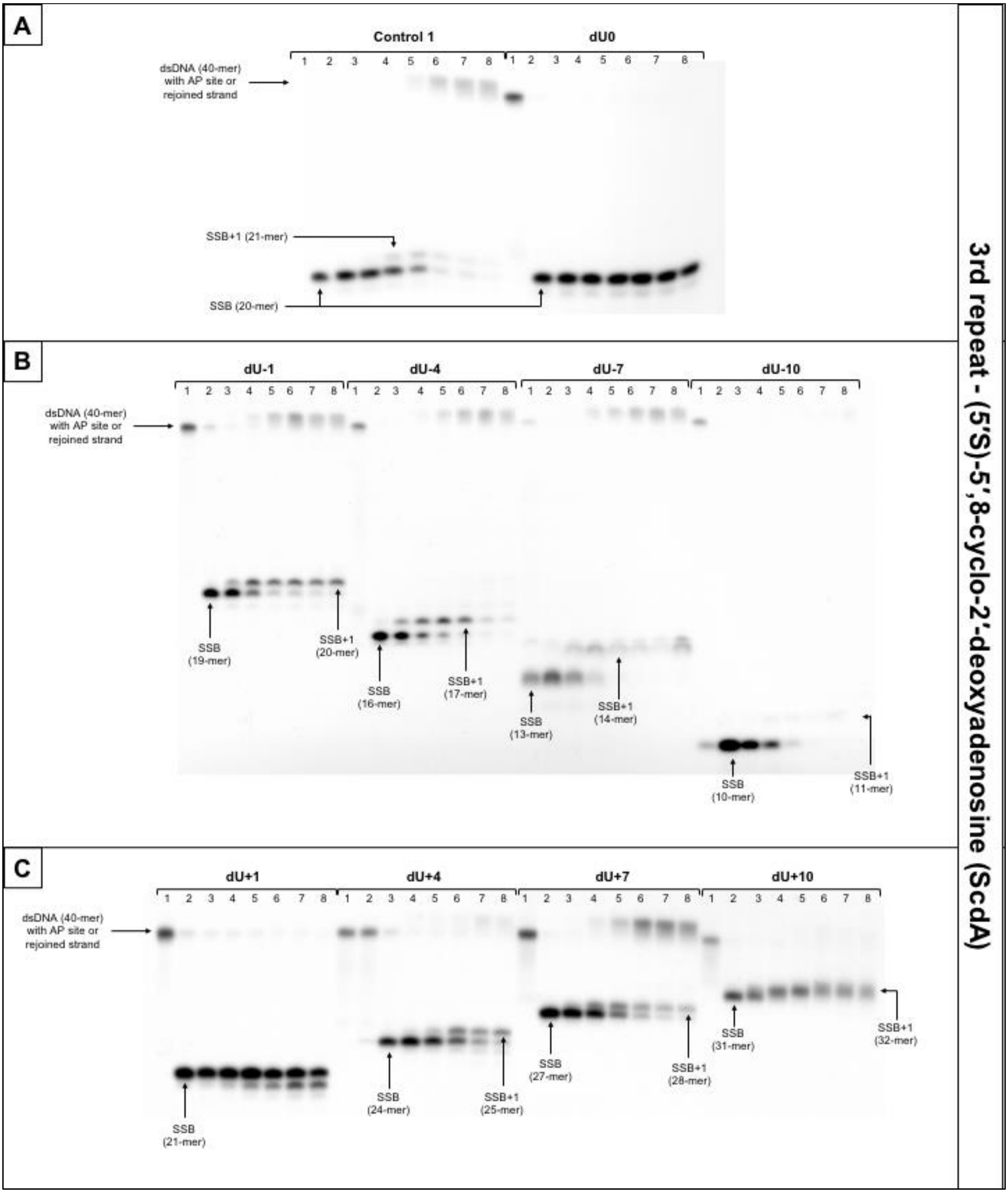

Figure S4. The autoradiograms of denaturing PAGE presenting repair of dsDNA containing clustered damage with AP site in one strand and ScdA in the opposing strand: (A) Controls: dsDNA with single lesion in one strand (Control 1); dsDNA with clustered lesions in two strands opposite to each other (dU0); (B) dsDNA with clustered lesions in two strands where AP site is located 1-10 base pairs in 3′ direction (negative numbers); (C) dsDNA with clustered lesions in two strands where AP site is located 1-10 base pairs in 5′ direction (positive numbers). Each lane corresponds with different assay time: lane 1 - 0 min; lane 2 - 1 min; lane 3 – 5 min; lane 4 – 15 min; lane 5 – 30 min; lane 6 – 60 min; lane 7 – 90 min; lane 8 – 120 min. Each replication of the experiment is shown.

A.

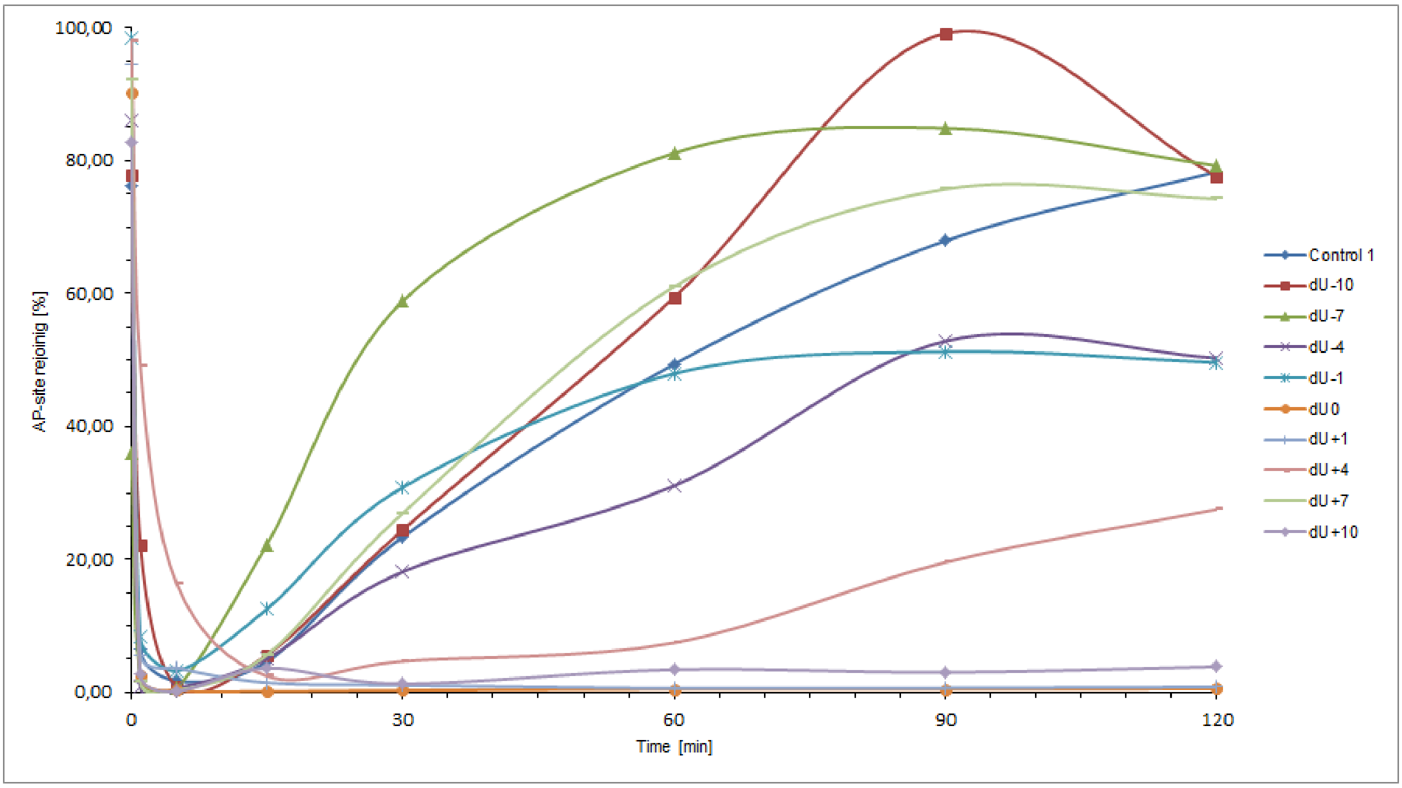
100,00
80,00
60,00
40,00
20,00
0,00
AP-site rejoinig [%]
0
30
60
90
120
Time [min]
Control 1
dU-10
dU-7
dU-4
dU-1
dU0
dU+1
dU+4
dU+7
dU+10

B.

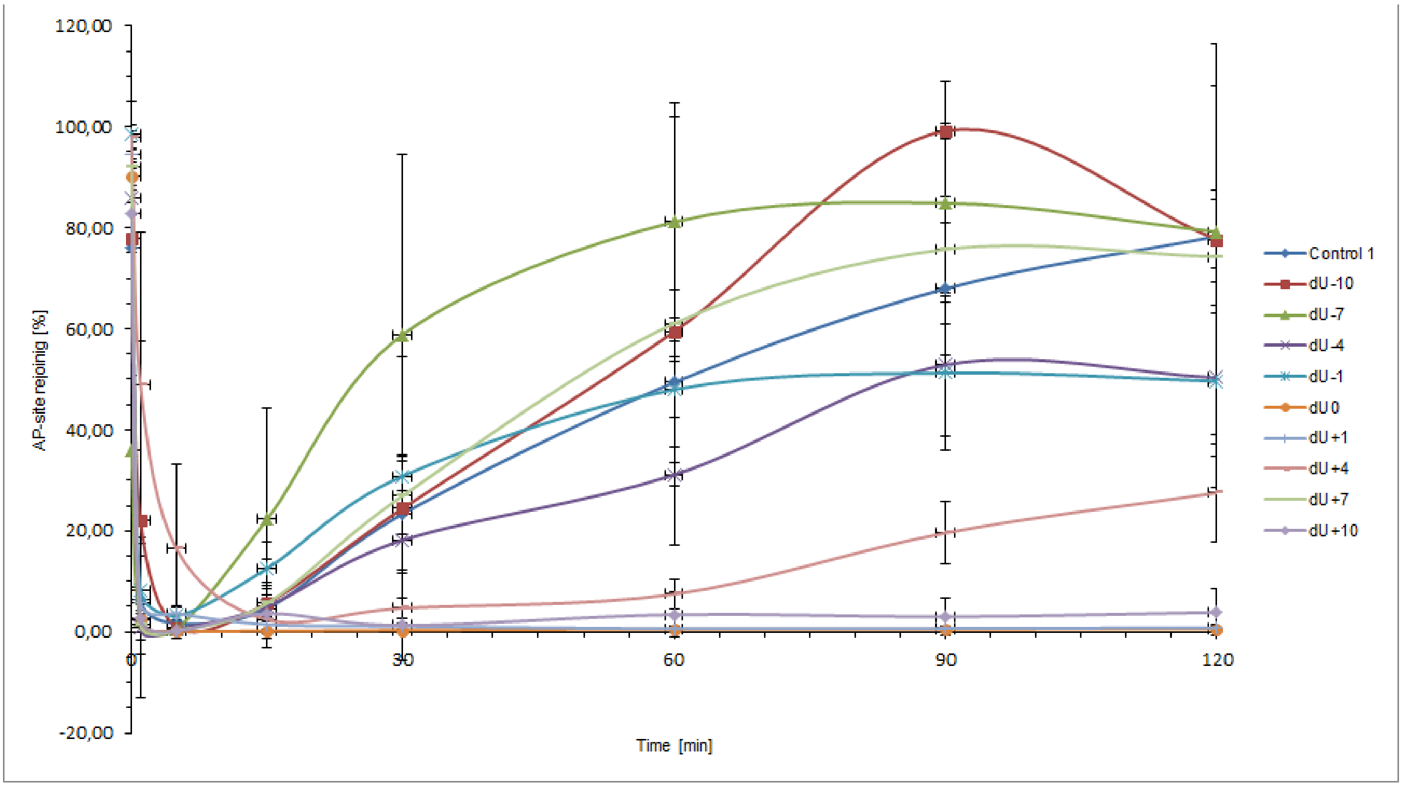
120,00
100,00
80,00
60,00
40,00
20,00
0,00
-20,00
AP-site rejoinig [%]
0
30
60
90
120
Time [min]
Control 1
dU-10
dU-7
dU-4
dU-1
dU0
dU+1
dU+4
dU+7
dU+10

C.

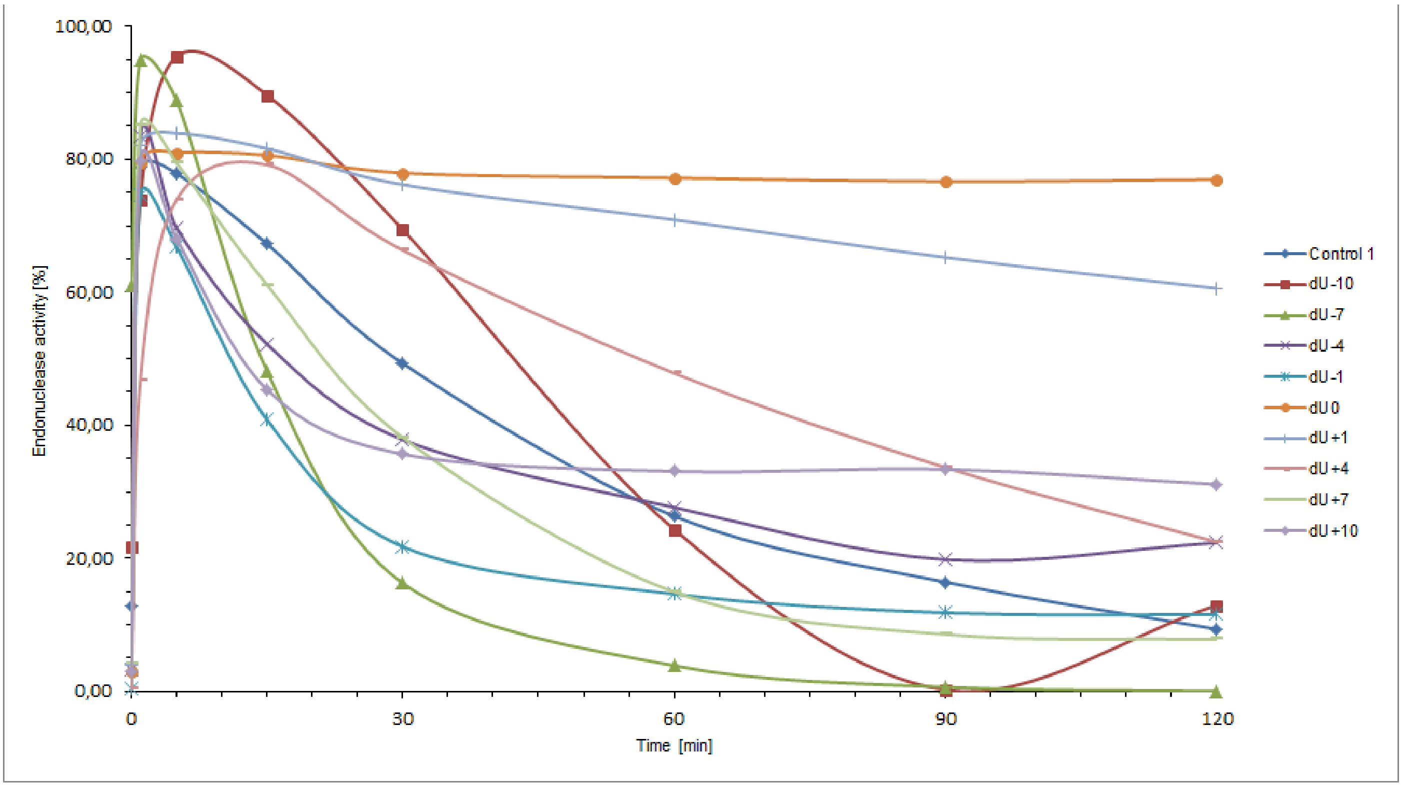
100,00
80,00
60,00
40,00
20,00
0,00
Endonuclease activity [%]
0
30
60
90
120
Time [min]
Control 1
dU-10
dU-7
dU-4
dU-1
dU0
dU+1
dU+4
dU+7
dU+10

D.

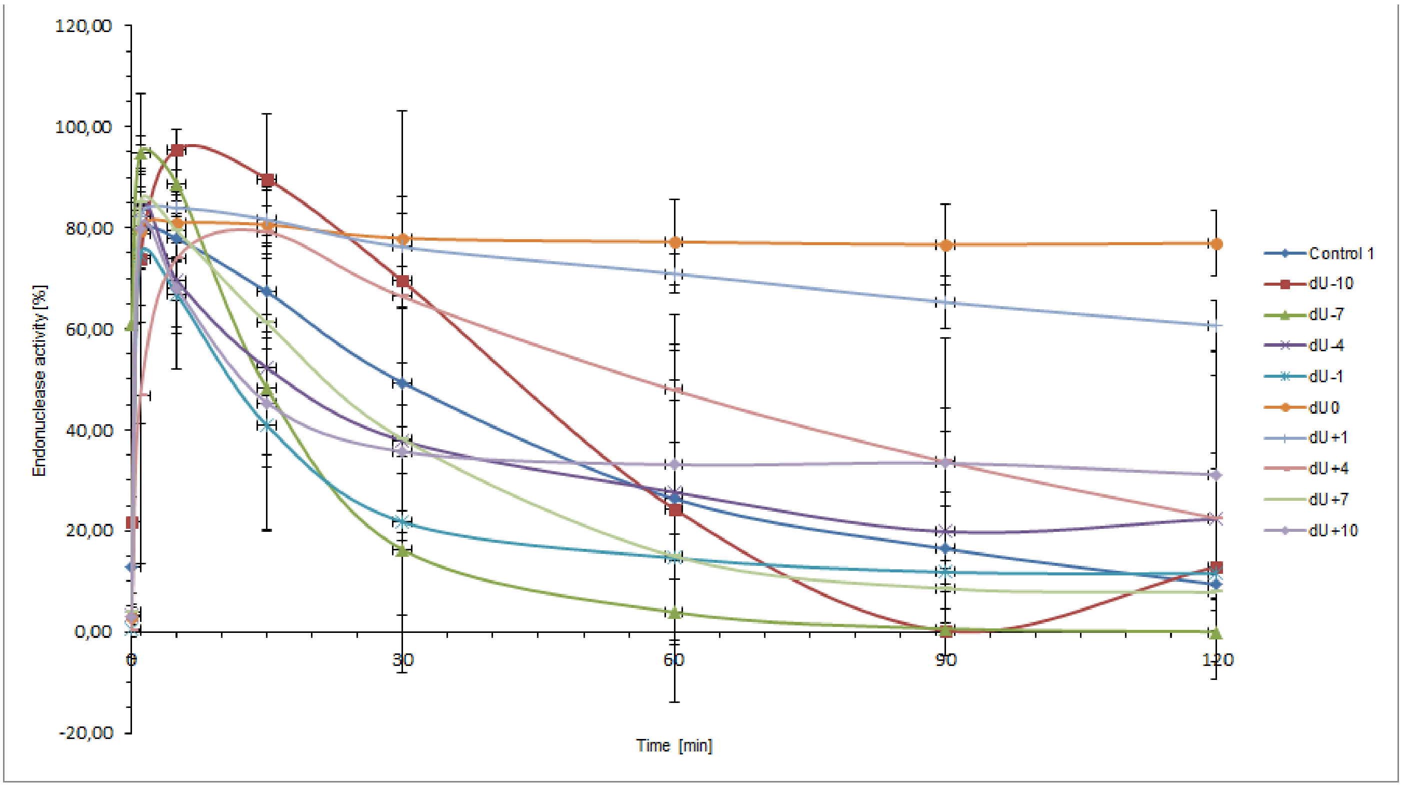
120,00
100,00
80,00
60,00
40,00
20,00
0,00
-20,00
Endonuclease activity [%]
0
30
60
90
120
Time [min]
Control 1
dU-10
dU-7
dU-4
dU-1
dU0
dU+1
dU+4
dU+7
dU+10

E.

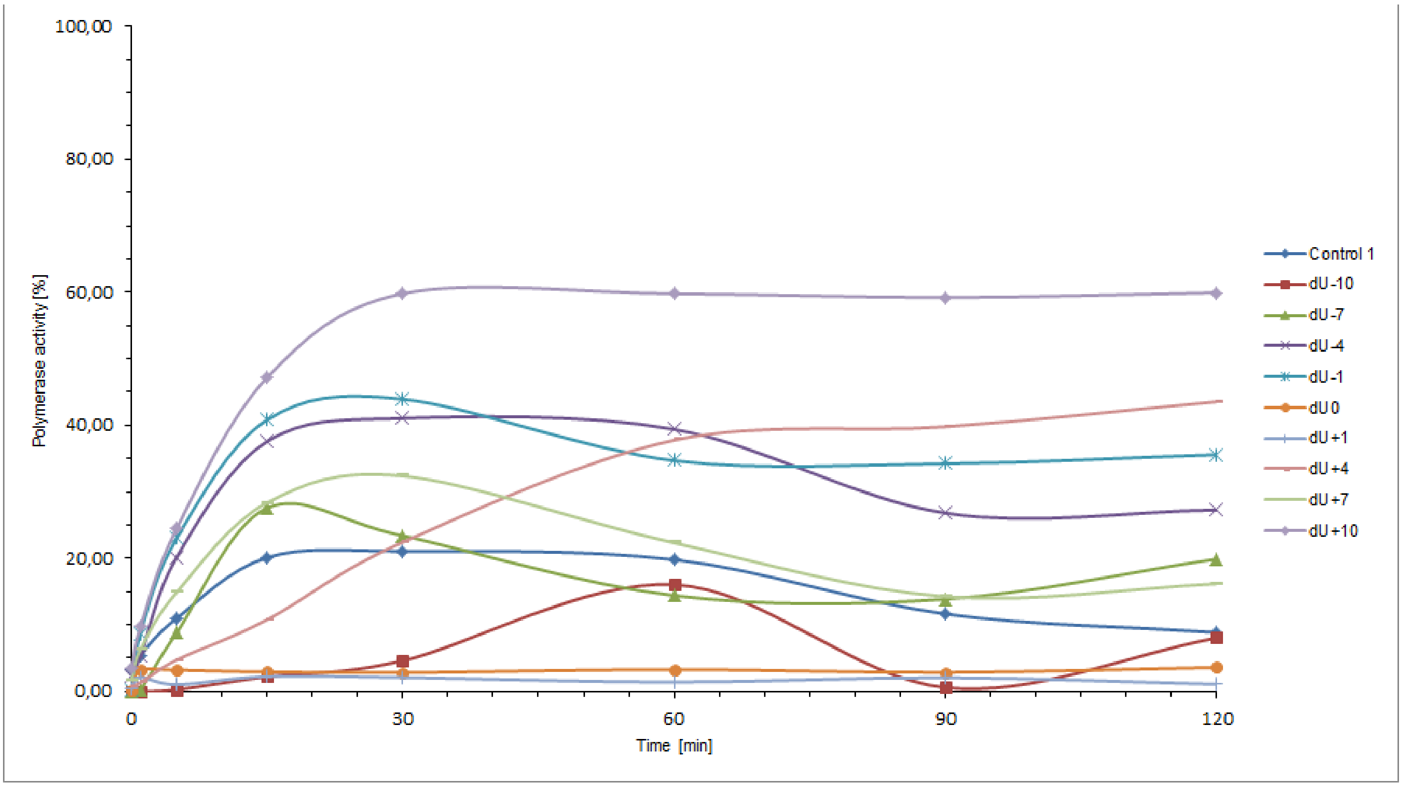

F.

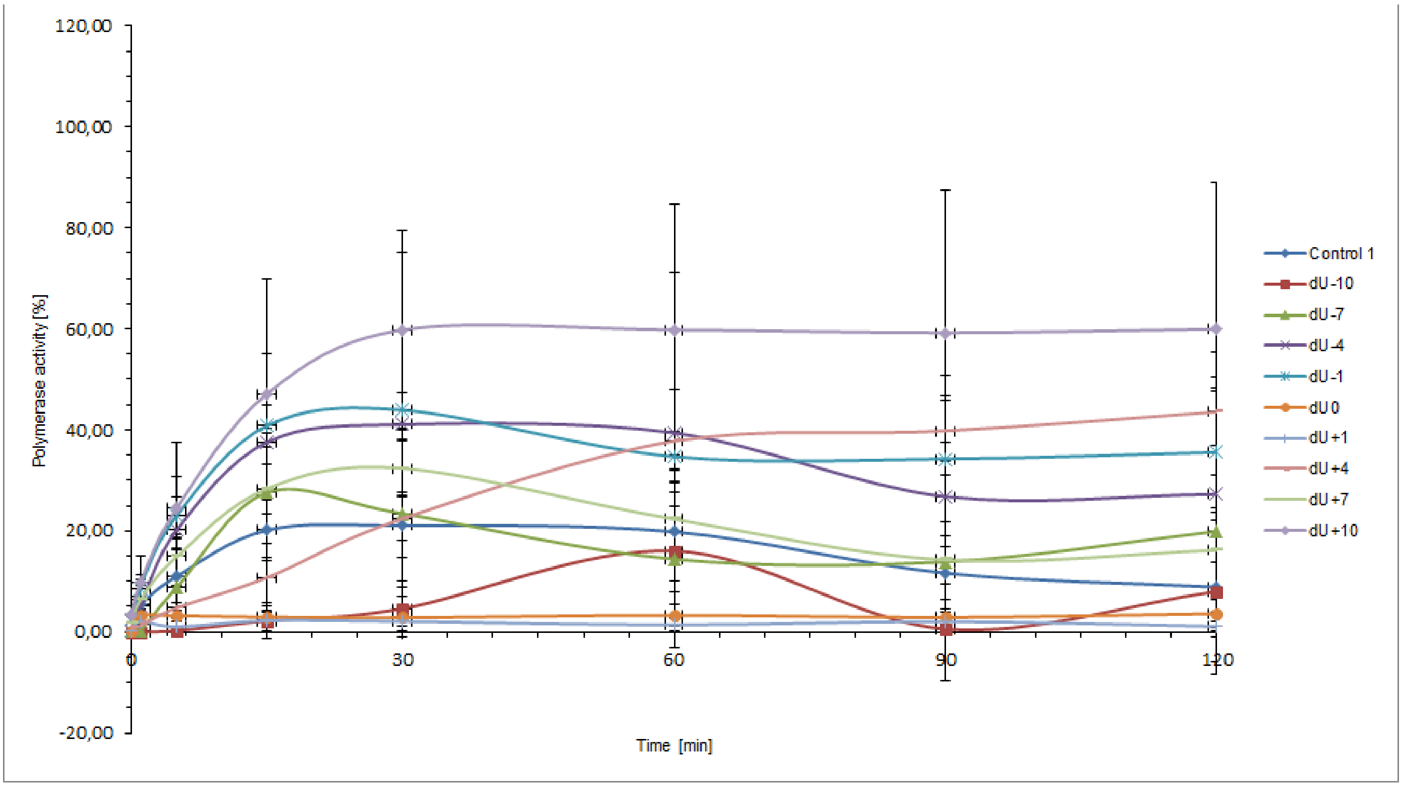

Figure S5. Graphical representation of DNA repair assays' results for ScdA. (A) AP site rejoining efficiency, (B) AP site rejoining efficiency + SD, (C) endonuclease activity, (D) endonuclease activity + SD, (E) polymerase activity, (F) polymerase activity + SD

Table S1. AP site rejoining - ScdA. Raw numerical data of densitometry obtained from Quantity One software.

ScdA		Time [min]							
		0	1	5	15	30	60	90	120
Strand	Data set	AP site rejoining [%]							
dU0	1.	89,18	3,70	0,30	0,07	0,09	0,05	0,05	0,07
	2.	93,45	2,32	0,27	0,07	0,21	0,45	0,51	0,43
	3.	88,30	1,55	0,43	0,38	0,66	1,01	0,96	1,19
	Avg	90,31	2,52	0,33	0,17	0,32	0,50	0,50	0,56
	SD	2,76	1,09	0,08	0,18	0,30	0,49	0,46	0,57
dU-1	1.	99,13	15,62	4,89	18,42	34,29	54,00	68,06	66,06
	2.	97,79	2,56	2,95	11,02	29,64	46,73	47,51	46,03
	3.	98,57	6,80	1,69	8,21	28,64	43,15	38,18	36,76
	Avg	98,49	8,33	3,18	12,55	30,86	47,96	51,25	49,62
	SD	0,67	6,66	1,61	5,28	3,02	5,53	15,28	14,97
dU-4	1.	89,48	1,75	1,42	4,91	17,74	33,60	46,41	42,81
	2.	85,98	0,00	0,00	7,30	24,30	29,19	43,04	33,20
	3.	82,60	0,63	0,49	3,13	12,46	30,47	69,14	74,88
	Avg	86,02	0,79	0,64	5,12	18,17	31,09	52,86	50,29
	SD	3,44	0,89	0,72	2,09	5,93	2,27	14,20	21,82
dU-7	1.	62,66	7,08	1,05	2,12	18,60	54,24	57,04	96,84
	2.	36,60	0,00	0,00	45,78	86,83	98,08	99,23	94,92
	3.	8,58	0,23	0,70	18,97	71,20	91,08	98,43	45,91
	Avg	35,95	2,43	0,58	22,29	58,88	81,13	84,90	79,23
	SD	27,05	4,02	0,53	22,02	35,74	23,55	24,13	28,87
dU-10	1.	100,00	62,86	0,62	0,34	2,00	15,50	100,00	100,00
	2.	86,13	3,49	2,20	15,76	58,46	62,75	97,47	32,97
	3.	47,62	0,27	0,05	0,93	12,90	100,00	100,00	100,00
	Avg	77,92	22,21	0,96	5,68	24,45	59,42	99,16	77,66
	SD	27,14	35,24	1,11	8,74	29,95	42,35	1,46	38,70
dU+1	1.	97,53	6,01	2,84	1,43	0,72	0,53	0,46	0,38
	2.	93,05	5,74	5,39	0,45	0,79	0,16	0,31	0,71
	3.	92,67	5,31	2,88	2,55	1,75	1,20	1,25	1,36
	Avg	94,42	5,69	3,70	1,48	1,09	0,63	0,68	0,82
	SD	2,70	0,35	1,46	1,05	0,58	0,53	0,50	0,50
dU+4	1.	98,59	44,18	5,17	1,93	3,06	10,78	16,89	28,04
	2.	100,00	21,54	35,56	2,58	6,77	5,42	26,54	36,97
	3.	95,31	81,33	8,44	2,87	4,24	6,20	15,36	17,60
	Avg	97,97	49,01	16,39	2,46	4,69	7,47	19,59	27,53
	SD	2,41	30,19	16,68	0,48	1,90	2,90	6,06	9,69
dU+7	1.	90,12	1,56	0,22	2,05	21,60	59,48	75,38	86,49
	2.	96,34	1,10	0,41	8,62	35,87	68,20	86,22	72,13
	3.	89,92	1,94	0,29	6,34	23,44	55,44	65,55	64,17
	Avg	92,13	1,53	0,31	5,67	26,97	61,04	75,72	74,26
	SD	3,65	0,42	0,10	3,34	7,76	6,52	10,33	11,31
dU+10	1.	91,35	2,67	0,16	0,50	0,57	1,10	1,53	1,69
	2.	79,91	4,31	0,36	9,20	0,42	0,59	0,37	0,64
	3.	76,83	1,12	0,00	1,19	2,92	8,60	7,12	9,29
	Avg	82,70	2,70	0,17	3,63	1,30	3,43	3,00	3,87
	SD	7,65	1,59	0,18	4,84	1,40	4,48	3,61	4,72

Table S2. Endonuclease activity - ScdA. Raw numerical data of densitometry obtained from Quantity One software.

ScdA		Time [min]							
		0	1	5	15	30	60	90	120
Strand	Data set	Endonuclease activity [%]							
dU0	1.	3,20	76,09	78,83	78,50	75,49	76,43	75,21	75,41
	2.	2,52	74,20	75,37	75,16	71,10	69,17	69,60	71,53
	3.	3,52	88,33	88,77	88,14	87,27	85,97	85,18	83,91
	Avg	3,08	79,54	80,99	80,60	77,95	77,19	76,66	76,95
	SD	0,51	7,67	6,96	6,74	8,36	8,43	7,89	6,33
dU-1	1.	0,50	63,43	63,11	34,08	20,68	13,99	9,02	8,93
	2.	0,33	77,46	62,92	45,24	24,24	14,81	13,19	12,24
	3.	0,63	82,62	74,38	43,23	20,36	15,10	13,21	13,48
	Avg	0,48	74,50	66,80	40,85	21,76	14,63	11,81	11,55
	SD	0,15	9,93	6,57	5,95	2,16	0,57	2,41	2,35
dU-4	1.	3,16	89,86	88,44	87,81	77,07	61,36	48,16	55,05
	2.	4,35	75,58	53,63	25,75	12,64	7,86	4,58	8,82
	3.	2,43	84,69	66,92	43,10	23,82	13,79	6,72	3,22
	Avg	3,31	83,37	69,67	52,22	37,84	27,67	19,82	22,36
	SD	0,97	7,23	17,57	32,02	34,43	29,33	24,57	28,44
dU-7	1.	35,36	91,54	95,61	79,72	44,33	11,29	1,90	0,00
	2.	62,64	97,86	84,51	25,94	4,45	0,33	0,05	0,11
	3.	85,01	95,40	86,30	38,74	0,00	0,00	0,00	0,00
	Avg	61,00	94,93	88,81	48,13	16,26	3,87	0,65	0,04
	SD	24,87	3,18	5,96	28,09	24,41	6,43	1,08	0,06
dU-10	1.	0,00	36,97	94,13	94,70	90,79	68,60	0,00	0,00
	2.	13,08	85,67	92,30	75,16	30,36	4,47	0,67	38,81
	3.	52,38	99,16	99,95	99,07	87,10	0,00	0,00	0,00
	Avg	21,82	73,94	95,46	89,64	69,42	24,35	0,22	12,94
	SD	27,26	32,71	3,99	12,74	33,87	38,38	0,39	22,41
dU+1	1.	2,15	82,12	84,37	81,00	76,32	70,51	67,87	64,88
	2.	4,68	84,69	86,15	88,62	82,79	74,95	68,78	61,80
	3.	5,07	79,41	81,15	75,25	69,45	67,28	59,10	55,13
	Avg	3,97	82,07	83,89	81,62	76,19	70,91	65,25	60,60
	SD	1,59	2,64	2,53	6,71	6,67	3,85	5,35	4,99
dU+4	1.	0,68	50,19	80,07	72,13	64,34	48,10	33,92	24,68
	2.	0,00	78,46	64,44	88,96	66,37	45,60	27,56	11,83
	3.	0,31	11,90	77,20	76,38	68,28	49,82	39,49	30,85
	Avg	0,33	46,85	73,90	79,16	66,33	47,84	33,65	22,45
	SD	0,34	33,41	8,32	8,75	1,97	2,12	5,97	9,70
dU+7	1.	8,07	90,58	85,83	60,63	39,12	14,30	9,67	6,03
	2.	1,80	86,14	77,77	59,78	31,05	10,82	4,03	5,49
	3.	2,32	78,42	74,72	62,90	44,32	19,72	11,98	11,95
	Avg	4,06	85,05	79,44	61,10	38,16	14,95	8,56	7,83
	SD	3,48	6,15	5,74	1,62	6,69	4,48	4,09	3,58
dU+10	1.	4,75	86,88	75,36	58,15	50,80	56,39	57,63	53,66
	2.	1,34	81,59	70,83	32,87	16,32	11,11	8,29	5,21
	3.	3,25	70,83	57,98	45,05	39,89	31,73	34,22	34,49
	Avg	3,11	79,77	68,06	45,35	35,67	33,08	33,38	31,12
	SD	1,71	8,18	9,02	12,64	17,62	22,67	24,68	24,40

Table S3. Polymerase activity - ScdA. Raw numerical data of densitometry obtained from Quantity One software.

ScdA		Time [min]							
		0	1	5	15	30	60	90	120
Strand	Data set	Polymerase activity [%]							
dU0	1.	0,34	2,61	3,85	2,85	1,77	2,43	2,33	2,91
	2.	0,34	5,26	3,85	4,06	4,98	5,83	4,59	6,71
	3.	0,00	1,71	1,94	1,95	1,78	1,64	1,73	1,22
	Avg	0,23	3,20	3,21	2,95	2,84	3,30	2,88	3,61
	SD	0,19	1,84	1,10	1,06	1,85	2,23	1,51	2,81
dU-1	1.	0,19	9,93	24,26	40,64	41,05	29,08	20,54	22,41
	2.	0,01	10,36	25,78	36,80	42,85	36,14	36,82	38,29
	3.	0,05	5,73	18,97	45,00	47,89	38,88	45,38	46,04
	Avg	0,08	8,67	23,01	40,81	43,93	34,70	34,25	35,58
	SD	0,09	2,56	3,57	4,10	3,55	5,06	12,62	12,05
dU-4	1.	0,49	0,00	1,77	1,67	1,87	3,50	4,52	2,14
	2.	3,40	10,11	35,90	64,54	62,15	62,57	52,01	57,80
	3.	3,08	5,64	22,81	46,51	59,21	52,16	23,88	21,90
	Avg	2,33	5,25	20,16	37,57	41,08	39,41	26,80	27,28
	SD	1,60	5,07	17,22	32,37	33,99	31,53	23,88	28,22
dU-7	1.	0,00	0,00	0,00	15,06	37,06	34,47	41,06	3,16
	2.	0,18	0,74	14,04	27,00	8,09	1,43	0,54	3,87
	3.	0,00	0,00	12,34	40,64	25,06	7,29	0,00	52,77
	Avg	0,06	0,25	8,80	27,57	23,41	14,40	13,87	19,93
	SD	0,10	0,43	7,66	12,80	14,56	17,63	23,55	28,44
dU-10	1.	0,00	0,00	0,00	0,22	3,01	15,90	0,00	0,00
	2.	0,00	0,00	0,81	6,40	10,85	32,27	1,73	24,14
	3.	0,00	0,00	0,00	0,00	0,00	0,00	0,00	0,00
	Avg	0,00	0,00	0,27	2,21	4,62	16,06	0,58	8,05
	SD	0,00	0,00	0,47	3,63	5,60	16,14	1,00	13,94
dU+1	1.	0,00	0,00	0,00	3,36	2,69	1,84	3,78	2,01
	2.	0,00	1,53	0,00	0,00	0,00	0,00	0,00	0,00
	3.	1,00	4,72	3,08	3,33	3,54	2,35	2,35	1,22
	Avg	0,33	2,08	1,03	2,23	2,08	1,40	2,04	1,08
	SD	0,57	2,41	1,78	1,93	1,85	1,24	1,91	1,01
dU+4	1.	0,41	0,54	5,95	16,46	22,30	29,38	39,59	38,06
	2.	0,00	0,00	0,00	3,18	26,86	48,98	45,90	51,20
	3.	1,83	3,36	8,25	12,68	18,17	34,97	33,88	41,43
	Avg	0,74	1,30	4,74	10,78	22,44	37,78	39,79	43,56
	SD	0,96	1,81	4,26	6,84	4,35	10,10	6,02	6,82
dU+7	1.	0,93	4,25	10,76	34,04	38,45	25,28	14,30	6,52
	2.	0,00	6,13	15,50	26,10	31,06	20,26	9,38	21,84
	3.	4,17	8,50	18,38	24,94	27,65	21,48	19,05	20,18
	Avg	1,70	6,29	14,88	28,36	32,39	22,34	14,24	16,18
	SD	2,19	2,13	3,85	4,95	5,52	2,62	4,84	8,41
dU+10	1.	1,57	6,92	21,99	39,83	47,70	41,03	37,99	43,09
	2.	1,11	6,34	19,88	55,49	82,58	87,87	91,14	93,48
	3.	7,57	15,80	31,58	46,19	49,13	50,49	48,54	43,27
	Avg	3,42	9,68	24,48	47,17	59,80	59,79	59,22	59,95
	SD	3,61	5,30	6,24	7,88	19,74	24,77	28,14	29,04

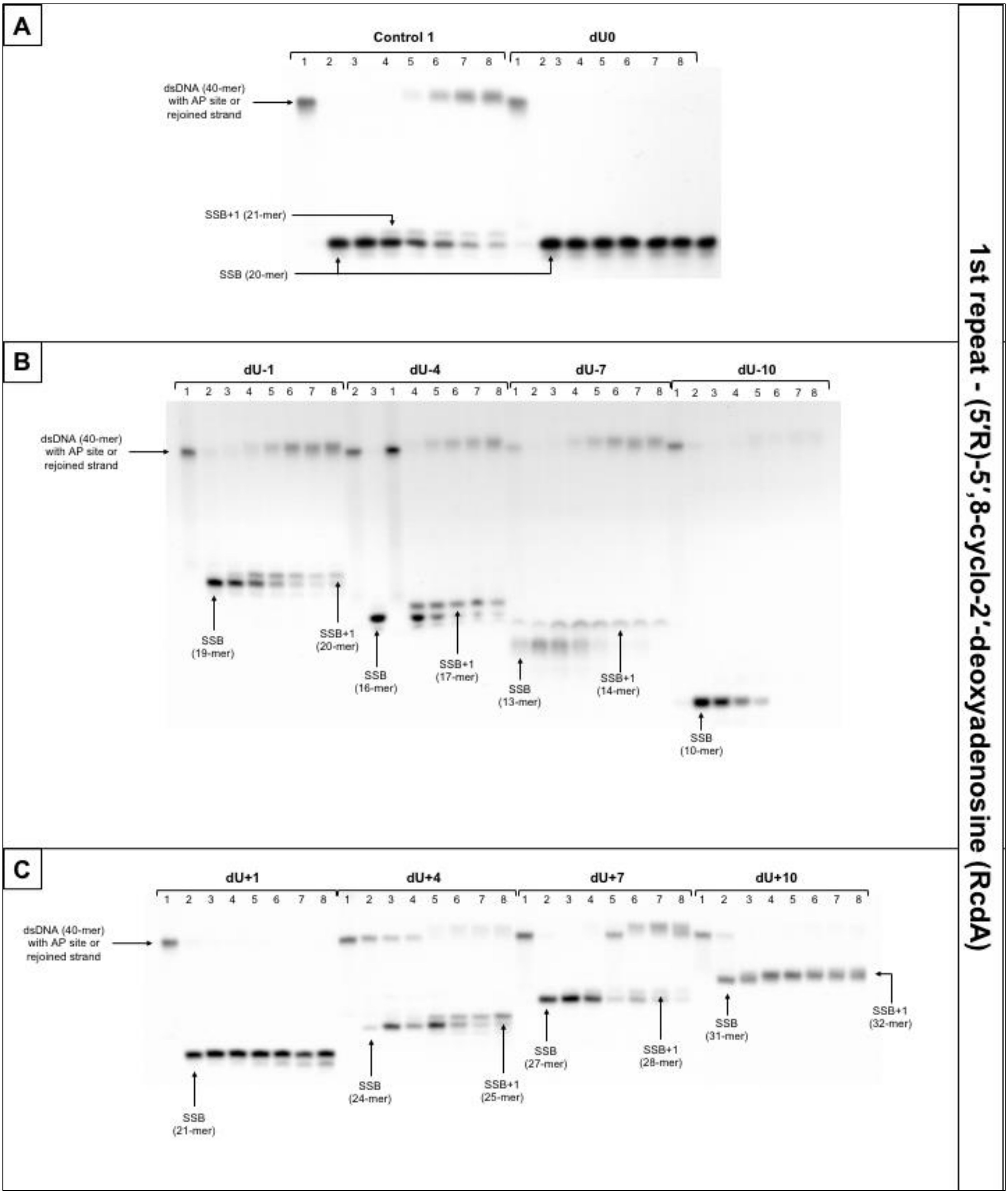
A
Control 1
dU0
1 2 3 4 5 6 7 8
1 2 3 4 5 6 7 8
dsDNA (40-mer) with AP site or rejoined strand
SSB+1 (21-mer)
SSB (20-mer)
B
dU-1
dU-4
dU-7
dU-10
SSB (19-mer)
SSB+1 (20-mer)
SSB (16-mer)
SSB+1 (17-mer)
SSB (13-mer)
SSB+1 (14-mer)
SSB (10-mer)
C
dU+1
dU+4
dU+7
dU+10
SSB (21-mer)
SSB (24-mer)
SSB+1 (25-mer)
SSB (27-mer)
SSB+1 (28-mer)
SSB (31-mer)
SSB+1 (32-mer)
1st repeat - (5'R)-5',8-cyclo-2'-deoxyadenosine (RcdA)

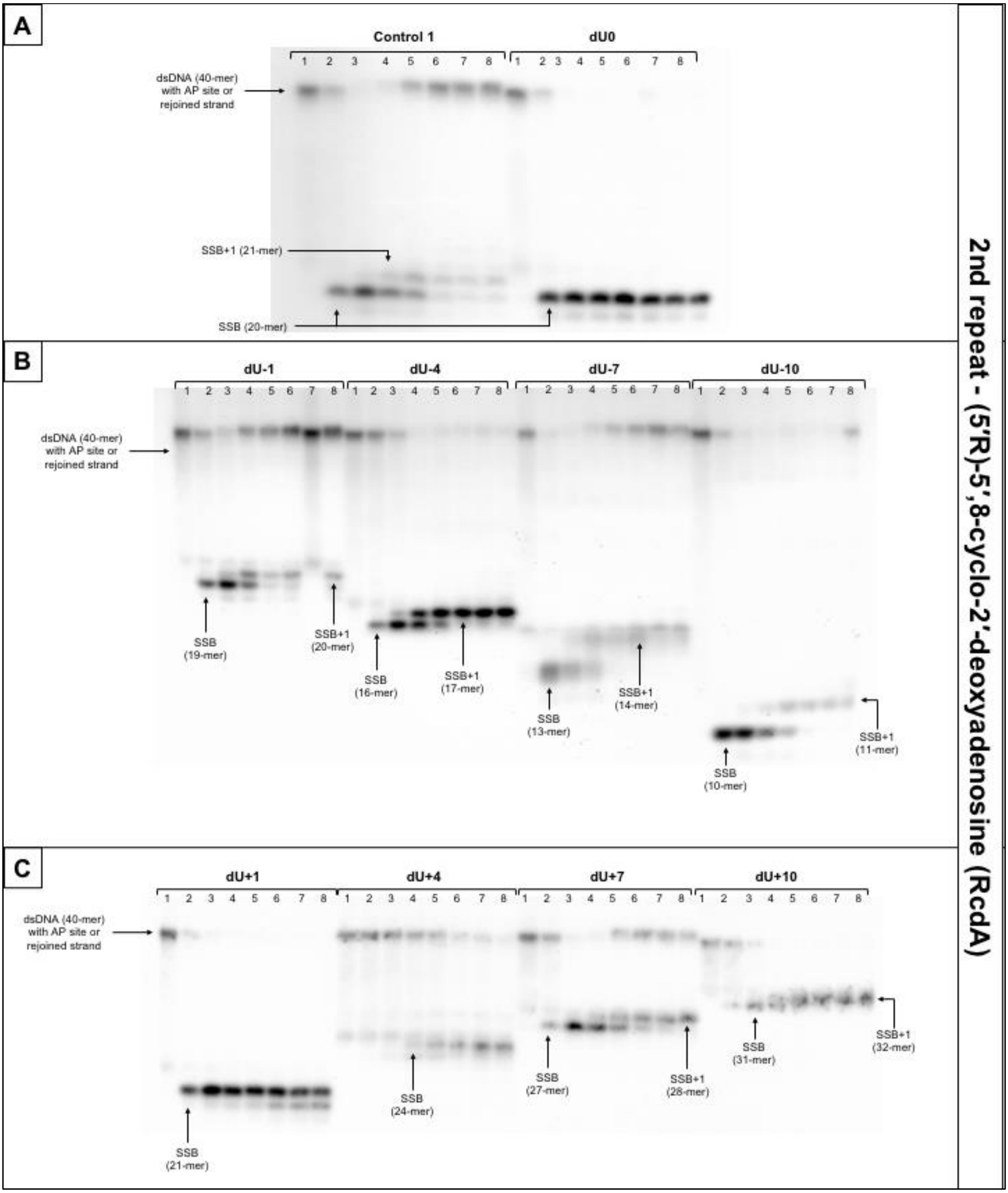
A
Control 1
dU0
1 2 3 4 5 6 7 8
dsDNA (40-mer) with AP site or rejoined strand
SSB+1 (21-mer)
SSB (20-mer)
B
dU-1
dU-4
dU-7
dU-10
SSB (19-mer)
SSB+1 (20-mer)
SSB (16-mer)
SSB+1 (17-mer)
SSB (13-mer)
SSB+1 (14-mer)
SSB (10-mer)
SSB+1 (11-mer)
C
dU+1
dU+4
dU+7
dU+10
SSB (21-mer)
SSB (24-mer)
SSB (27-mer)
SSB+1 (28-mer)
SSB (31-mer)
SSB+1 (32-mer)
2nd repeat - (5'R)-5',8-cyclo-2'-deoxyadenosine (RcdA)

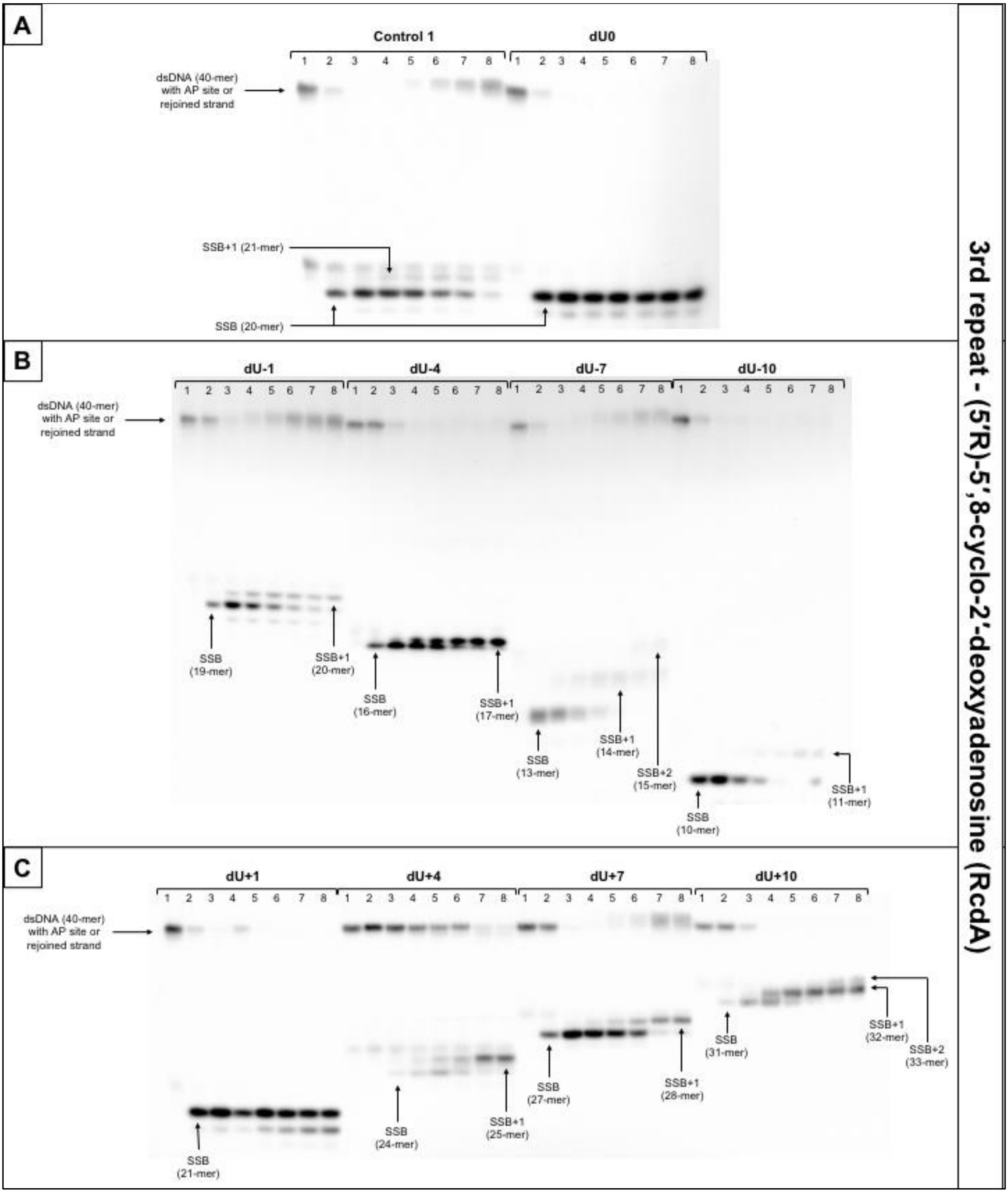

Figure S6. The autoradiograms of denaturing PAGE presenting repair of dsDNA containing clustered damage with AP site in one strand and RcdA in the opposing strand: (A) Controls: dsDNA with single lesion in one strand (Control 1); dsDNA with clustered lesions in two strands opposite to each other (dU0); (B) dsDNA with clustered lesions in two strands where AP site is located 1-10 base pairs in 3′ direction (negative numbers); (C) dsDNA with clustered lesions in two strands where AP site is located 1-10 base pairs in 5′ direction (positive numbers). Each lane corresponds with different assay time: lane 1 - 0 min; lane 2 - 1 min; lane 3 – 5 min; lane 4 – 15 min; lane 5 – 30 min; lane 6 – 60 min; lane 7 – 90 min; lane 8 – 120 min. Each replication of the experiment is shown.

A.

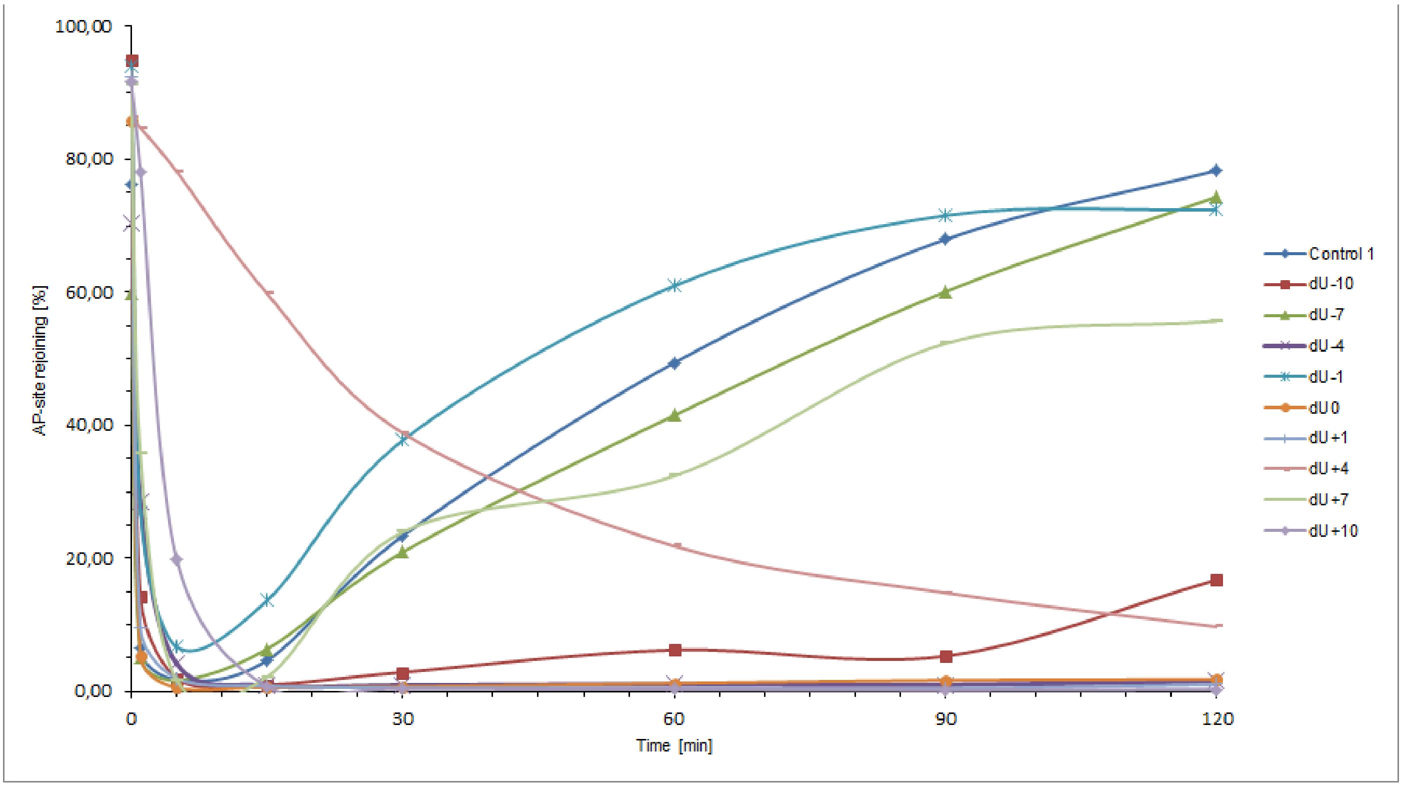
100,00
80,00
60,00
40,00
20,00
0,00
AP-site rejoining [%]
0
30
60
90
120
Time [min]
Control 1
dU -10
dU -7
dU -4
dU -1
dU 0
dU +1
dU +4
dU +7
dU +10

B.

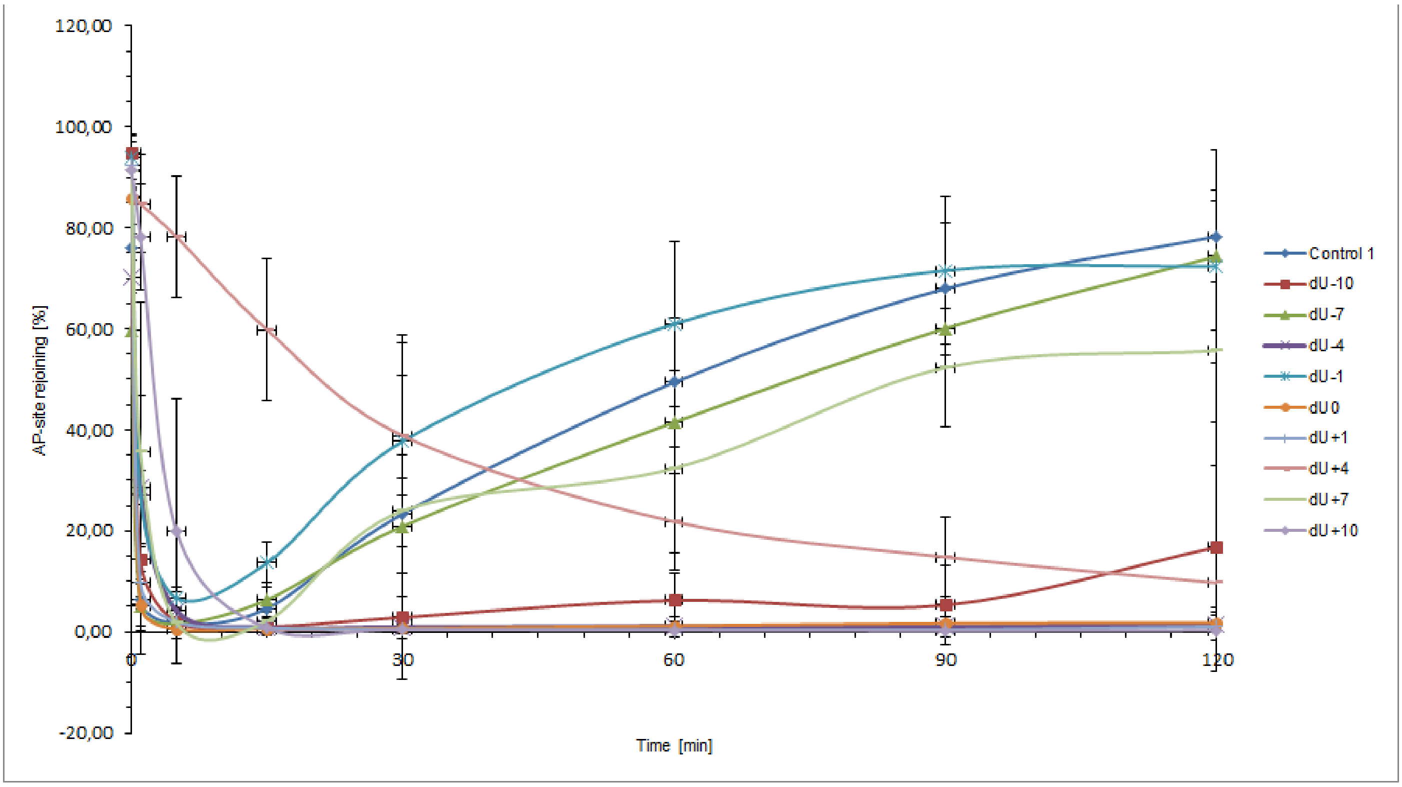
120,00
100,00
80,00
60,00
40,00
20,00
0,00
-20,00
AP-site rejoining [%]
0
30
60
90
120
Time [min]
Control 1
dU -10
dU -7
dU -4
dU -1
dU 0
dU +1
dU +4
dU +7
dU +10

C.

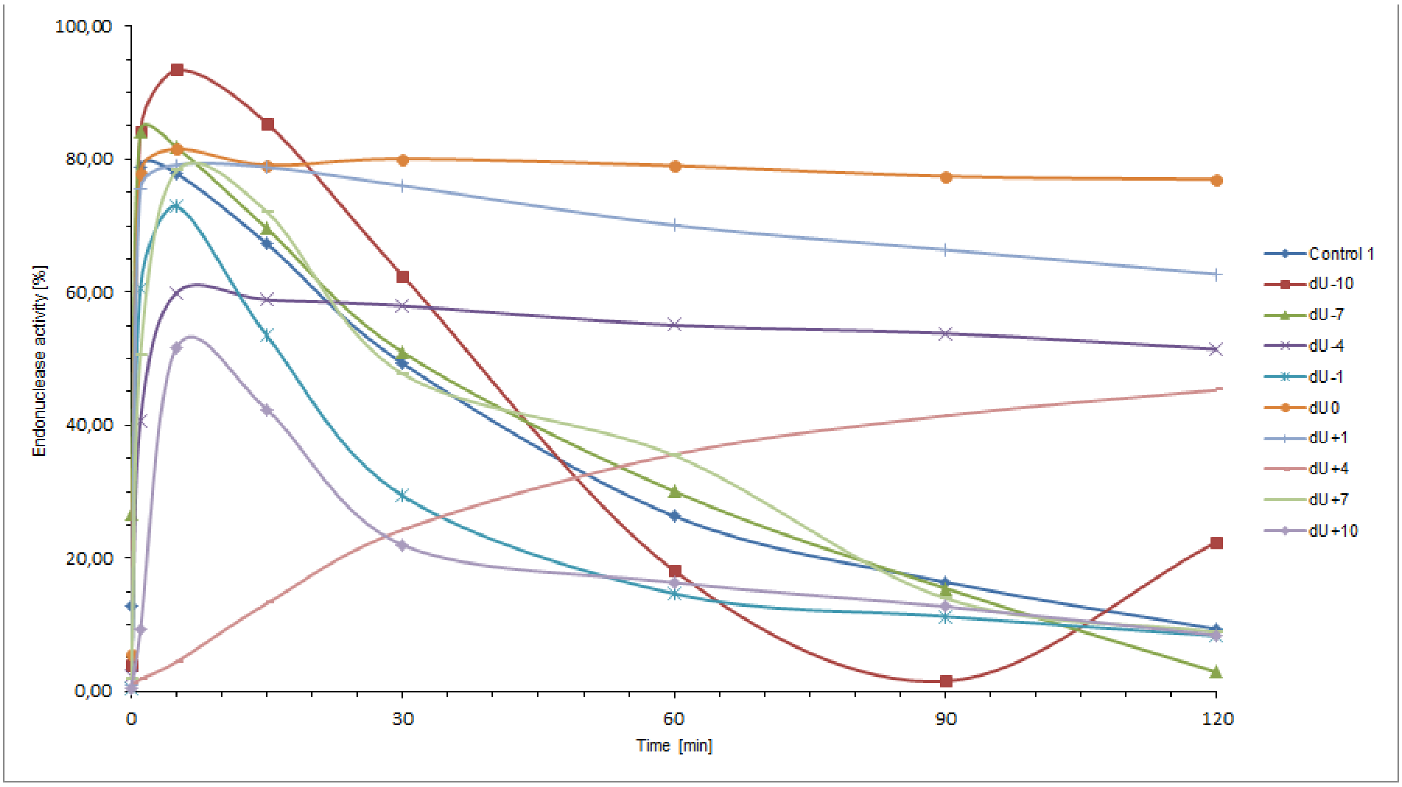
100,00
80,00
60,00
40,00
20,00
0,00
Endonuclease activity [%]
0
30
60
90
120
Time [min]
Control 1
dU-10
dU-7
dU-4
dU-1
dU0
dU+1
dU+4
dU+7
dU+10

D.

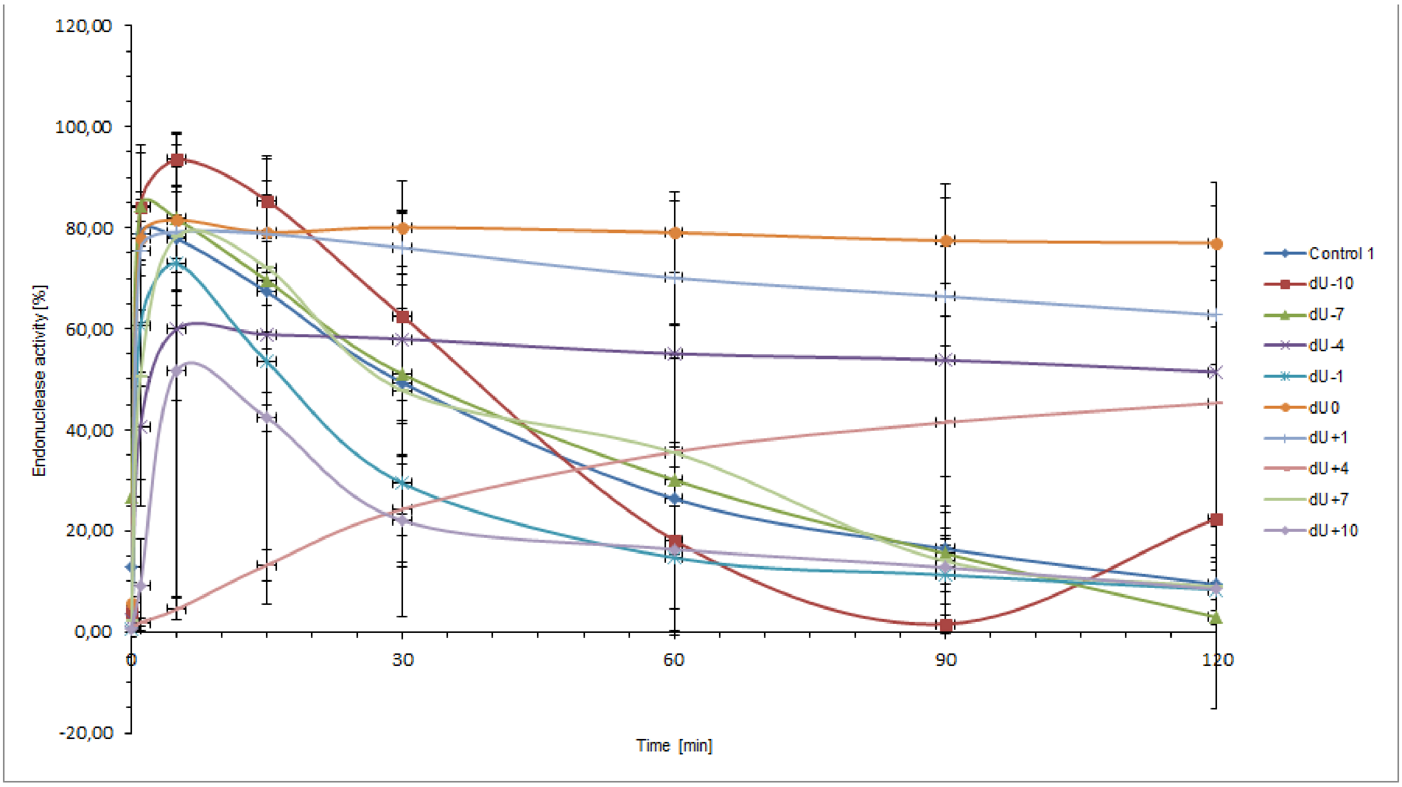
120,00
100,00
80,00
60,00
40,00
20,00
0,00
-20,00
Endonuclease activity [%]
0
30
60
90
120
Time [min]
Control 1
dU-10
dU-7
dU-4
dU-1
dU0
dU+1
dU+4
dU+7
dU+10

E.

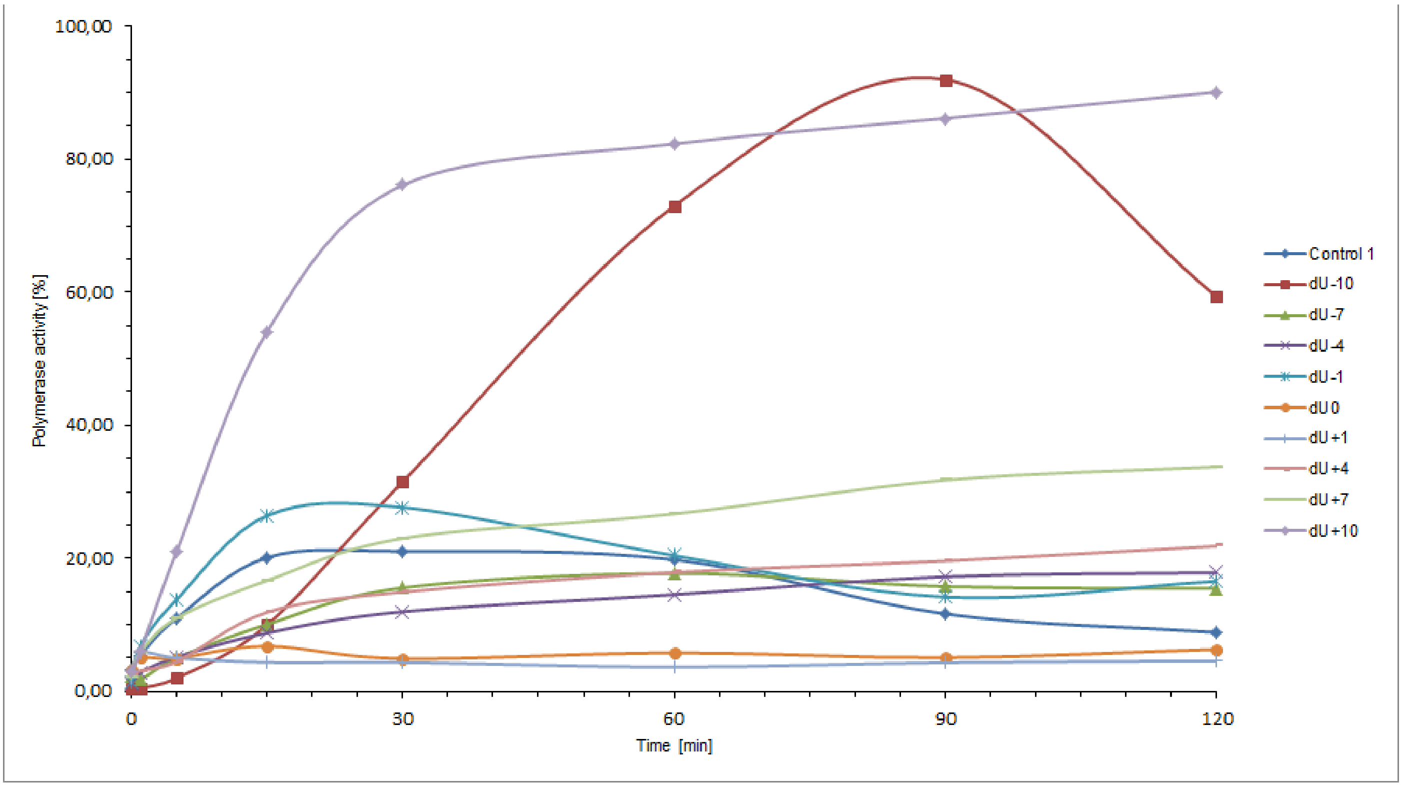

F.

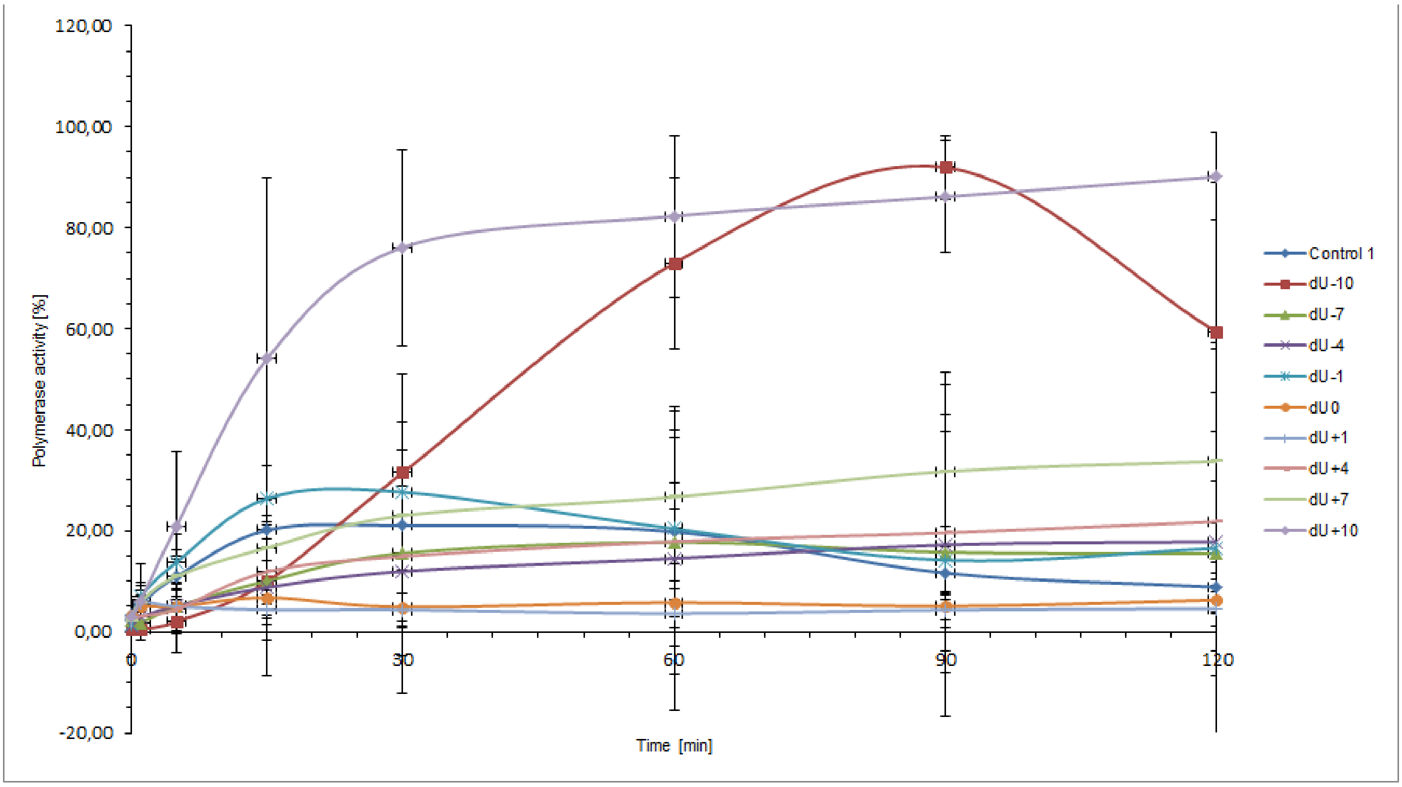

Figure S7. Graphical representation of DNA repair assays' results for RcdA. (A) AP site rejoining efficiency, (B) AP site rejoining efficiency + SD, (C) endonuclease activity, (D) endonuclease activity + SD, (E) polymerase activity, (F) polymerase activity + SD

Table S4. AP site rejoining - RcdA. Raw numerical data of densitometry obtained from Quantity One software.

RcdA		Time [min]							
		0	1	5	15	30	60	90	120
Strand	Data set	AP site rejoining [%]							
dU0	1.	82,61	0,57	0,49	0,99	1,34	1,94	2,21	2,18
	2.	83,19	7,65	0,59	0,44	0,20	0,48	0,16	0,11
	3.	91,79	7,91	0,59	0,44	0,65	1,20	2,71	3,15
	Avg	85,86	5,38	0,56	0,62	0,73	1,21	1,69	1,81
	SD	5,14	4,17	0,06	0,32	0,57	0,73	1,35	1,56
dU-1	1.	89,72	5,37	4,12	9,81	24,68	52,16	64,02	61,39
	2.	93,71	44,18	7,50	13,29	26,71	50,92	62,05	69,45
	3.	98,28	31,06	8,46	17,87	61,85	79,80	88,56	86,38
	Avg	93,91	26,87	6,70	13,66	37,75	60,96	71,54	72,41
	SD	4,28	19,74	2,28	4,04	20,90	16,33	14,77	12,76
dU-4	1.	90,02	41,37	8,62	2,25	2,89	3,70	3,58	5,72
	2.	95,34	34,72	1,57	0,35	0,18	0,07	0,08	0,19
	3.	95,77	37,84	6,58	0,84	0,60	0,52	0,29	0,25
	Avg	70,28	28,48	4,19	0,86	0,92	1,07	0,99	1,54
	SD	3,20	3,33	3,63	0,99	1,46	1,98	1,96	3,17
dU-7	1.	22,43	0,54	0,99	8,79	31,07	53,09	58,20	72,15
	2.	80,76	10,73	1,99	6,24	19,06	36,92	53,68	54,45
	3.	76,31	4,37	1,98	4,06	12,66	34,43	68,41	96,43
	Avg	59,83	5,22	1,65	6,36	20,93	41,48	60,10	74,35
	SD	32,47	5,15	0,57	2,37	9,35	10,13	7,55	21,08
dU-10	1.	98,44	11,75	0,17	0,00	0,00	0,00	0,00	0,00
	2.	91,41	14,68	4,37	3,02	7,70	10,60	14,46	45,06
	3.	94,78	16,50	1,27	0,00	0,95	8,08	1,45	5,26
	Avg	94,87	14,31	1,94	1,01	2,88	6,23	5,30	16,78
	SD	3,52	2,40	2,18	1,74	4,20	5,54	7,96	24,64
dU+1	1.	97,43	3,60	1,27	0,81	0,65	0,51	0,47	0,41
	2.	91,01	12,23	2,25	0,98	0,57	0,53	0,44	0,39
	3.	88,75	13,03	2,31	0,62	0,41	0,39	0,33	2,31
	Avg	92,39	9,62	1,94	0,80	0,54	0,48	0,42	1,04
	SD	4,50	5,23	0,58	0,18	0,12	0,08	0,08	1,10
dU+4	1.	88,13	89,08	86,01	72,85	52,20	31,36	21,87	14,04
	2.	77,89	73,65	64,18	45,02	35,00	21,72	16,15	12,00
	3.	92,81	91,45	84,36	61,54	29,30	12,43	6,40	3,10
	Avg	86,28	84,73	78,18	59,80	38,83	21,84	14,81	9,72
	SD	7,63	9,66	12,15	14,00	11,92	9,47	7,82	5,82
dU+7	1.	94,28	3,75	0,87	2,98	62,29	44,45	65,73	82,05
	2.	91,63	61,31	1,90	1,91	3,67	39,65	47,19	43,87
	3.	87,31	42,25	2,36	1,63	5,98	13,05	43,97	41,09
	Avg	91,07	35,77	1,71	2,17	23,98	32,38	52,30	55,67
	SD	3,52	29,32	0,76	0,72	33,20	16,91	11,74	22,89
dU+10	1.	87,51	66,19	6,65	0,00	0,00	0,14	0,00	0,04
	2.	94,92	82,20	2,94	0,48	0,69	0,11	0,00	0,27
	3.	92,18	86,09	50,18	1,79	0,64	0,70	0,56	0,62
	Avg	91,54	78,16	19,92	0,75	0,44	0,32	0,19	0,31
	SD	3,75	10,55	26,27	0,93	0,38	0,33	0,32	0,29

Table S5. Endonuclease activity - RcdA. Raw numerical data of densitometry obtained from Quantity One software.

RcdA		Time [min]							
		0	1	5	15	30	60	90	120
Strand	Data set	Endonuclease activity [%]							
dU0	1.	10,28	73,05	72,31	71,40	70,97	71,59	71,33	70,12
	2.	4,39	74,20	79,27	75,50	79,63	77,94	73,91	75,92
	3.	2,17	86,58	93,04	90,44	89,57	87,57	87,05	84,92
	Avg	5,62	77,94	81,54	79,11	80,06	79,03	77,43	76,99
	SD	4,19	7,50	10,55	10,02	9,31	8,05	8,43	7,46
dU-1	1.	1,21	71,34	66,57	50,99	35,00	19,02	14,47	14,64
	2.	0,25	47,58	75,59	60,31	42,40	21,90	14,69	8,25
	3.	0,06	62,89	76,58	49,08	10,81	3,09	4,60	2,15
	Avg	0,51	60,60	72,92	53,46	29,40	14,67	11,25	8,34
	SD	0,62	12,04	5,52	6,01	16,52	10,13	5,76	6,24
dU-4	1.	2,51	42,41	63,87	56,46	48,85	38,73	31,54	25,09
	2.	4,66	62,83	89,96	90,44	93,83	92,56	92,62	91,19
	3.	2,37	57,59	85,88	88,64	89,11	88,95	91,08	89,47
	Avg	2,38	40,71	59,93	58,88	57,95	55,06	53,81	51,44
	SD	1,28	10,60	14,03	19,12	24,72	30,09	34,83	37,68
dU-7	1.	48,99	74,65	64,07	47,31	26,51	11,60	13,19	3,82
	2.	7,47	82,55	83,25	65,40	39,12	13,03	1,71	1,29
	3.	23,69	95,63	98,02	95,94	87,34	65,57	31,59	3,57
	Avg	26,72	84,28	81,78	69,55	50,99	30,06	15,50	2,89
	SD	20,93	10,60	17,02	24,58	32,10	30,75	15,07	1,39
dU-10	1.	1,21	87,62	97,31	93,94	86,02	39,24	3,70	66,13
	2.	6,98	82,83	87,61	77,47	46,56	9,65	0,76	1,28
	3.	3,68	82,06	95,51	84,76	54,85	5,57	0,18	0,00
	Avg	3,95	84,17	93,47	85,39	62,47	18,15	1,55	22,47
	SD	2,90	3,02	5,16	8,25	20,81	18,38	1,89	37,82
dU+1	1.	0,45	88,46	87,41	86,67	83,70	79,70	77,33	73,30
	2.	0,70	72,46	78,35	78,33	75,70	69,56	63,88	60,56
	3.	1,69	65,55	71,65	71,43	68,67	60,98	57,95	54,26
	Avg	0,95	75,49	79,14	78,81	76,03	70,08	66,38	62,71
	SD	0,66	11,75	7,91	7,63	7,52	9,37	9,93	9,70
dU+4	1.	2,09	2,49	3,63	9,81	20,48	35,19	45,27	54,32
	2.	0,25	2,15	6,95	15,51	16,06	16,76	18,83	17,74
	3.	0,86	0,92	2,75	14,39	36,33	54,91	60,24	63,91
	Avg	1,06	1,85	4,44	13,24	24,29	35,62	41,45	45,32
	SD	0,94	0,83	2,21	3,02	10,66	19,08	20,97	24,37
dU+7	1.	1,41	77,71	75,24	68,31	22,78	33,15	18,81	8,67
	2.	1,50	26,38	76,37	67,42	48,53	17,98	10,02	8,21
	3.	2,93	47,46	83,14	80,49	71,86	55,30	13,11	9,80
	Avg	1,94	50,52	78,25	72,07	47,72	35,48	13,98	8,89
	SD	0,85	25,80	4,27	7,31	24,55	18,77	4,46	0,82
dU+10	1.	0,92	18,43	76,73	68,64	31,69	16,83	18,75	8,05
	2.	0,43	9,35	78,33	58,32	34,31	32,24	19,47	17,27
	3.	0,05	0,07	0,00	0,10	0,00	0,00	0,01	0,02
	Avg	0,47	9,28	51,69	42,36	22,00	16,36	12,74	8,45
	SD	0,43	9,18	44,77	36,95	19,10	16,13	11,04	8,63

Table S6. Polymerase activity - RcdA. Raw numerical data of densitometry obtained from Quantity One software.

RcdA		Time [min]							
		0	1	5	15	30	60	90	120
Strand	Data set	Polymerase activity [%]							
dU0	1.	2,99	5,37	4,85	5,79	3,55	4,52	3,87	5,34
	2.	5,57	5,54	4,66	6,35	3,18	3,95	3,32	4,18
	3.	1,42	4,31	5,45	8,24	8,08	8,92	8,06	9,42
	Avg	3,33	5,08	4,99	6,79	4,94	5,80	5,09	6,31
	SD	2,09	0,67	0,42	1,28	2,73	2,72	2,59	2,75
dU-1	1.	2,53	14,44	20,14	30,06	32,85	23,28	17,04	17,50
	2.	1,46	3,01	9,92	18,96	24,32	21,99	19,06	21,07
	3.	0,32	3,09	11,34	30,24	25,81	16,03	6,42	11,13
	Avg	1,43	6,85	13,80	26,42	27,66	20,44	14,17	16,57
	SD	1,10	6,58	5,53	6,46	4,56	3,87	6,79	5,03
dU-4	1.	2,79	8,77	17,58	32,09	43,72	53,93	62,37	67,83
	2.	0,00	0,53	0,35	1,16	2,44	2,23	4,24	1,37
	3.	1,87	2,04	2,63	1,89	1,72	1,99	2,38	2,22
	Avg	1,16	2,84	5,14	8,78	11,97	14,54	17,25	17,86
	SD	1,42	4,39	9,36	17,65	24,04	29,92	34,11	38,13
dU-7	1.	4,62	3,24	5,58	7,35	8,02	5,58	3,92	3,00
	2.	2,04	2,16	9,42	22,84	38,71	47,66	43,39	43,45
	3.	0,00	0,00	0,00	0,00	0,00	0,00	0,00	0,00
	Avg	2,22	1,80	5,00	10,07	15,58	17,75	15,77	15,49
	SD	2,32	1,65	4,74	11,66	20,43	26,05	24,00	24,27
dU-10	1.	0,08	0,04	0,28	2,28	9,09	53,70	94,38	33,05
	2.	1,04	1,05	4,15	15,30	42,98	79,68	84,78	53,65
	3.	0,41	0,52	1,58	12,38	42,48	85,55	96,74	91,26
	Avg	0,51	0,54	2,01	9,99	31,52	72,97	91,97	59,32
	SD	0,49	0,51	1,97	6,83	19,42	16,95	6,34	29,52
dU+1	1.	0,87	1,60	1,02	0,81	0,53	0,47	0,34	0,64
	2.	1,86	6,69	6,34	6,06	6,75	5,90	7,48	6,61
	3.	3,49	9,17	7,75	6,23	5,78	4,63	5,18	6,52
	Avg	2,07	5,82	5,03	4,37	4,35	3,67	4,33	4,59
	SD	1,33	3,86	3,55	3,08	3,35	2,84	3,64	3,42
dU+4	1.	3,07	2,28	2,17	2,09	1,31	0,32	0,00	0,00
	2.	3,62	6,29	10,10	20,48	29,24	40,61	45,71	50,04
	3.	0,73	0,76	1,09	13,09	14,23	12,73	13,14	15,47
	Avg	2,47	3,11	4,46	11,89	14,93	17,89	19,62	21,84
	SD	1,53	2,86	4,92	9,25	13,98	20,63	23,53	25,62
dU+7	1.	1,76	7,38	12,00	17,32	8,97	13,94	12,05	6,69
	2.	3,06	6,93	14,35	22,62	44,17	40,53	41,36	46,63
	3.	1,44	3,05	6,68	10,04	15,86	25,60	41,89	47,93
	Avg	2,09	5,79	11,01	16,66	23,00	26,69	31,77	33,75
	SD	0,86	2,38	3,93	6,31	18,66	13,33	17,07	23,44
dU+10	1.	4,98	8,31	12,53	28,41	66,61	81,58	79,87	89,65
	2.	1,90	4,14	12,65	38,85	63,34	66,66	79,55	81,75
	3.	1,95	5,53	37,83	94,98	98,37	98,72	99,04	98,90
	Avg	2,95	5,99	21,00	54,08	76,11	82,32	86,15	90,10
	SD	1,76	2,12	14,57	35,80	19,35	16,04	11,16	8,58

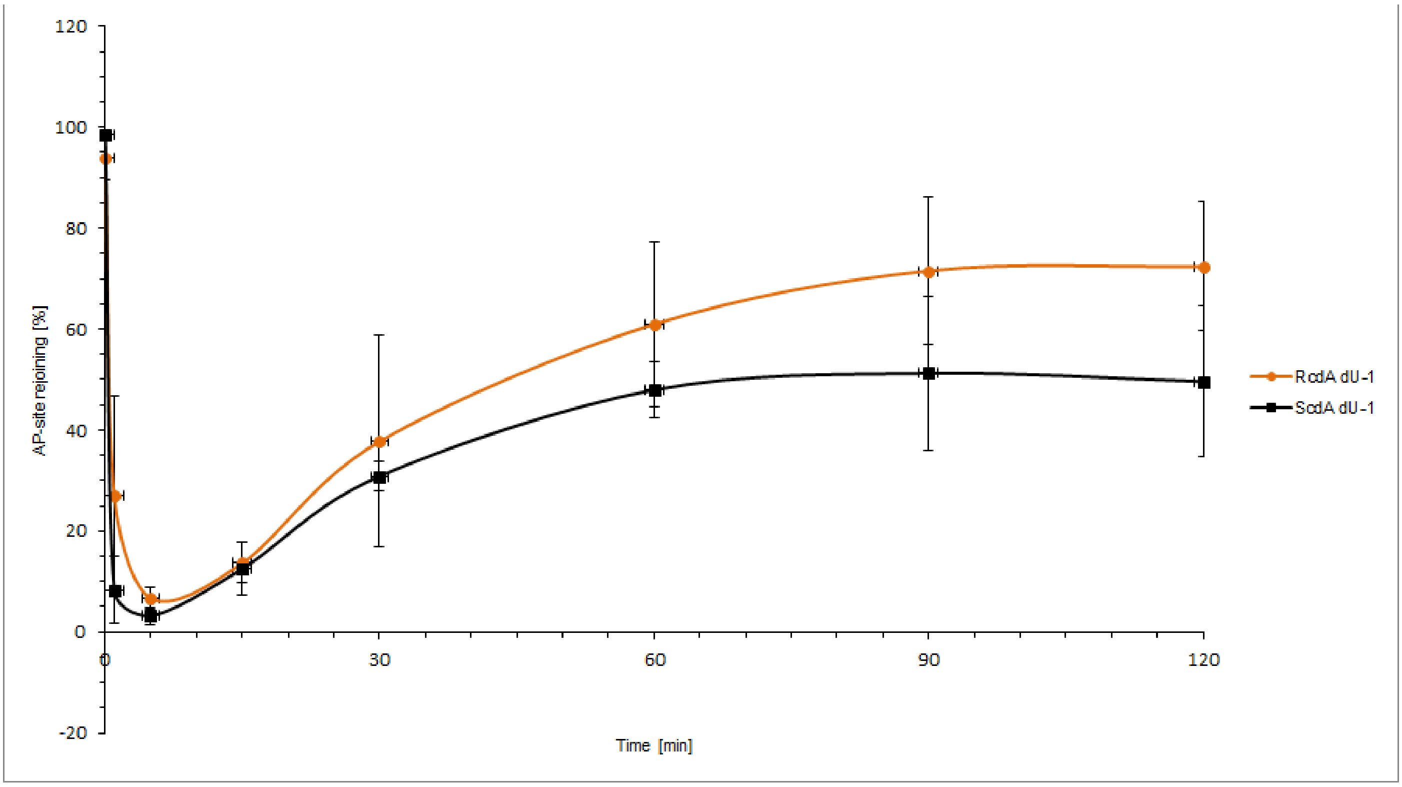
120
100
80
60
40
20
0
-20
AP-site rejoining [%]
0
30
60
90
120
Time [min]
RcdA dU-1
ScdA dU-1

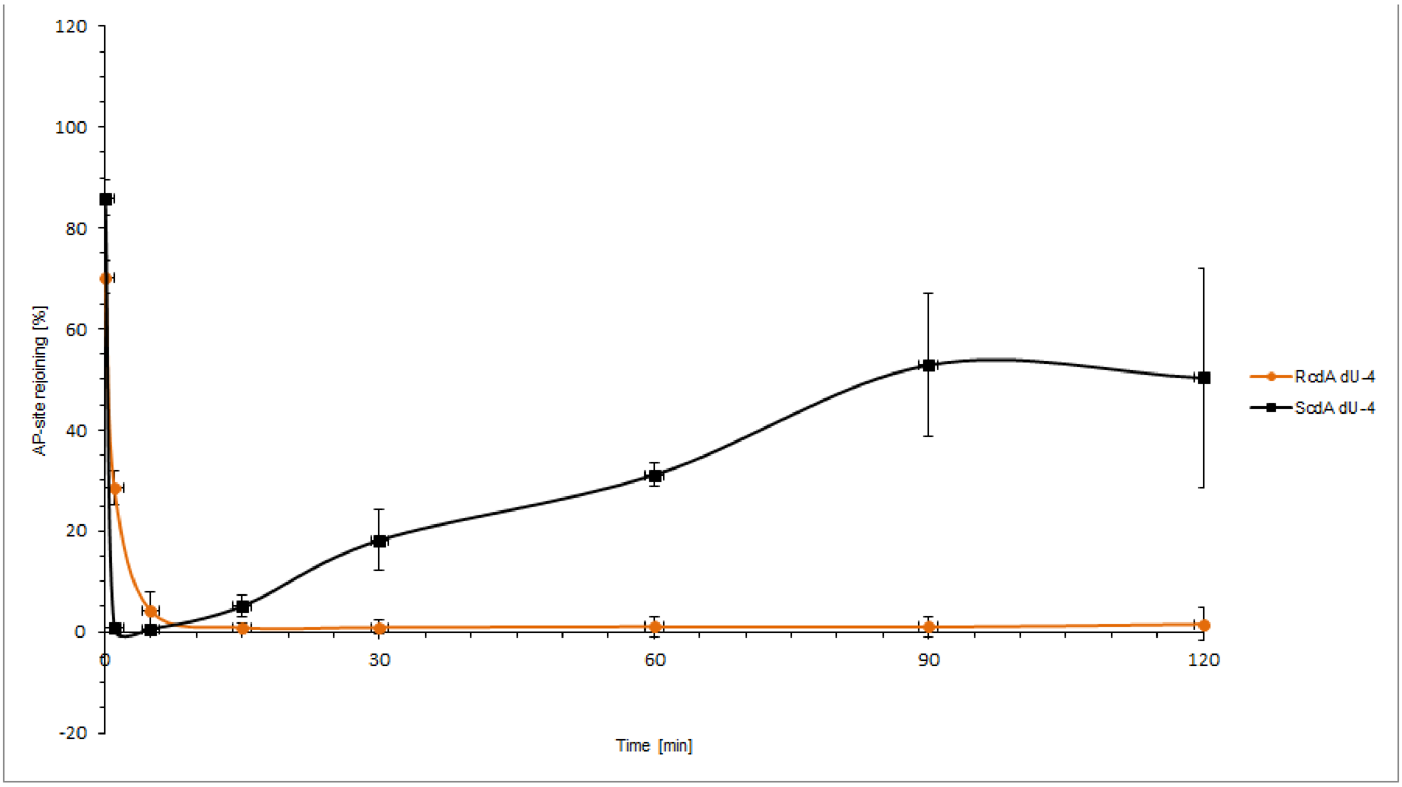
120
100
80
60
40
20
0
-20
AP-site rejoining [%]
0
30
60
90
120
Time [min]
RcdA dU-4
ScdA dU-4

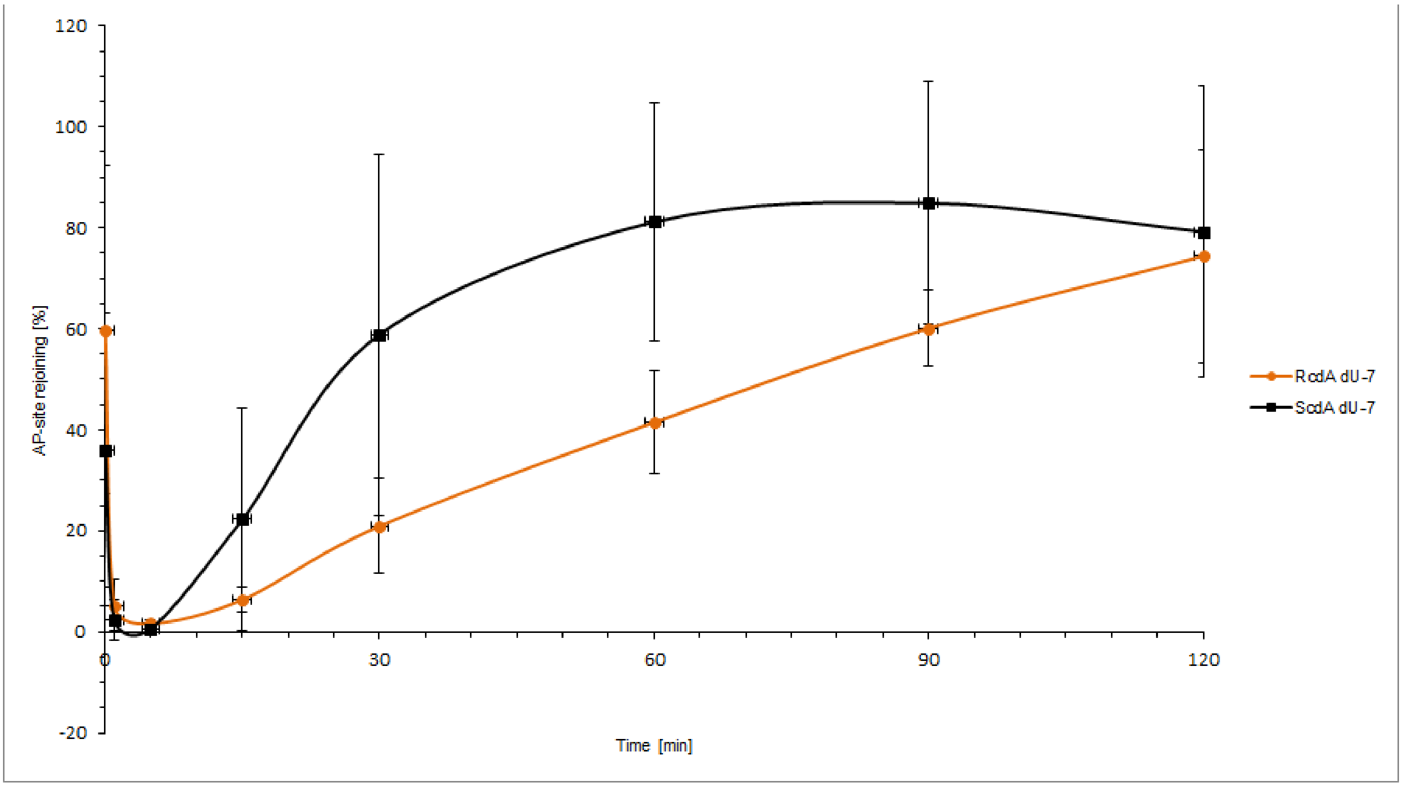
120
100
80
60
40
20
0
-20
AP-site rejoining [%]
0
30
60
90
120
Time [min]
RcdA dU-7
ScdA dU-7

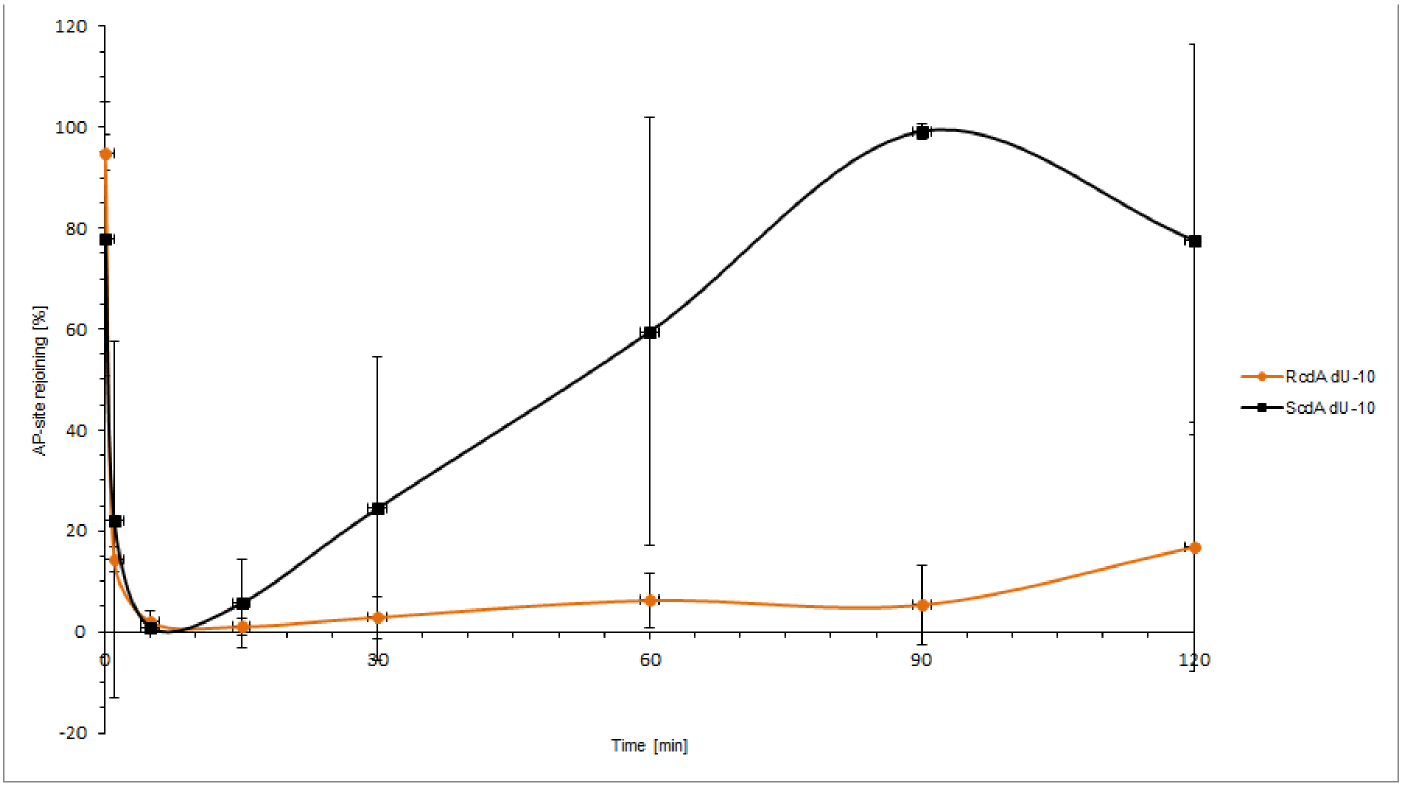
120
100
80
60
40
20
0
-20
AP-site rejoining [%]
0
30
60
90
120
Time [min]
RcdA dU-10
ScdA dU-10

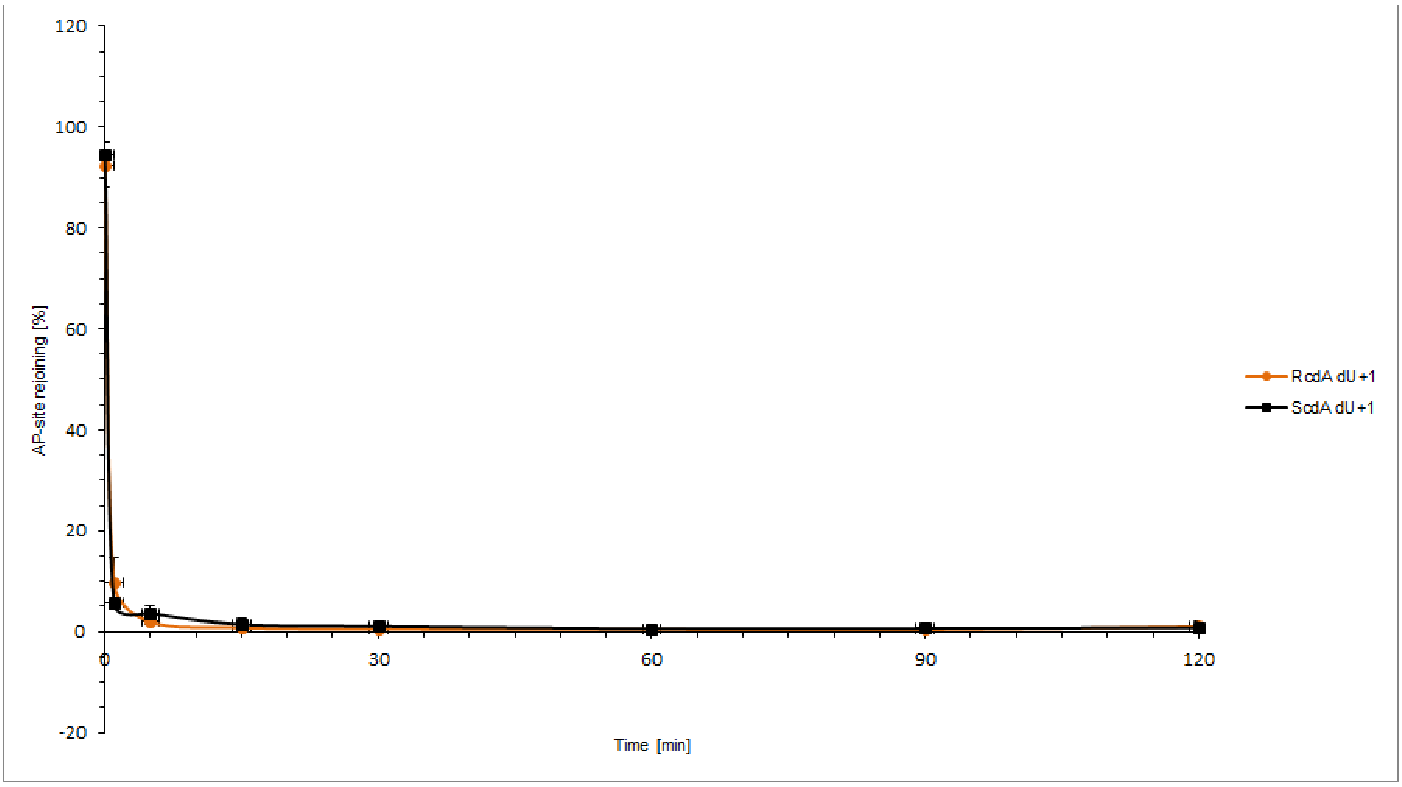

120
100
80
60
40
20
0
-20
AP-site rejoining [%]
0
30
60
90
120
Time [min]
RcdA dU+1
ScdA dU+1

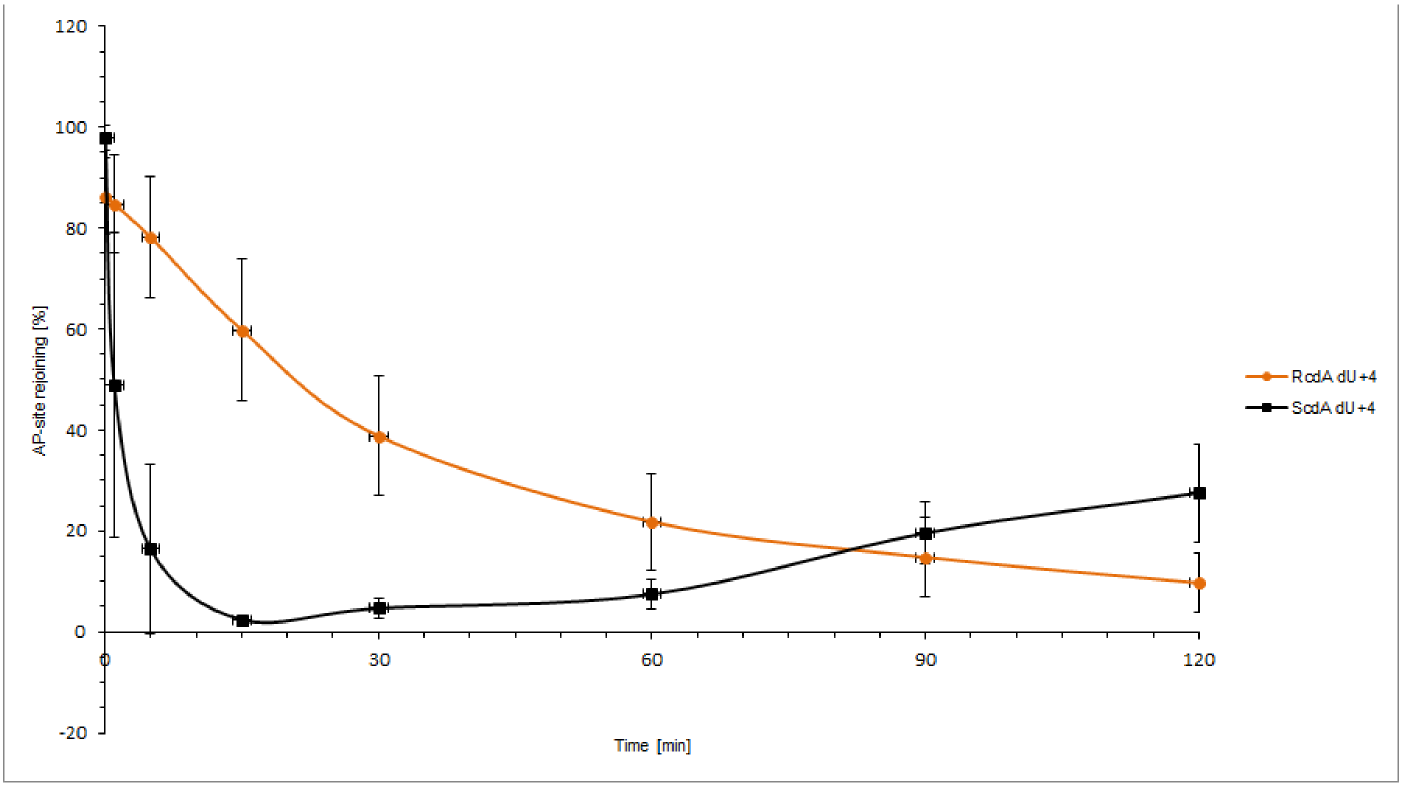

120
100
80
60
40
20
0
-20
AP-site rejoining [%]
0
30
60
90
120
Time [min]
RcdA dU+4
ScdA dU+4

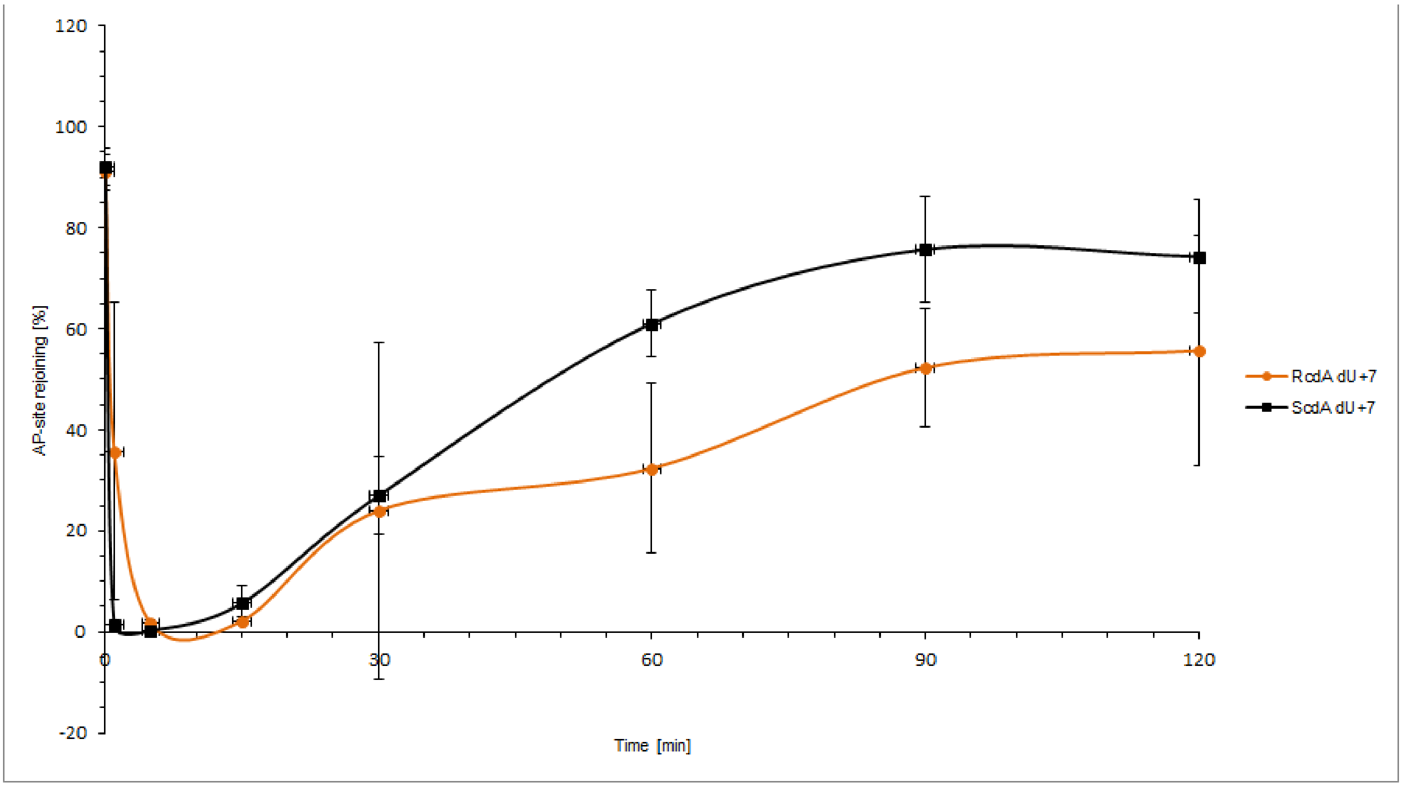

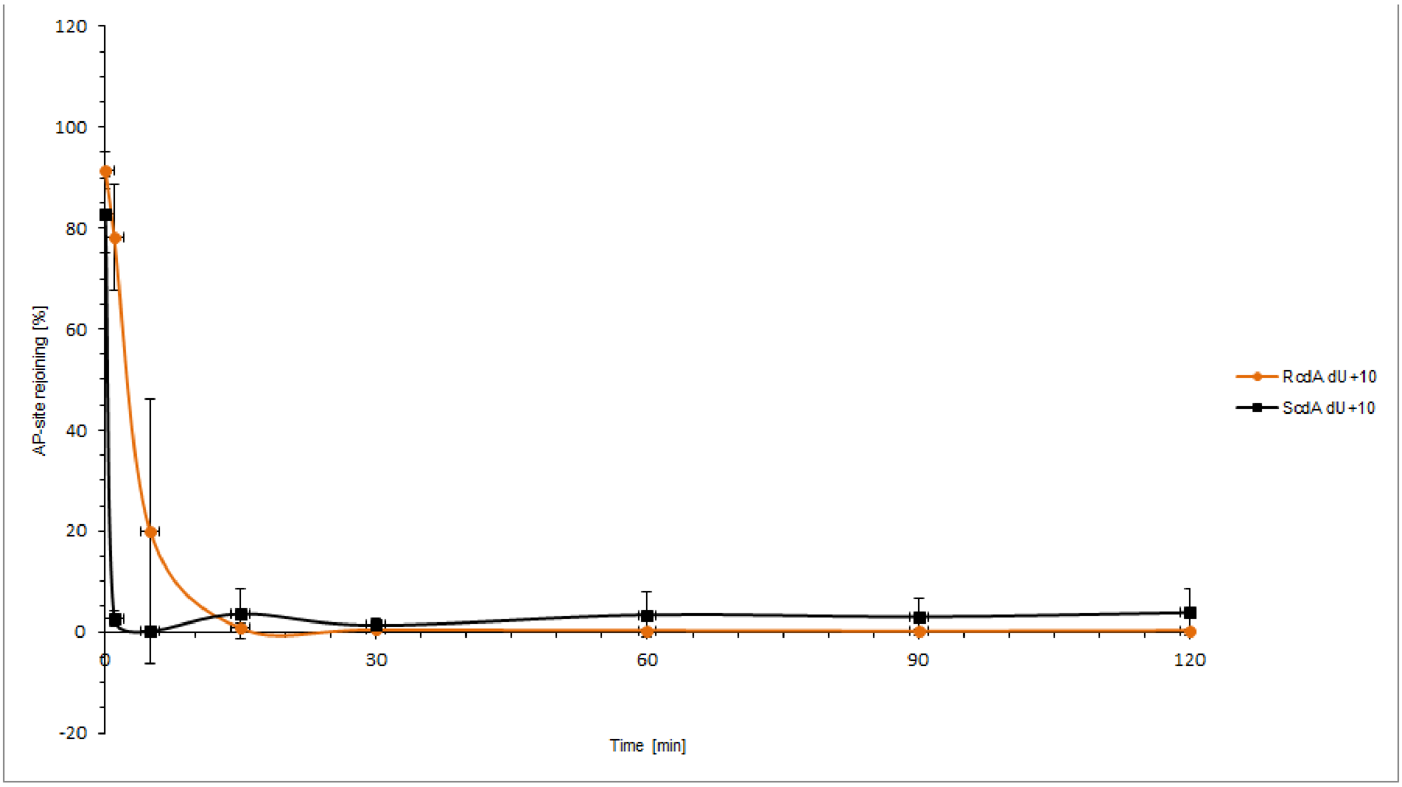

Figure S8. AP site rejoining [%] of ScdA vs. RcdA – comparison of individual strands

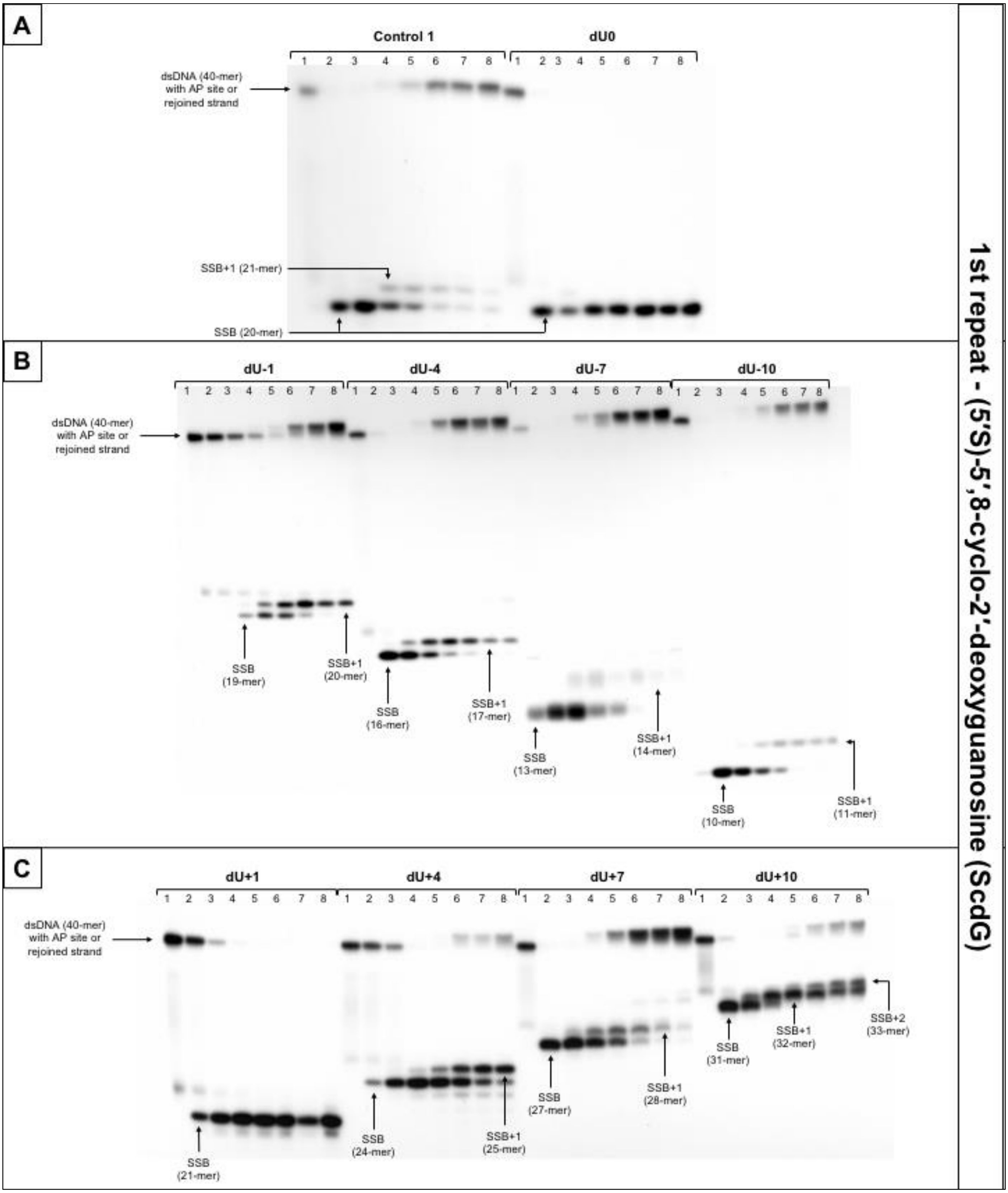
A
Control 1
dU0
dsDNA (40-mer) with AP site or rejoined strand
SSB+1 (21-mer)
SSB (20-mer)
B
dU-1
dU-4
dU-7
dU-10
dsDNA (40-mer) with AP site or rejoined strand
SSB (19-mer)
SSB+1 (20-mer)
SSB (16-mer)
SSB+1 (17-mer)
SSB (13-mer)
SSB+1 (14-mer)
SSB (10-mer)
SSB+1 (11-mer)
C
dU+1
dU+4
dU+7
dU+10
dsDNA (40-mer) with AP site or rejoined strand
SSB (21-mer)
SSB (24-mer)
SSB+1 (25-mer)
SSB (27-mer)
SSB+1 (28-mer)
SSB (31-mer)
SSB+1 (32-mer)
SSB+2 (33-mer)
1st repeat - (5'S)-5',8-cyclo-2'-deoxyguanosine (ScdG)

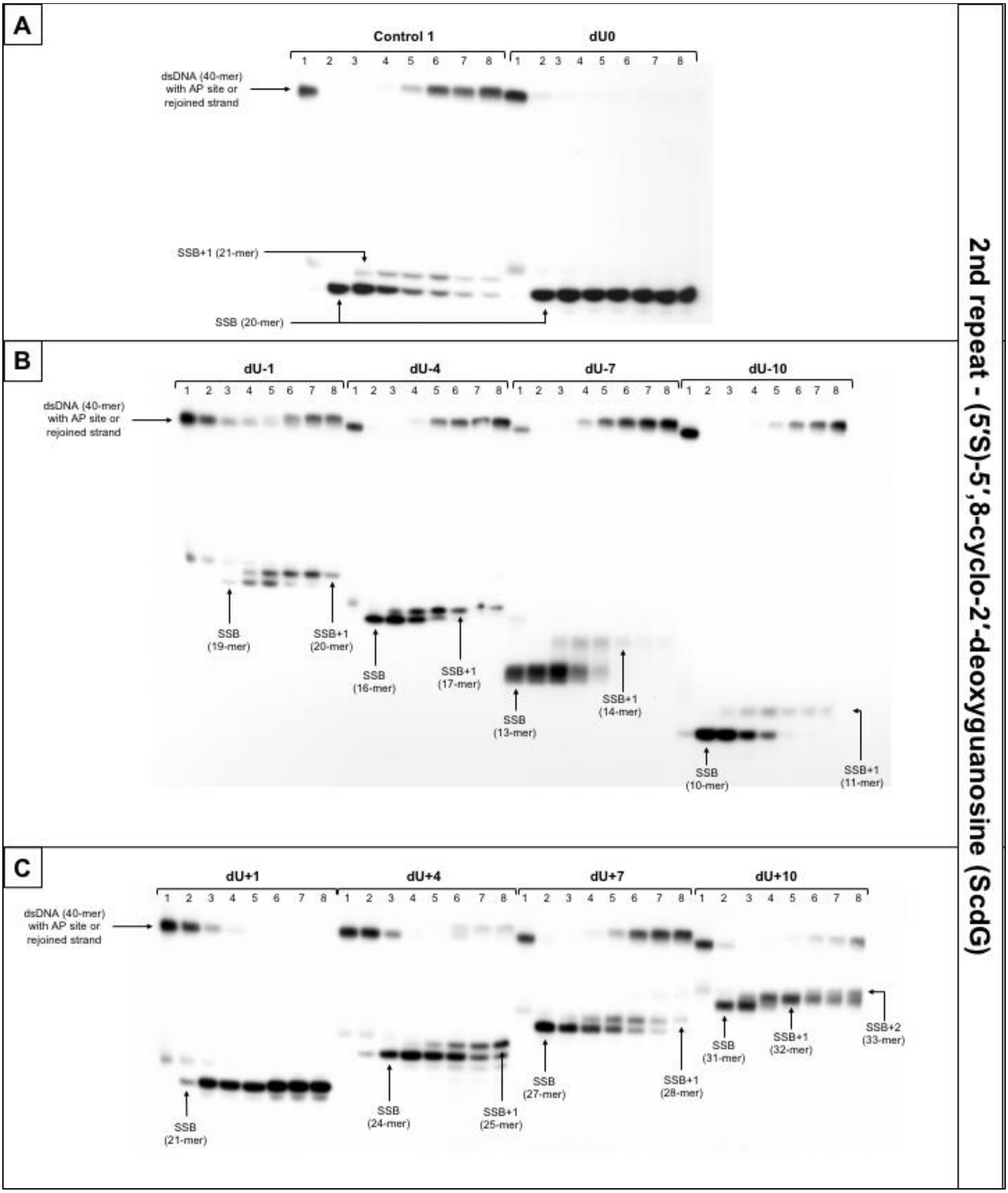
A
Control 1
dU0
1 2 3 4 5 6 7 8
dsDNA (40-mer) with AP site or rejoined strand
SSB+1 (21-mer)
SSB (20-mer)
B
dU-1
dU-4
dU-7
dU-10
SSB (19-mer)
SSB+1 (20-mer)
SSB (16-mer)
SSB+1 (17-mer)
SSB (13-mer)
SSB+1 (14-mer)
SSB (10-mer)
SSB+1 (11-mer)
C
dU+1
dU+4
dU+7
dU+10
SSB (21-mer)
SSB (24-mer)
SSB+1 (25-mer)
SSB (27-mer)
SSB+1 (28-mer)
SSB (31-mer)
SSB+1 (32-mer)
SSB+2 (33-mer)
2nd repeat - (5'S)-5',8-cyclo-2'-deoxyguanosine (ScdG)

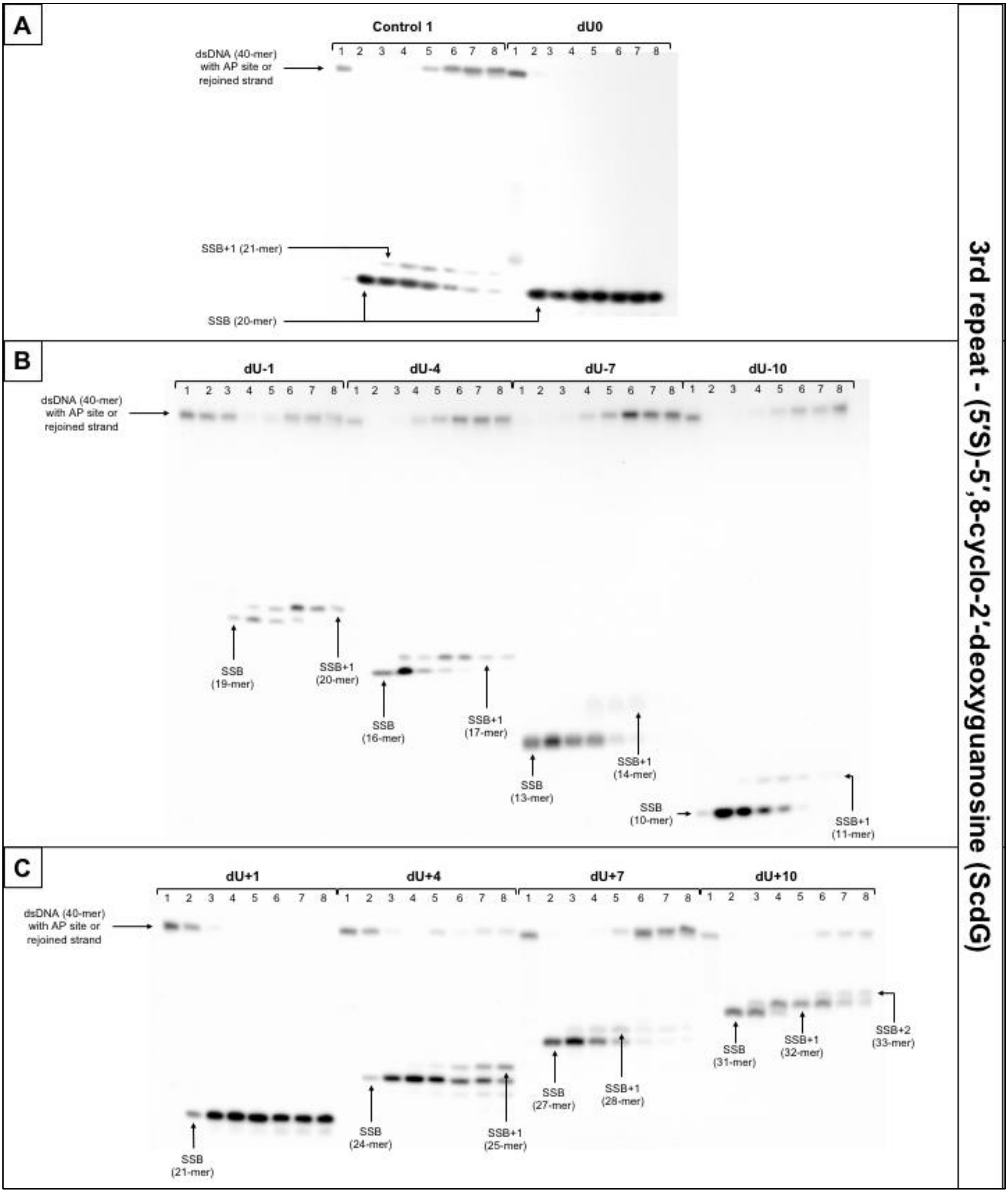

Figure S9. The autoradiograms of denaturing PAGE presenting repair of dsDNA containing clustered damage with AP site in one strand and ScdG in the opposing strand: (A) Controls: dsDNA with single lesion in one strand (Control 1); dsDNA with clustered lesions in two strands opposite to each other (dU0); (B) dsDNA with clustered lesions in two strands where AP site is located 1-10 base pairs in 3′ direction (negative numbers); (C) dsDNA with clustered lesions in two strands where AP site is located 1-10 base pairs in 5′ direction (positive numbers). Each lane corresponds with different assay time: lane 1 - 0 min; lane 2 - 1 min; lane 3 – 5 min; lane 4 – 15 min; lane 5 – 30 min; lane 6 – 60 min; lane 7 – 90 min; lane 8 – 120 min. Each replication of the experiment is shown.

A.

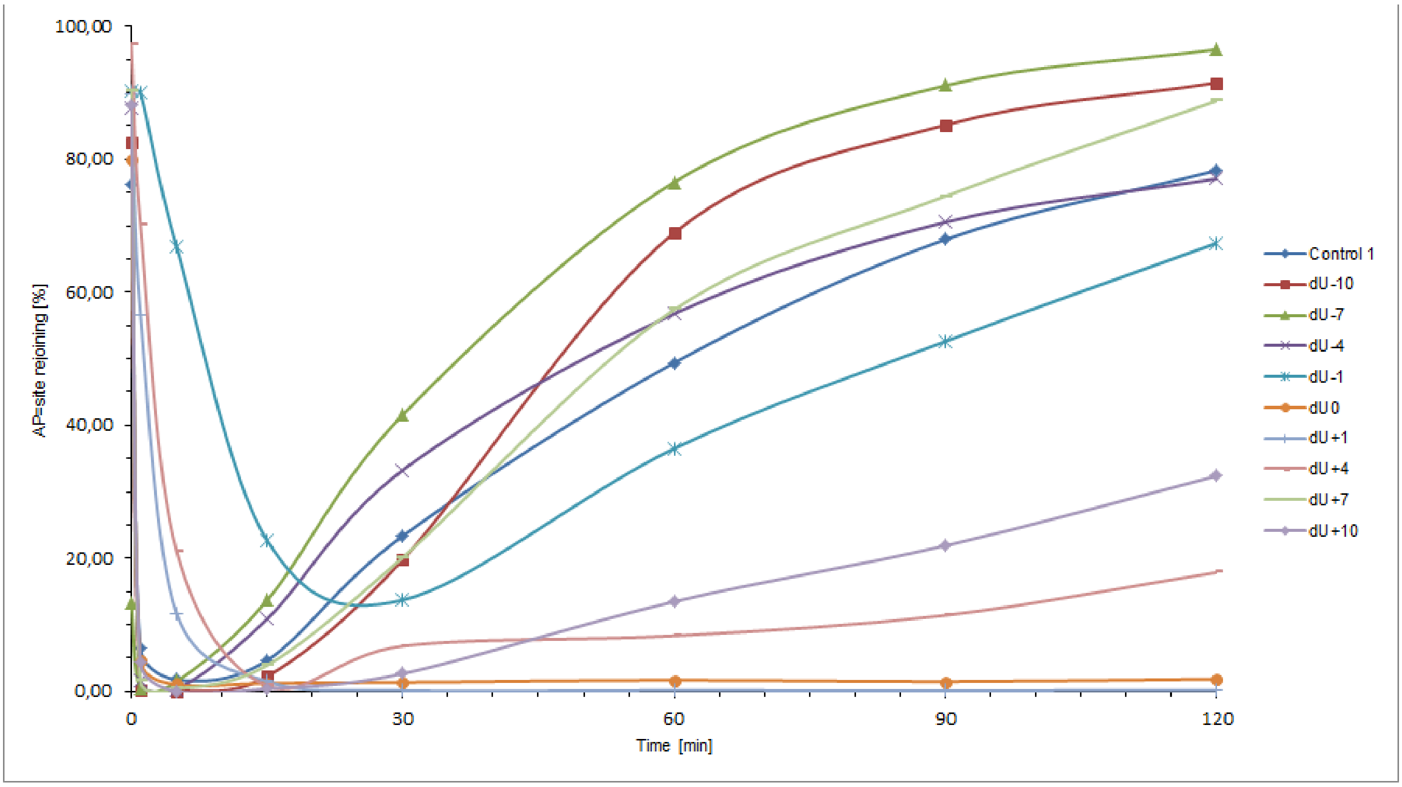

100,00
80,00
60,00
40,00
20,00
0,00
AP=site rejoining [%]
0
30
60
90
120
Time [min]
Control 1
dU-10
dU-7
dU-4
dU-1
dU 0
dU+1
dU+4
dU+7
dU+10

B.

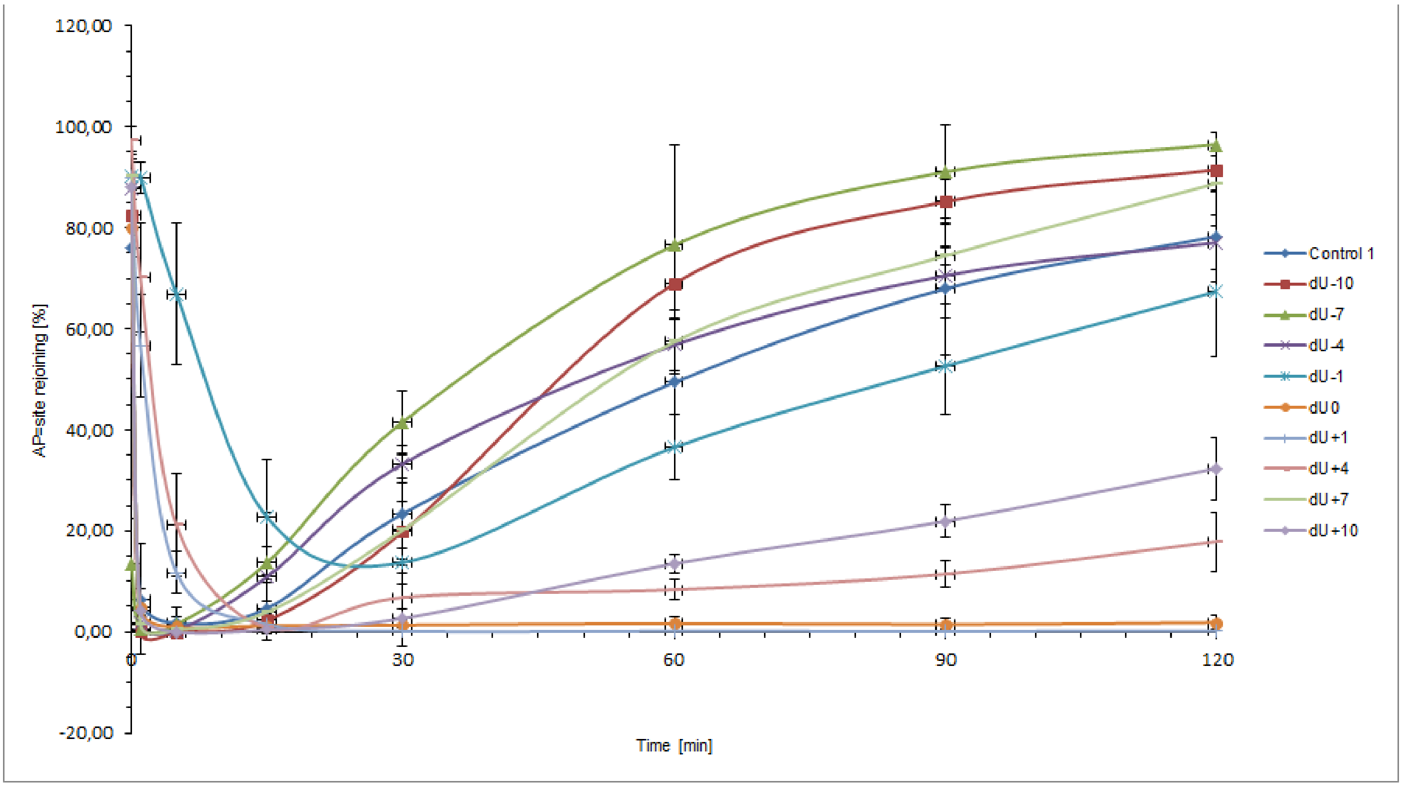

120,00
100,00
80,00
60,00
40,00
20,00
0,00
-20,00
AP=site rejoining [%]
0
30
60
90
120
Time [min]
Control 1
dU-10
dU-7
dU-4
dU-1
dU 0
dU+1
dU+4
dU+7
dU+10

C.

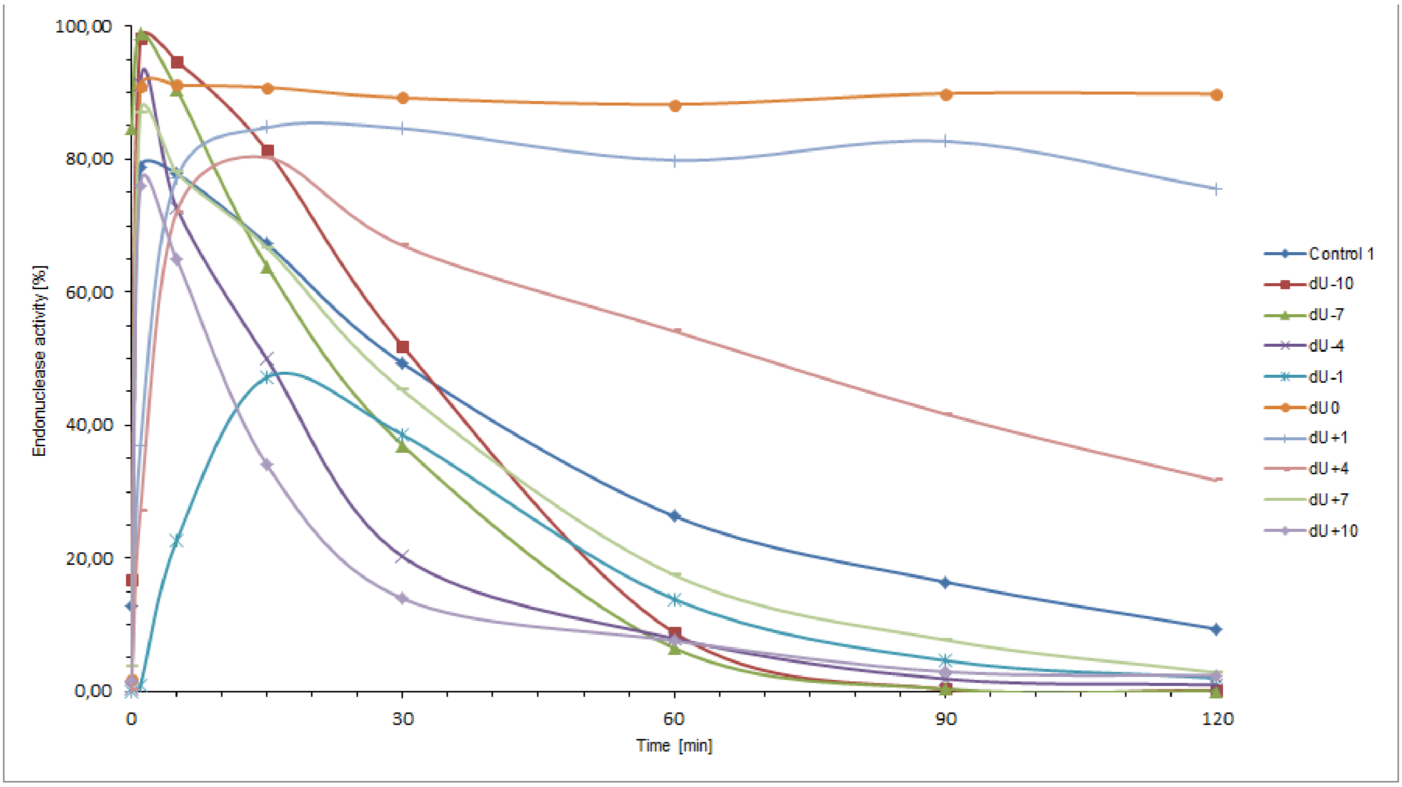

100,00
80,00
60,00
40,00
20,00
0,00
Endonuclease activity [%]
0
30
60
90
120
Time [min]
Control 1
dU-10
dU-7
dU-4
dU-1
dU0
dU+1
dU+4
dU+7
dU+10

D.

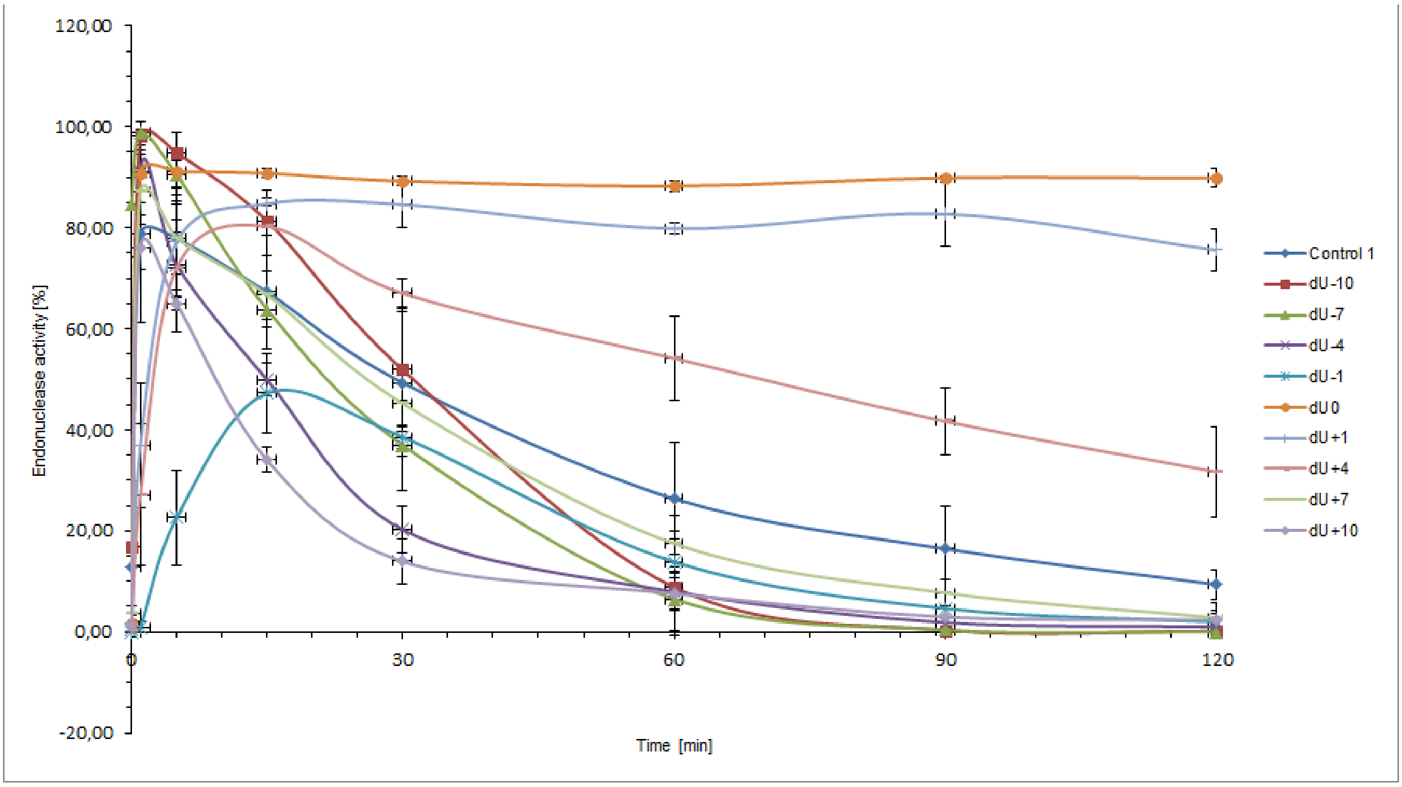

120,00
100,00
80,00
60,00
40,00
20,00
0,00
-20,00
Endonuclease activity [%]
0
30
60
90
120
Time [min]
Control 1
dU-10
dU-7
dU-4
dU-1
dU0
dU+1
dU+4
dU+7
dU+10

E.

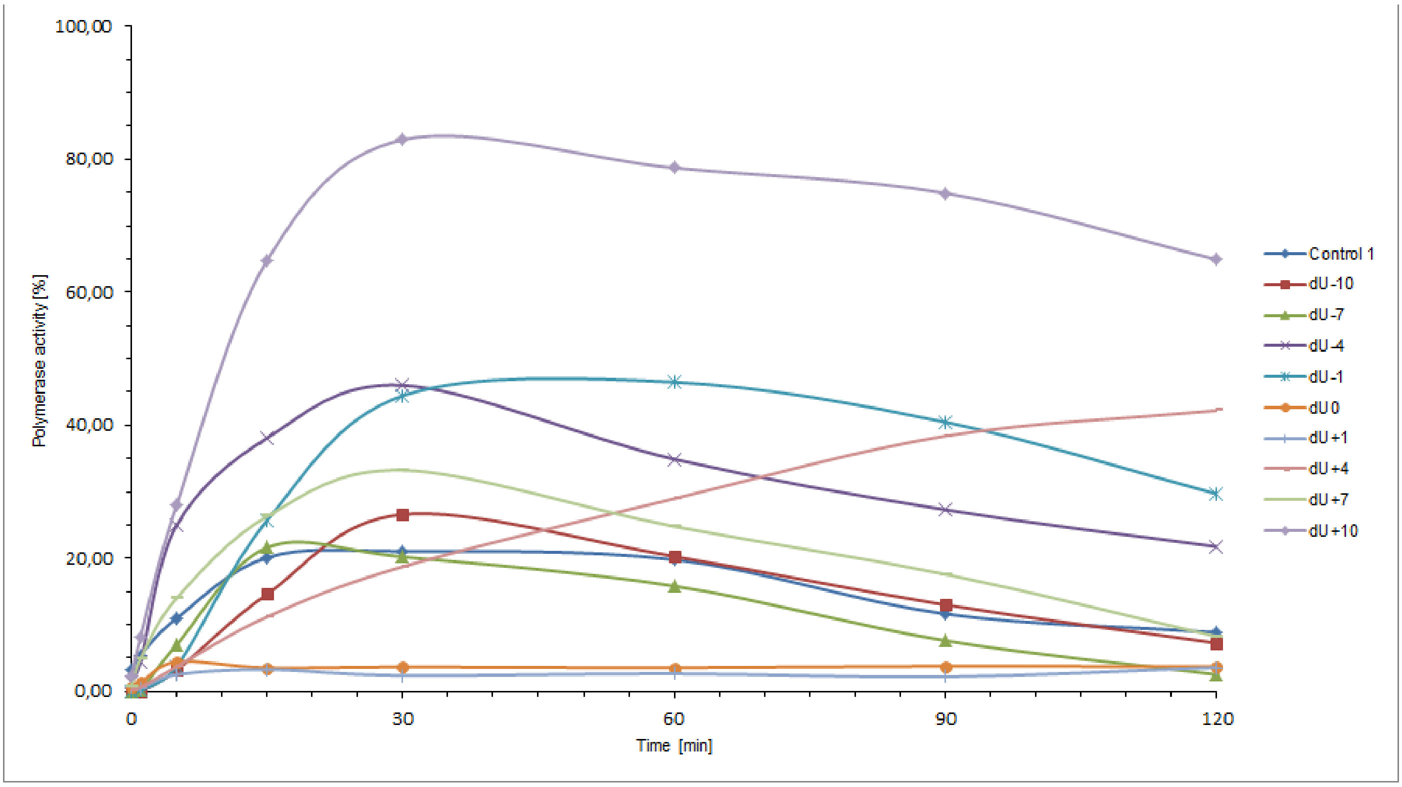

F.

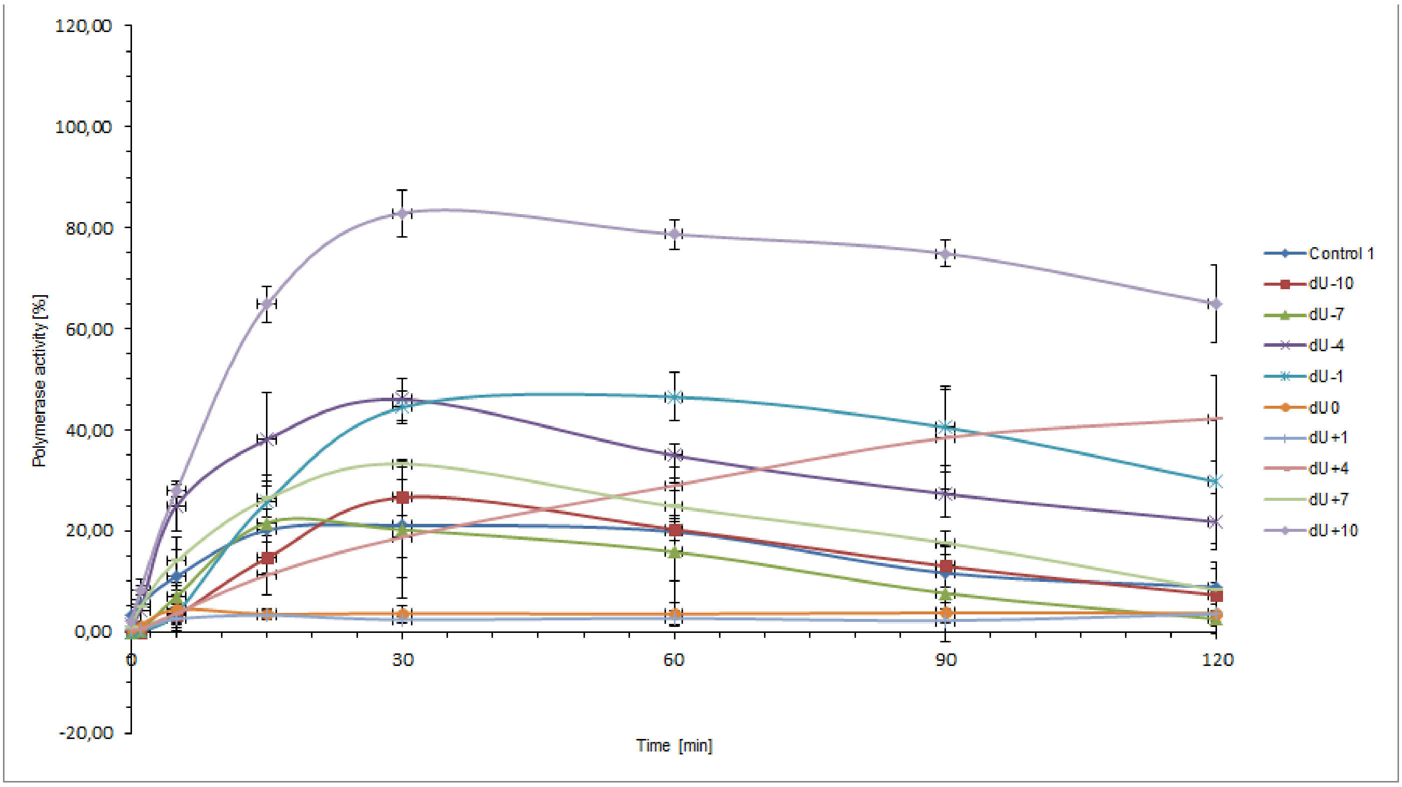

Figure S10. Graphical representation of DNA repair assays' results for ScdG. (A) AP site rejoining efficiency, (B) AP site rejoining efficiency + SD, (C) endonuclease activity, (D) endonuclease activity + SD, (E) polymerase activity, (F) polymerase activity + SD

Table S7. AP site rejoining - ScdG. Raw numerical data of densitometry obtained from Quantity One software.

ScdG		Time [min]							
		0	1	5	15	30	60	90	120
Strand	Data set	AP site rejoining [%]							
dU0	1.	73,69	5,79	0,48	1,05	1,37	1,79	1,93	2,68
	2.	84,75	5,54	1,86	2,19	2,38	3,01	2,24	2,53
	3.	81,39	2,76	0,70	0,39	0,33	0,24	0,27	0,33
	Avg	79,94	4,70	1,02	1,21	1,36	1,68	1,48	1,85
	SD	5,67	1,68	0,74	0,91	1,03	1,39	1,06	1,32
dU-1	1.	92,37	93,42	70,59	28,69	14,23	37,97	62,16	72,93
	2.	82,42	87,40	78,55	29,94	13,77	42,05	52,59	76,76
	3.	95,84	89,22	51,32	9,54	13,21	29,55	43,10	52,53
	Avg	90,21	90,01	66,82	22,72	13,74	36,52	52,62	67,41
	SD	6,97	3,09	14,00	11,43	0,51	6,37	9,53	13,02
dU-4	1.	88,76	3,12	0,00	3,80	35,60	62,61	75,44	81,05
	2.	81,28	1,96	0,48	3,42	29,01	54,49	64,40	79,37
	3.	93,05	0,00	0,43	25,43	35,20	53,25	71,80	70,81
	Avg	87,70	1,69	0,30	10,88	33,27	56,78	70,54	77,07
	SD	5,96	1,58	0,26	12,60	3,69	5,08	5,62	5,49
dU-7	1.	20,28	0,68	0,80	16,20	46,49	84,64	94,87	96,82
	2.	16,57	0,45	0,58	10,06	43,49	91,02	97,79	98,61
	3.	3,24	0,23	3,11	14,76	34,76	54,02	80,57	94,11
	Avg	13,36	0,45	1,50	13,68	41,58	76,56	91,08	96,51
	SD	8,96	0,23	1,40	3,21	6,10	19,78	9,22	2,27
dU-10	1.	92,54	0,00	0,00	2,52	30,78	76,62	82,27	86,71
	2.	86,42	0,26	0,13	0,82	9,88	67,83	83,02	93,01
	3.	68,32	0,00	0,00	3,41	18,82	62,32	90,18	94,76
	Avg	82,43	0,09	0,04	2,25	19,83	68,92	85,16	91,49
	SD	12,59	0,15	0,08	1,32	10,49	7,21	4,36	4,23
dU+1	1.	85,57	48,91	11,20	0,67	0,16	0,19	0,00	0,32
	2.	85,50	68,14	16,17	3,71	0,19	0,19	0,26	0,16
	3.	98,32	52,97	7,75	0,08	0,08	0,08	0,09	0,06
	Avg	89,80	56,67	11,71	1,49	0,14	0,15	0,12	0,18
	SD	7,38	10,14	4,24	1,94	0,06	0,06	0,13	0,13
dU+4	1.	98,90	65,36	26,68	1,05	1,39	10,04	11,06	24,53
	2.	94,17	82,49	27,17	1,17	1,11	9,00	8,93	13,42
	3.	98,69	62,74	9,39	0,28	17,97	6,03	14,35	15,79
	Avg	97,25	70,19	21,08	0,83	6,83	8,35	11,45	17,91
	SD	2,67	10,72	10,13	0,49	9,65	2,08	2,73	5,85
dU+7	1.	92,43	1,76	0,89	6,34	26,78	59,08	75,90	88,44
	2.	87,12	1,40	0,38	3,47	16,90	50,48	75,06	90,70
	3.	90,86	1,56	0,00	1,96	16,76	62,59	72,39	87,24
	Avg	90,14	1,57	0,43	3,92	20,15	57,38	74,45	88,79
	SD	2,73	0,18	0,45	2,23	5,75	6,23	1,83	1,76
dU+10	1.	79,11	4,35	0,03	0,11	4,70	12,75	20,74	25,63
	2.	86,71	8,57	0,04	1,05	2,21	12,16	19,32	33,80
	3.	98,26	0,17	0,03	0,05	1,12	15,61	25,68	37,74
	Avg	88,03	4,36	0,03	0,41	2,68	13,51	21,91	32,39
	SD	9,64	4,20	0,00	0,56	1,84	1,84	3,34	6,18

Table S8. Endonuclease activity - ScdG. Raw numerical data of densitometry obtained from Quantity One software.

ScdG		Time [min]							
		0	1	5	15	30	60	90	120
Strand	Data set	Endonuclease activity [%]							
dU0	1.	3,54	87,70	86,68	91,72	90,01	87,48	90,29	88,64
	2.	0,50	90,35	90,79	90,61	89,61	87,79	89,63	88,81
	3.	1,54	94,77	95,84	90,05	88,12	89,50	89,72	92,03
	Avg	1,86	90,94	91,10	90,79	89,25	88,26	89,88	89,83
	SD	1,54	3,57	4,59	0,85	1,00	1,09	0,36	1,91
dU-1	1.	0,00	0,23	21,87	45,12	36,49	11,60	1,90	1,07
	2.	0,00	0,28	13,64	40,67	38,17	9,39	4,24	0,96
	3.	0,01	2,19	32,36	55,92	41,07	20,58	7,76	3,92
	Avg	0,00	0,90	22,63	47,24	38,58	13,86	4,63	1,98
	SD	0,00	1,12	9,38	7,84	2,32	5,93	2,95	1,68
dU-4	1.	0,60	93,16	80,19	49,97	14,76	3,84	0,98	0,40
	2.	0,65	84,33	68,34	53,22	22,63	10,74	3,08	1,65
	3.	0,17	96,27	69,53	46,57	23,30	9,27	1,53	0,90
	Avg	0,47	91,26	72,69	49,92	20,23	7,95	1,86	0,98
	SD	0,26	6,19	6,53	3,33	4,75	3,63	1,09	0,63
dU-7	1.	78,12	98,97	91,07	63,54	47,22	3,55	0,25	0,07
	2.	78,95	98,17	87,61	67,52	31,12	2,12	0,00	0,00
	3.	96,60	99,55	92,92	60,48	32,63	13,89	0,86	0,00
	Avg	84,56	98,90	90,54	63,85	36,99	6,52	0,37	0,02
	SD	10,44	0,69	2,70	3,53	8,89	6,42	0,44	0,04
dU-10	1.	7,41	99,90	98,52	84,02	38,73	1,04	0,29	0,05
	2.	11,37	94,93	90,46	81,81	59,23	6,01	0,00	0,00
	3.	31,50	99,54	95,39	78,19	57,68	19,45	0,69	0,40
	Avg	16,76	98,12	94,79	81,34	51,88	8,83	0,33	0,15
	SD	12,92	2,77	4,07	2,94	11,41	9,53	0,35	0,22
dU+1	1.	0,00	42,86	77,84	82,28	80,88	79,36	89,50	71,32
	2.	0,22	22,79	72,33	87,74	89,77	79,12	76,80	75,66
	3.	0,01	45,16	81,26	84,24	83,16	80,99	81,83	79,75
	Avg	0,08	36,94	77,14	84,76	84,61	79,83	82,71	75,58
	SD	0,13	12,30	4,50	2,76	4,62	1,02	6,40	4,21
dU+4	1.	0,00	33,82	66,29	77,83	65,25	45,82	35,27	24,11
	2.	0,17	11,09	63,30	76,23	70,14	54,04	41,38	29,25
	3.	0,09	36,42	86,54	86,71	65,68	62,57	48,37	41,65
	Avg	0,08	27,11	72,04	80,25	67,02	54,14	41,67	31,67
	SD	0,08	13,94	12,64	5,65	2,71	8,37	6,55	9,02
dU+7	1.	3,05	91,88	80,05	63,12	39,16	13,35	4,79	1,70
	2.	2,81	86,55	83,88	72,11	49,61	23,72	9,82	2,53
	3.	5,29	82,60	70,13	64,98	46,96	15,44	8,53	4,24
	Avg	3,72	87,01	78,02	66,73	45,24	17,50	7,71	2,82
	SD	1,37	4,66	7,10	4,75	5,43	5,48	2,61	1,30
dU+10	1.	2,41	74,63	63,40	33,78	11,37	5,10	1,51	0,57
	2.	1,46	72,57	66,02	36,62	19,20	10,85	5,44	6,28
	3.	0,71	81,07	65,48	31,69	11,24	6,83	1,91	0,31
	Avg	1,53	76,09	64,97	34,03	13,94	7,59	2,95	2,39
	SD	0,85	4,43	1,38	2,47	4,56	2,95	2,16	3,37

Table S9. Polymerase activity - ScdG. Raw numerical data of densitometry obtained from Quantity One software.

ScdG		Time [min]							
		0	1	5	15	30	60	90	120
Strand	Data set	Polymerase activity [%]							
dU0	1.	1,61	1,67	8,67	3,02	2,52	2,01	2,36	3,72
	2.	0,47	0,76	1,93	3,00	3,20	2,71	2,97	3,11
	3.	0,16	1,40	2,71	4,64	5,29	5,98	6,06	4,29
	Avg	0,75	1,28	4,44	3,55	3,67	3,56	3,80	3,70
	SD	0,76	0,47	3,69	0,94	1,44	2,12	1,99	0,59
dU-1	1.	0,04	0,10	1,75	21,98	47,45	48,91	35,24	25,36
	2.	0,05	0,16	1,56	23,10	41,06	40,96	37,14	20,23
	3.	0,02	0,18	7,82	31,96	44,86	49,57	49,06	43,46
	Avg	0,04	0,14	3,71	25,68	44,45	46,48	40,48	29,69
	SD	0,02	0,04	3,56	5,47	3,21	4,79	7,49	12,20
dU-4	1.	0,23	2,64	19,33	45,53	48,61	32,96	23,24	18,45
	2.	3,96	7,92	27,50	40,97	48,14	34,49	32,11	18,54
	3.	0,07	2,46	28,08	27,77	41,33	37,32	26,58	28,30
	Avg	1,42	4,34	24,97	38,09	46,02	34,92	27,31	21,76
	SD	2,20	3,11	4,89	9,23	4,08	2,21	4,48	5,66
dU-7	1.	0,00	0,22	7,96	19,99	5,71	11,40	4,36	1,81
	2.	0,00	0,00	9,29	20,23	22,44	4,15	0,00	0,00
	3.	0,00	0,11	3,78	24,65	32,57	31,99	18,52	5,83
	Avg	0,00	0,11	7,01	21,62	20,24	15,85	7,63	2,55
	SD	0,00	0,11	2,88	2,62	13,56	14,44	9,68	2,98
dU-10	1.	0,02	0,06	1,46	13,46	30,45	22,29	17,40	13,20
	2.	0,00	0,00	3,84	12,31	26,19	20,49	12,57	3,92
	3.	0,07	0,05	4,30	18,08	23,23	18,15	9,04	4,66
	Avg	0,03	0,03	3,20	14,62	26,62	20,31	13,00	7,26
	SD	0,04	0,03	1,52	3,06	3,63	2,07	4,20	5,16
dU+1	1.	0,77	0,62	1,47	3,18	3,05	3,35	1,24	5,44
	2.	1,13	0,61	2,42	2,30	2,51	3,80	4,77	4,65
	3.	0,00	0,21	3,68	4,37	1,62	0,85	0,63	0,66
	Avg	0,63	0,48	2,52	3,28	2,40	2,67	2,21	3,58
	SD	0,58	0,23	1,11	1,04	0,72	1,59	2,23	2,56
dU+4	1.	0,00	0,60	3,35	11,83	25,92	35,80	46,99	45,10
	2.	0,80	0,98	5,53	14,78	20,46	26,73	40,98	48,80
	3.	0,19	0,19	2,25	7,04	9,86	24,39	27,25	32,76
	Avg	0,33	0,59	3,71	11,22	18,75	28,98	38,41	42,22
	SD	0,42	0,39	1,67	3,91	8,16	6,03	10,12	8,40
dU+7	1.	0,83	4,72	16,79	29,92	34,06	27,56	19,31	9,86
	2.	1,07	4,18	8,57	23,02	32,62	25,42	14,83	6,60
	3.	0,42	5,69	16,63	25,99	33,05	21,43	18,70	8,28
	Avg	0,77	4,86	14,00	26,31	33,24	24,80	17,61	8,25
	SD	0,33	0,76	4,70	3,46	0,74	3,11	2,43	1,63
dU+10	1.	3,31	10,12	29,30	65,39	83,21	82,05	77,67	73,71
	2.	2,47	8,69	27,07	61,09	78,19	76,68	74,75	59,38
	3.	0,84	5,63	27,58	67,89	87,41	77,39	72,27	61,67
	Avg	2,21	8,15	27,98	64,79	82,94	78,71	74,90	64,92
	SD	1,25	2,29	1,17	3,44	4,61	2,92	2,70	7,70

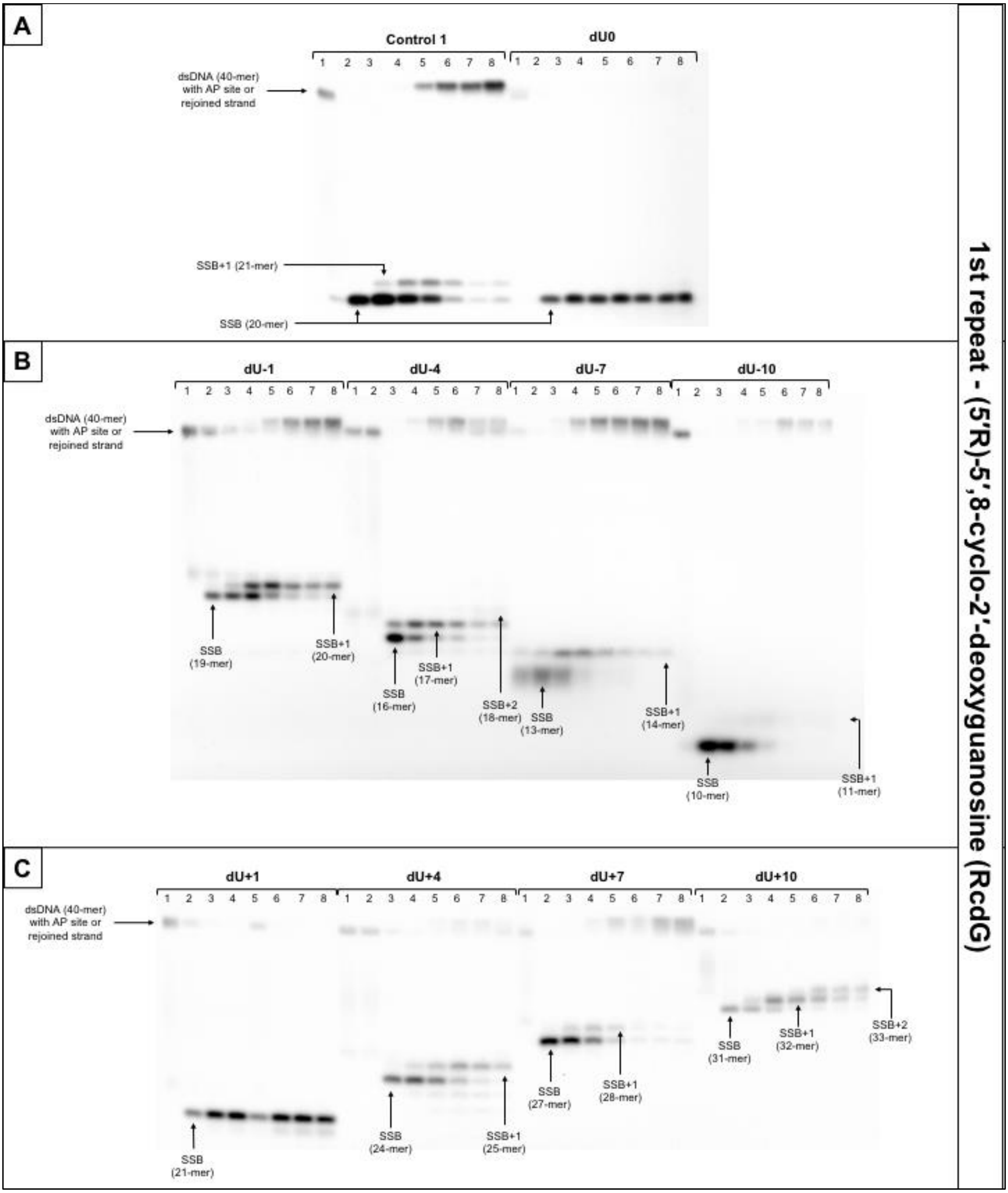
A
Control 1
dU0
1 2 3 4 5 6 7 8
dsDNA (40-mer) with AP site or rejoined strand
SSB+1 (21-mer)
SSB (20-mer)
B
dU-1
dU-4
dU-7
dU-10
SSB (19-mer)
SSB+1 (20-mer)
SSB (16-mer)
SSB+1 (17-mer)
SSB+2 (18-mer)
SSB (13-mer)
SSB+1 (14-mer)
SSB (10-mer)
SSB+1 (11-mer)
C
dU+1
dU+4
dU+7
dU+10
SSB (21-mer)
SSB (24-mer)
SSB+1 (25-mer)
SSB (27-mer)
SSB+1 (28-mer)
SSB (31-mer)
SSB+1 (32-mer)
SSB+2 (33-mer)
1st repeat - (5'R)-5',8-cyclo-2'-deoxyguanosine (RcdG)

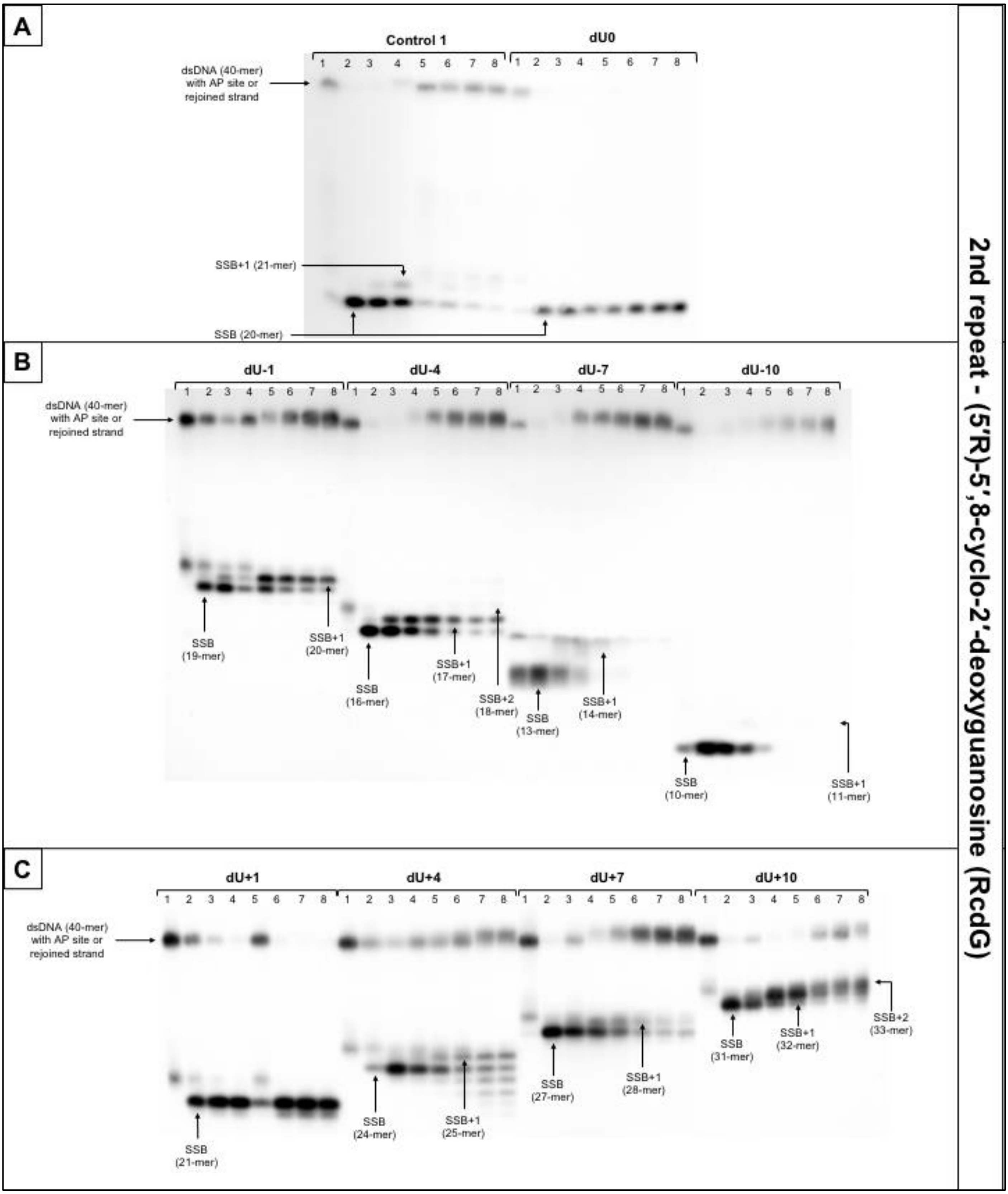
A
Control 1
dU0
dsDNA (40-mer) with AP site or rejoined strand
SSB+1 (21-mer)
SSB (20-mer)
B
dU-1
dU-4
dU-7
dU-10
dsDNA (40-mer) with AP site or rejoined strand
SSB (19-mer)
SSB+1 (20-mer)
SSB (16-mer)
SSB+1 (17-mer)
SSB+2 (18-mer)
SSB (13-mer)
SSB+1 (14-mer)
SSB (10-mer)
SSB+1 (11-mer)
C
dU+1
dU+4
dU+7
dU+10
dsDNA (40-mer) with AP site or rejoined strand
SSB (21-mer)
SSB (24-mer)
SSB+1 (25-mer)
SSB (27-mer)
SSB+1 (28-mer)
SSB (31-mer)
SSB+1 (32-mer)
SSB+2 (33-mer)
2nd repeat - (5'R)-5',8-cyclo-2'-deoxyguanosine (RcdG)

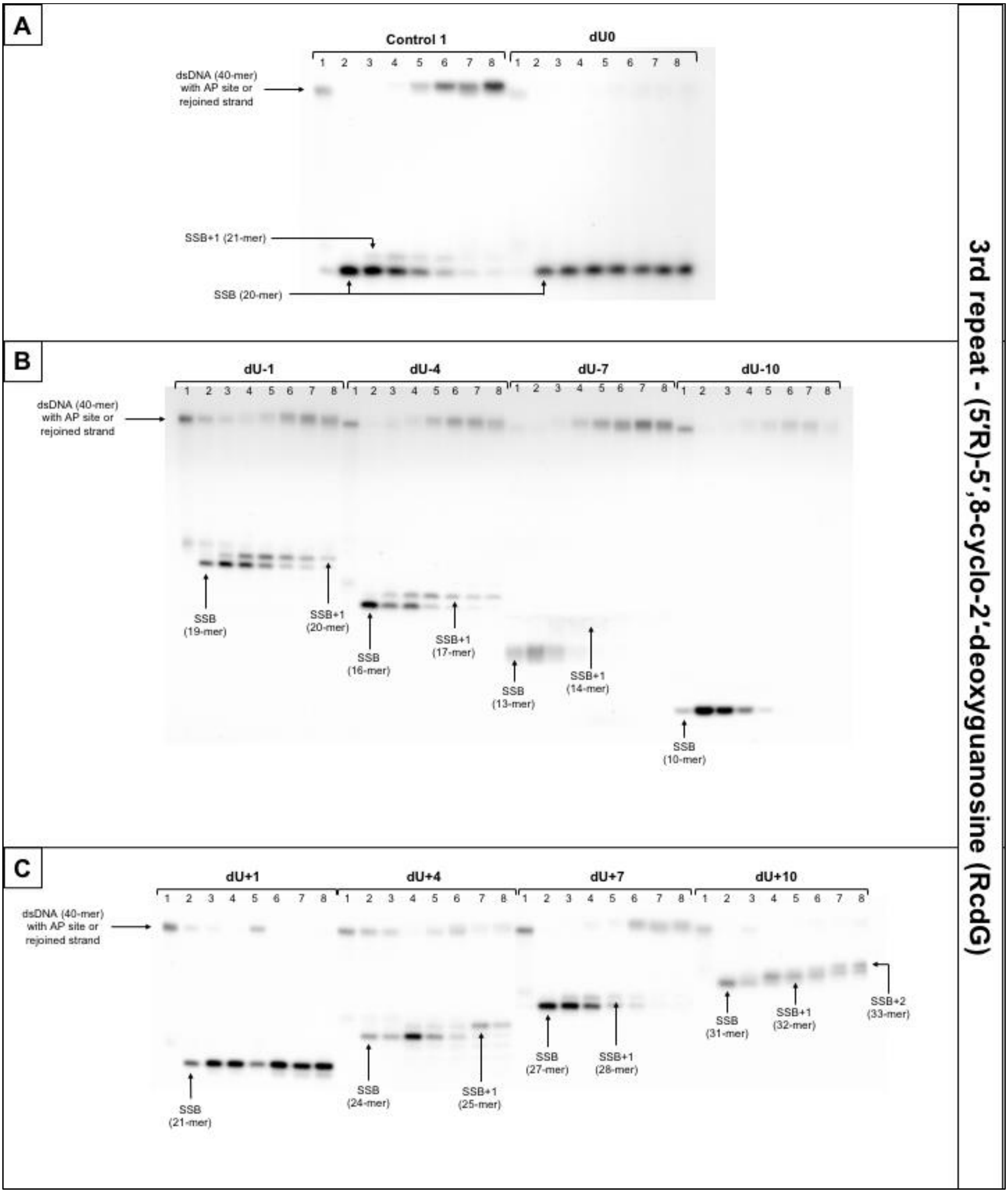

Figure S11. The autoradiograms of denaturing PAGE presenting repair of dsDNA containing clustered damage with AP site in one strand and RcdG in the opposing strand: (A) Controls: dsDNA with single lesion in one strand (Control 1); dsDNA with clustered lesions in two strands opposite to each other (dU0); (B) dsDNA with clustered lesions in two strands where AP site is located 1-10 base pairs in 3′ direction (negative numbers); (C) dsDNA with clustered lesions in two strands where AP site is located 1-10 base pairs in 5′ direction (positive numbers). Each lane corresponds with different assay time: lane 1 - 0 min; lane 2 - 1 min; lane 3 – 5 min; lane 4 – 15 min; lane 5 – 30 min; lane 6 – 60 min; lane 7 – 90 min; lane 8 – 120 min. Each replication of the experiment is shown.

A.

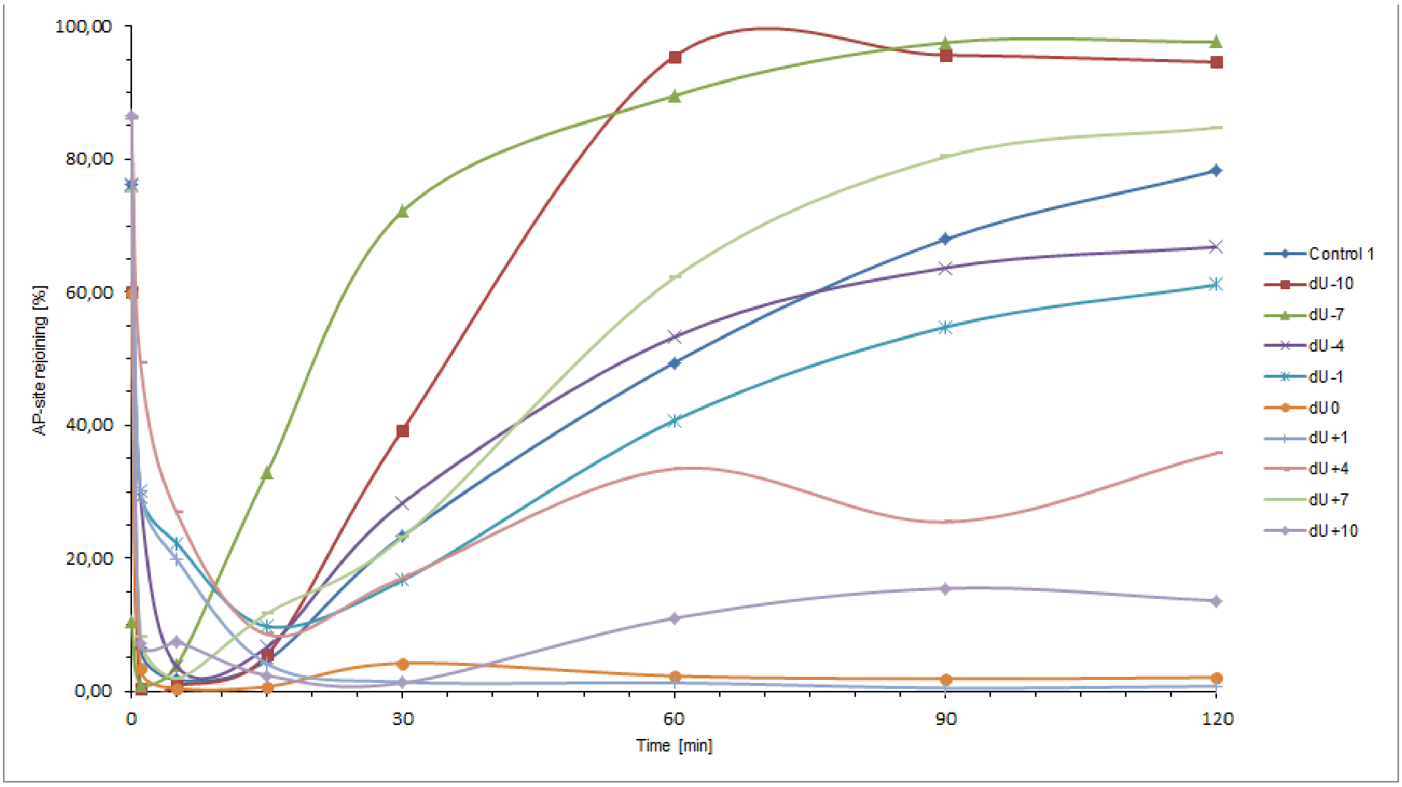

100,00
80,00
60,00
40,00
20,00
0,00
AP-site rejoining [%]
0
30
60
90
120
Time [min]
Control 1
dU-10
dU-7
dU-4
dU-1
dU0
dU+1
dU+4
dU+7
dU+10

B.

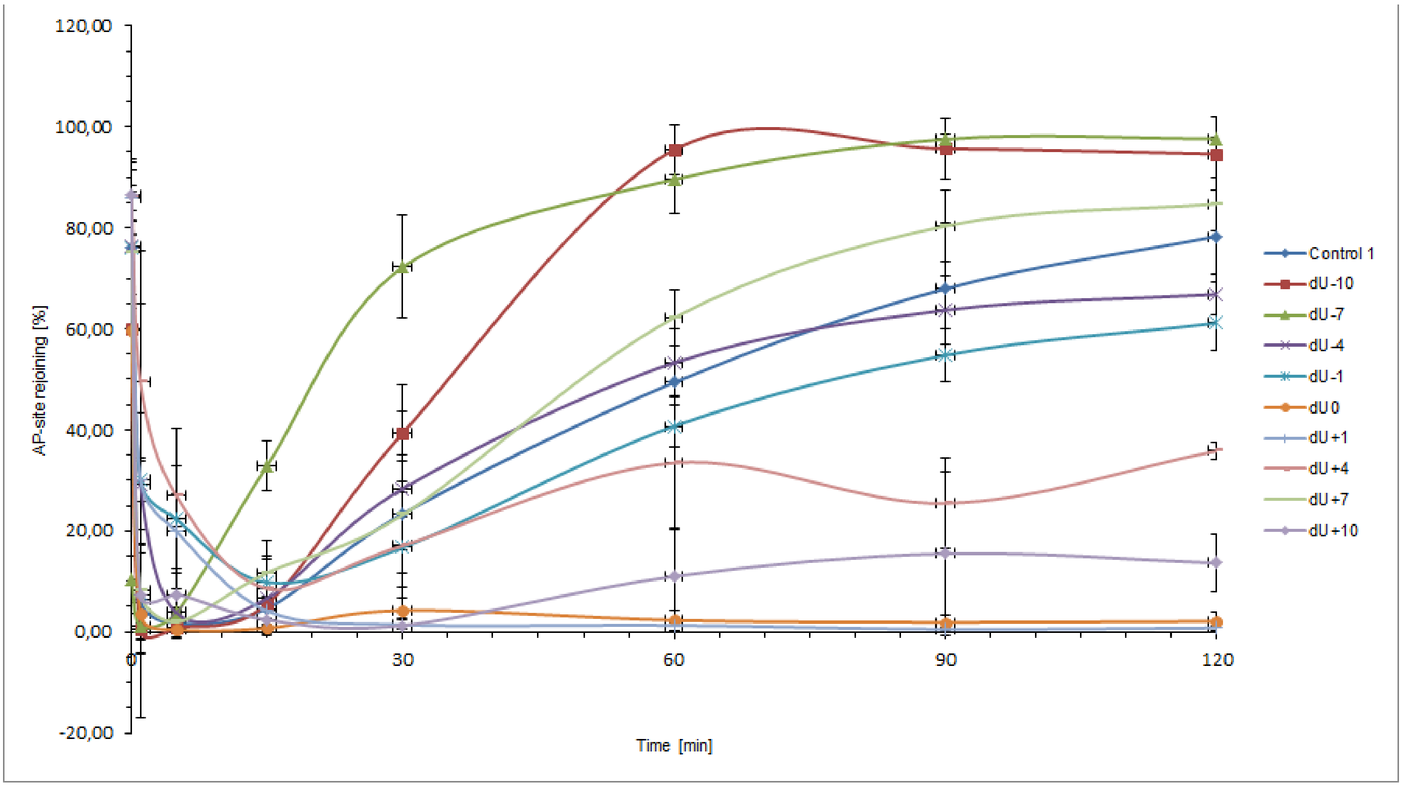

120,00
100,00
80,00
60,00
40,00
20,00
0,00
-20,00
AP-site rejoining [%]
0
30
60
90
120
Time [min]
Control 1
dU-10
dU-7
dU-4
dU-1
dU0
dU+1
dU+4
dU+7
dU+10

C.

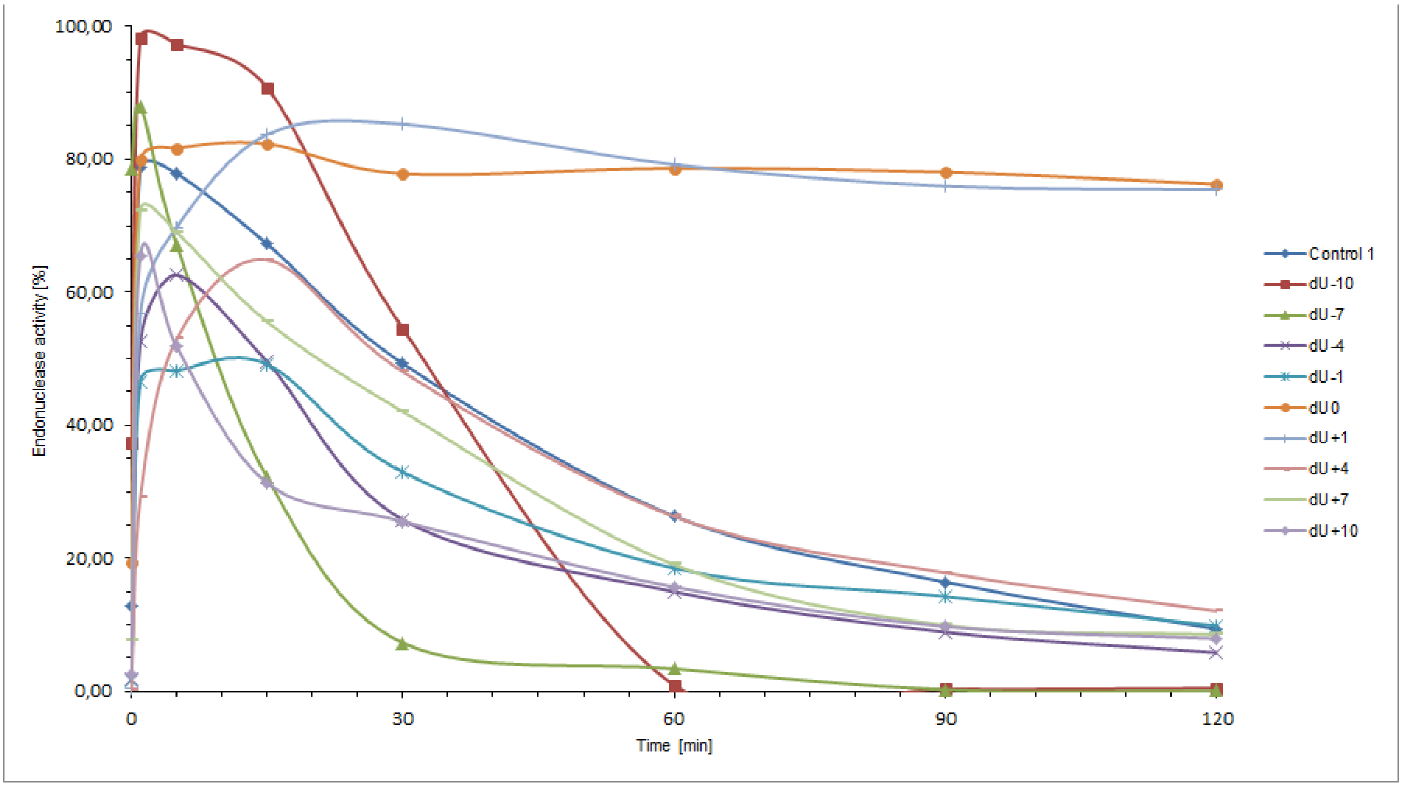

100,00
80,00
60,00
40,00
20,00
0,00
Endonuclease activity [%]
0
30
60
90
120
Time [min]
Control 1
dU-10
dU-7
dU-4
dU-1
dU0
dU+1
dU+4
dU+7
dU+10

D.

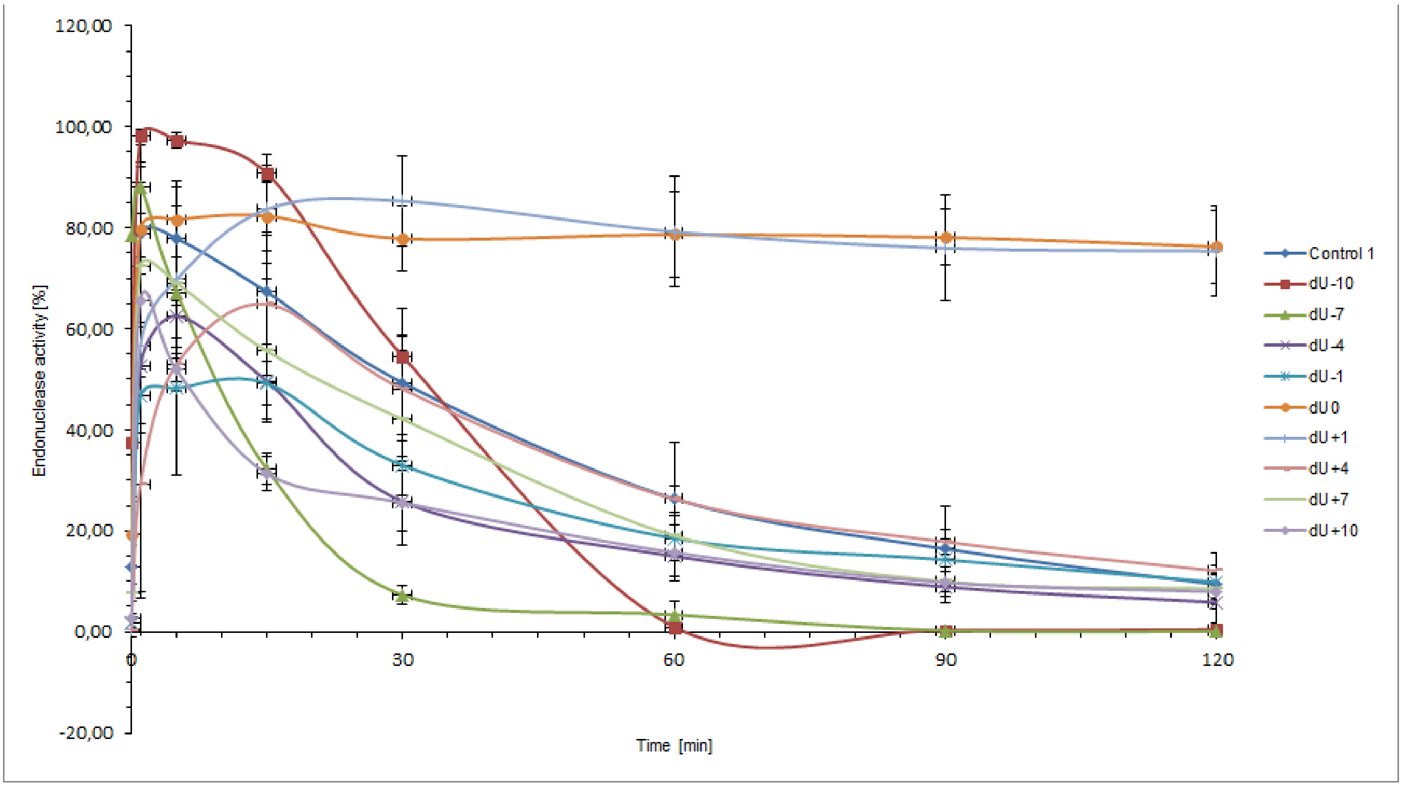

120,00
100,00
80,00
60,00
40,00
20,00
0,00
-20,00
Endonuclease activity [%]
0
30
60
90
120
Time [min]
Control 1
dU-10
dU-7
dU-4
dU-1
dU0
dU+1
dU+4
dU+7
dU+10

E.

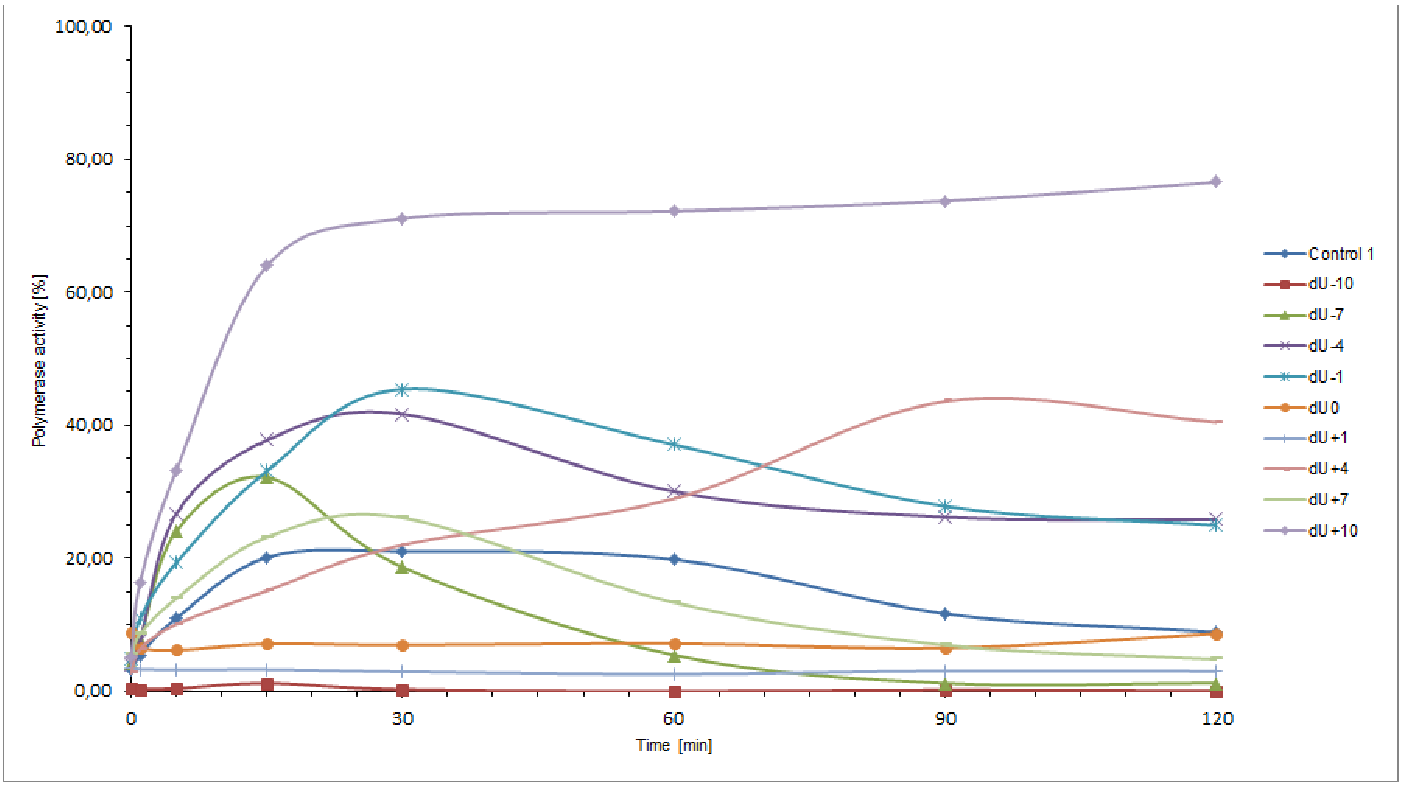

F.

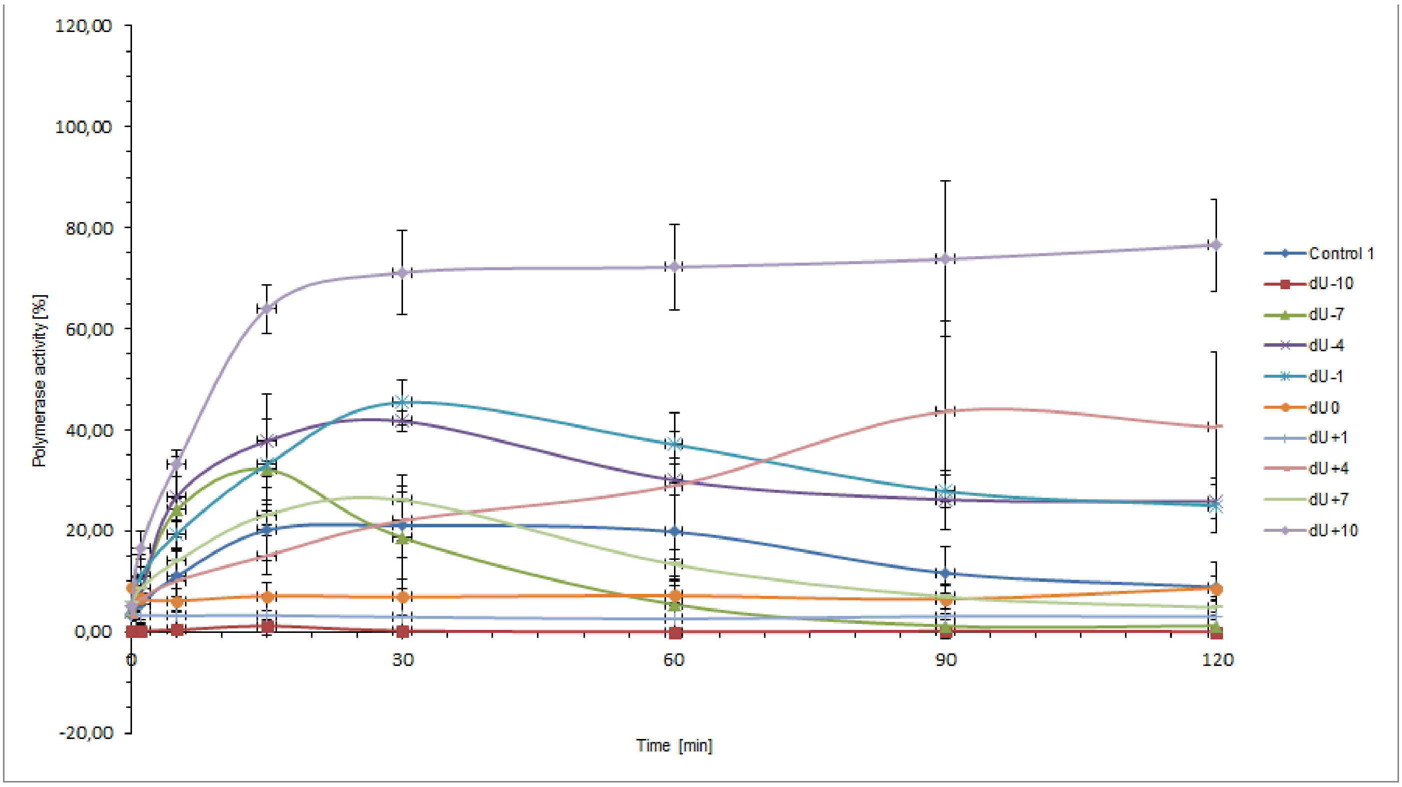

Figure S12. Graphical representation of DNA repair assays' results for RcdG. (A) AP site rejoining efficiency, (B) AP site rejoining efficiency + SD, (C) endonuclease activity, (D) endonuclease activity + SD, (E) polymerase activity, (F) polymerase activity + SD

Table S10. AP site rejoining - RcdG. Raw numerical data of densitometry obtained from Quantity One software.

RcdG		Time [min]							
		0	1	5	15	30	60	90	120
Strand	Data set	AP site rejoining [%]							
dU0	1.	81,24	0,80	0,23	0,31	0,45	1,00	0,99	0,87
	2.	60,13	9,23	1,10	0,70	9,14	1,37	1,08	1,30
	3.	37,91	0,38	0,15	1,13	3,11	4,56	3,63	4,13
	Avg	59,76	3,47	0,49	0,72	4,23	2,31	1,90	2,10
	SD	21,67	4,99	0,52	0,41	4,46	1,96	1,50	1,77
dU-1	1.	82,93	34,35	13,74	5,92	17,09	45,13	59,29	62,66
	2.	63,29	30,58	43,10	15,88	16,83	36,76	49,12	55,02
	3.	81,80	25,55	10,04	7,54	16,21	40,28	55,90	65,72
	Avg	76,01	30,16	22,29	9,78	16,71	40,72	54,77	61,13
	SD	11,03	4,41	18,11	5,35	0,45	4,20	5,18	5,51
dU-4	1.	81,10	82,66	1,06	5,05	26,44	49,95	59,06	65,39
	2.	62,37	3,45	1,09	5,75	23,96	48,94	60,59	63,83
	3.	85,26	1,78	9,15	9,12	34,65	60,93	71,39	71,26
	Avg	76,24	29,30	3,77	6,64	28,35	53,27	63,68	66,83
	SD	12,19	46,22	4,66	2,18	5,60	6,65	6,72	3,92
dU-7	1.	11,83	0,90	3,62	34,64	80,58	93,53	97,50	97,54
	2.	14,27	1,75	3,15	27,20	60,85	81,77	96,57	97,38
	3.	5,29	0,38	4,71	36,80	75,17	93,30	98,54	98,00
	Avg	10,46	1,01	3,82	32,88	72,20	89,53	97,53	97,64
	SD	4,64	0,69	0,80	5,04	10,20	6,72	0,99	0,32
dU-10	1.	81,28	0,82	0,31	4,92	28,51	89,82	88,69	86,37
	2.	48,11	0,32	2,27	6,44	45,70	98,96	99,19	99,69
	3.	51,00	0,00	0,00	5,20	43,85	97,48	99,11	97,73
	Avg	60,13	0,38	0,86	5,52	39,35	95,42	95,66	94,59
	SD	18,37	0,41	1,23	0,81	9,43	4,90	6,04	7,19
dU+1	1.	87,98	18,67	19,01	2,46	1,43	1,53	0,42	0,63
	2.	78,09	44,58	27,73	7,40	2,35	2,05	0,84	1,34
	3.	91,87	27,46	12,88	2,60	0,40	0,26	0,20	0,34
	Avg	85,98	30,24	19,87	4,15	1,39	1,28	0,49	0,77
	SD	7,11	13,18	7,46	2,81	0,98	0,92	0,33	0,51
dU+4	1.	88,05	67,30	29,33	8,62	6,00	19,68	26,60	35,72
	2.	80,53	42,61	20,03	14,40	26,68	35,50	33,79	37,57
	3.	90,33	38,37	31,29	2,63	18,60	45,21	15,98	34,19
	Avg	86,30	49,43	26,88	8,55	17,10	33,46	25,46	35,83
	SD	5,13	15,62	6,02	5,89	10,42	12,88	8,96	1,69
dU+7	1.	66,47	1,71	1,63	14,28	46,92	66,32	83,95	87,07
	2.	75,62	21,98	3,63	16,41	12,77	55,91	72,14	78,76
	3.	83,11	0,52	0,49	4,44	9,83	64,29	84,96	88,40
	Avg	75,07	8,07	1,92	11,71	23,17	62,17	80,35	84,74
	SD	8,34	12,06	1,59	6,39	20,62	5,52	7,13	5,22
dU+10	1.	90,51	16,92	7,14	0,69	1,59	7,51	9,33	13,91
	2.	80,67	2,63	3,56	6,01	1,22	21,43	33,77	19,28
	3.	88,04	2,04	11,62	0,47	1,02	4,06	3,33	7,69
	Avg	86,41	7,20	7,44	2,39	1,28	11,00	15,48	13,62
	SD	5,12	8,43	4,03	3,13	0,29	9,20	16,12	5,80

Table S11. Endonuclease activity - RcdG. Raw numerical data of densitometry obtained from Quantity One software.

RcdG		Time [min]							
		0	1	5	15	30	60	90	120
Strand	Data set	Endonuclease activity [%]							
dU0	1.	12,45	90,35	88,16	87,35	84,47	83,41	80,26	76,84
	2.	21,45	75,43	83,43	85,08	77,44	83,82	82,16	83,22
	3.	24,38	73,76	73,41	74,53	71,61	68,74	71,80	68,67
	Avg	19,43	79,85	81,67	82,32	77,84	78,66	78,07	76,24
	SD	6,21	9,14	7,53	6,84	6,44	8,59	5,52	7,29
dU-1	1.	1,27	46,12	54,57	44,26	26,11	13,67	9,87	7,18
	2.	2,63	41,42	28,67	51,34	36,95	22,07	18,02	13,46
	3.	0,95	52,25	61,42	51,93	35,89	19,82	14,92	8,95
	Avg	1,62	46,60	48,22	49,18	32,98	18,52	14,27	9,86
	SD	0,89	5,43	17,27	4,27	5,98	4,35	4,11	3,23
dU-4	1.	0,00	0,00	69,09	43,47	23,12	17,28	8,00	3,75
	2.	3,67	72,13	56,64	47,55	32,67	17,38	12,32	10,50
	3.	1,63	85,57	62,00	57,75	21,60	10,29	6,37	3,25
	Avg	1,77	52,57	62,58	49,59	25,80	14,98	8,89	5,83
	SD	1,84	46,02	6,25	7,36	6,00	4,07	3,07	4,05
dU-7	1.	82,53	91,12	66,61	30,15	6,41	2,98	0,00	0,00
	2.	66,70	82,12	64,90	35,77	9,56	6,29	0,74	0,24
	3.	86,44	90,61	70,07	30,57	5,97	0,92	0,00	0,00
	Avg	78,56	87,95	67,19	32,16	7,31	3,40	0,25	0,08
	SD	10,45	5,06	2,64	3,13	1,96	2,71	0,42	0,14
dU-10	1.	14,13	97,14	97,41	89,86	55,03	0,00	0,00	0,00
	2.	49,76	98,18	95,67	89,71	53,19	0,55	0,11	0,00
	3.	48,58	99,31	98,87	92,69	55,43	2,36	0,88	1,38
	Avg	37,49	98,21	97,32	90,75	54,55	0,97	0,33	0,46
	SD	20,24	1,08	1,60	1,68	1,19	1,24	0,48	0,80
dU+1	1.	0,00	68,41	73,05	89,87	88,38	84,06	81,50	80,16
	2.	0,50	36,64	53,72	71,16	75,18	66,65	63,84	65,11
	3.	0,62	65,03	82,55	90,09	92,29	86,90	82,54	80,93
	Avg	0,37	56,69	69,77	83,70	85,29	79,20	75,96	75,40
	SD	0,33	17,45	14,69	10,87	8,96	10,96	10,51	8,92
dU+4	1.	0,00	6,11	58,89	68,09	53,59	27,43	15,49	8,47
	2.	0,34	33,60	51,44	49,44	36,14	23,27	20,28	15,54
	3.	0,38	48,04	48,57	77,07	54,45	28,17	17,67	12,23
	Avg	0,24	29,25	52,97	64,87	48,06	26,29	17,81	12,08
	SD	0,21	21,30	5,32	14,09	10,33	2,64	2,39	3,54
dU+7	1.	9,63	78,59	70,60	51,84	25,72	14,98	8,63	7,58
	2.	7,14	50,26	55,43	43,61	41,62	19,43	13,37	11,93
	3.	6,43	88,10	81,00	71,29	58,86	22,70	7,81	6,10
	Avg	7,73	72,32	69,01	55,58	42,07	19,04	9,94	8,53
	SD	1,68	19,68	12,86	14,21	16,57	3,88	3,00	3,03
dU+10	1.	2,23	62,23	50,77	27,63	17,15	10,91	9,64	8,10
	2.	2,93	62,71	54,43	32,53	25,63	14,35	9,25	11,24
	3.	2,57	71,56	50,32	33,84	33,87	21,85	10,35	4,49
	Avg	2,58	65,50	51,84	31,34	25,55	15,70	9,74	7,94
	SD	0,35	5,26	2,25	3,27	8,36	5,60	0,56	3,38

Table S12. Polymerase activity - RcdG. Raw numerical data of densitometry obtained from Quantity One software.

RcdG		Time [min]							
		0	1	5	15	30	60	90	120
Strand	Data set	Polymerase activity [%]							
dU0	1.	2,23	2,00	3,72	4,77	4,17	6,41	5,03	10,14
	2.	9,10	7,49	6,53	6,41	5,75	5,60	4,79	5,93
	3.	15,33	10,31	8,31	10,12	10,93	9,52	9,66	9,93
	Avg	8,89	6,60	6,19	7,10	6,95	7,18	6,49	8,67
	SD	6,55	4,22	2,31	2,74	3,54	2,07	2,74	2,37
dU-1	1.	1,37	8,44	22,44	41,74	50,44	37,08	27,24	25,80
	2.	9,82	15,01	17,87	23,62	44,10	39,64	31,29	29,82
	3.	3,31	9,32	17,68	33,91	41,78	34,55	24,91	19,30
	Avg	4,84	10,92	19,33	33,09	45,44	37,09	27,81	24,97
	SD	4,42	3,57	2,70	9,09	4,49	2,55	3,23	5,31
dU-4	1.	0,00	0,00	23,18	44,05	44,06	31,24	32,27	29,68
	2.	7,96	15,23	35,77	42,17	40,81	32,31	25,73	24,29
	3.	3,66	7,68	21,18	27,05	40,19	26,55	20,54	23,58
	Avg	3,87	7,64	26,71	37,76	41,69	30,03	26,18	25,85
	SD	3,99	7,61	7,91	9,32	2,08	3,06	5,88	3,33
dU-7	1.	0,00	5,22	26,75	31,45	8,78	0,00	0,00	0,00
	2.	6,08	10,40	22,28	33,93	29,09	10,84	2,50	2,27
	3.	6,18	7,38	23,26	31,10	18,08	5,46	1,08	1,44
	Avg	4,09	7,67	24,10	32,16	18,65	5,43	1,19	1,24
	SD	3,54	2,60	2,35	1,54	10,17	5,42	1,26	1,15
dU-10	1.	0,00	0,00	0,00	0,00	0,00	0,00	0,00	0,00
	2.	0,62	0,35	0,47	1,99	0,70	0,00	0,47	0,12
	3.	0,35	0,40	0,70	1,54	0,00	0,00	0,00	0,00
	Avg	0,32	0,25	0,39	1,18	0,23	0,00	0,16	0,04
	SD	0,31	0,22	0,36	1,04	0,40	0,00	0,27	0,07
dU+1	1.	0,66	0,83	0,35	0,18	0,00	0,00	0,00	0,00
	2.	6,86	6,69	7,56	7,88	7,29	6,04	8,21	7,52
	3.	2,57	2,37	1,67	1,68	1,51	1,67	0,96	1,48
	Avg	3,36	3,30	3,20	3,25	2,93	2,57	3,06	3,00
	SD	3,17	3,04	3,84	4,08	3,85	3,12	4,49	3,99
dU+4	1.	0,06	1,99	5,20	13,33	25,55	44,77	50,84	53,00
	2.	6,55	11,49	16,61	19,64	22,59	25,93	23,16	24,19
	3.	1,49	5,60	8,38	12,35	17,83	16,17	56,79	44,26
	Avg	2,70	6,36	10,06	15,11	21,99	28,96	43,60	40,48
	SD	3,41	4,80	5,89	3,96	3,89	14,54	17,95	14,77
dU+7	1.	11,48	9,23	16,61	23,69	20,64	12,47	5,00	3,91
	2.	3,40	10,39	14,03	24,89	29,99	16,68	9,78	6,41
	3.	2,10	6,20	11,19	20,90	27,62	10,88	6,02	4,04
	Avg	5,66	8,60	13,94	23,16	26,08	13,34	6,93	4,78
	SD	5,08	2,16	2,71	2,05	4,86	2,99	2,52	1,41
dU+10	1.	7,00	12,43	36,08	68,64	79,09	80,27	79,80	75,80
	2.	1,73	19,51	32,87	59,10	71,44	63,57	56,22	67,89
	3.	6,41	17,35	30,78	64,18	62,73	72,76	85,25	86,08
	Avg	5,04	16,43	33,24	63,97	71,09	72,20	73,76	76,59
	SD	2,89	3,63	2,67	4,77	8,19	8,36	15,43	9,12

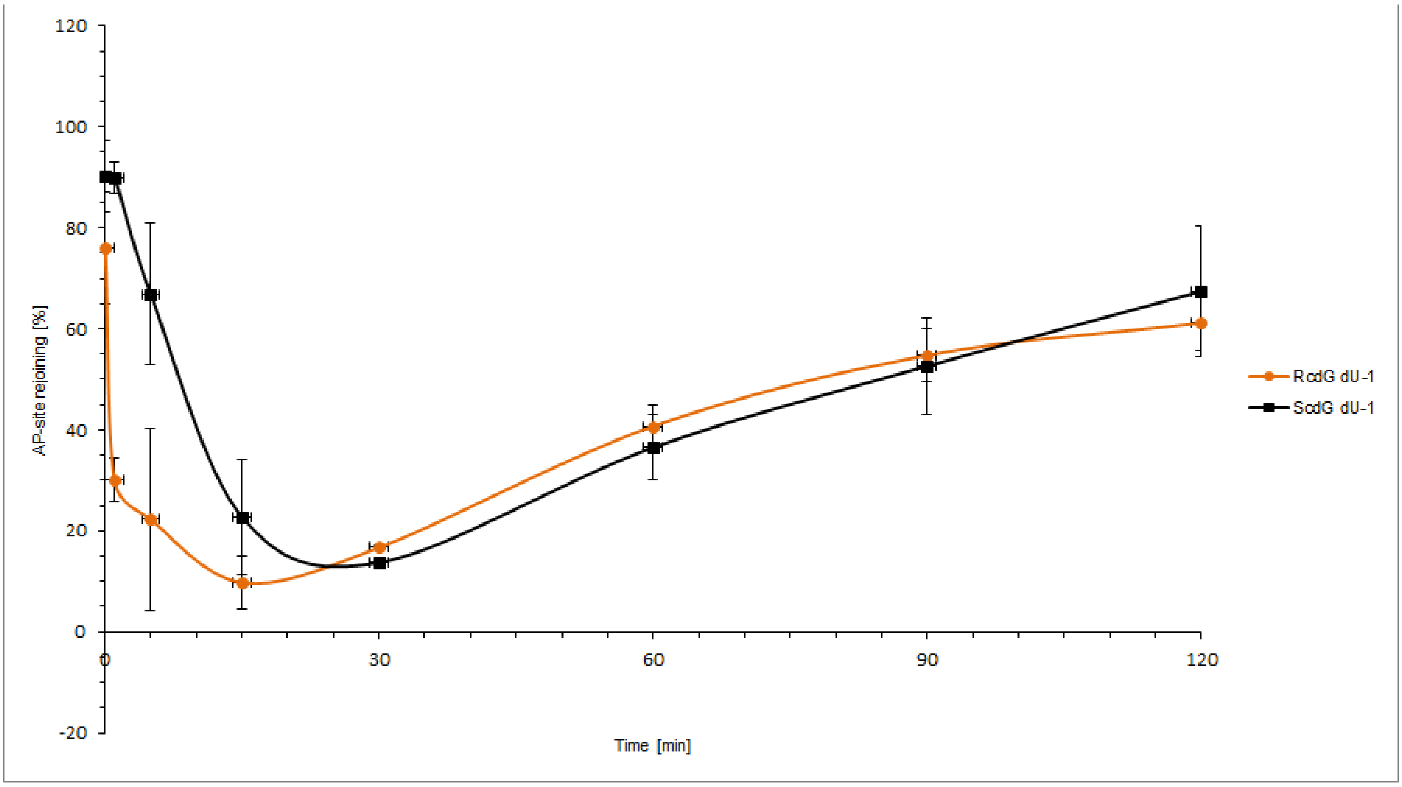

AP-site rejoining [%]
Time [min]
RcdG dU-1
ScdG dU-1

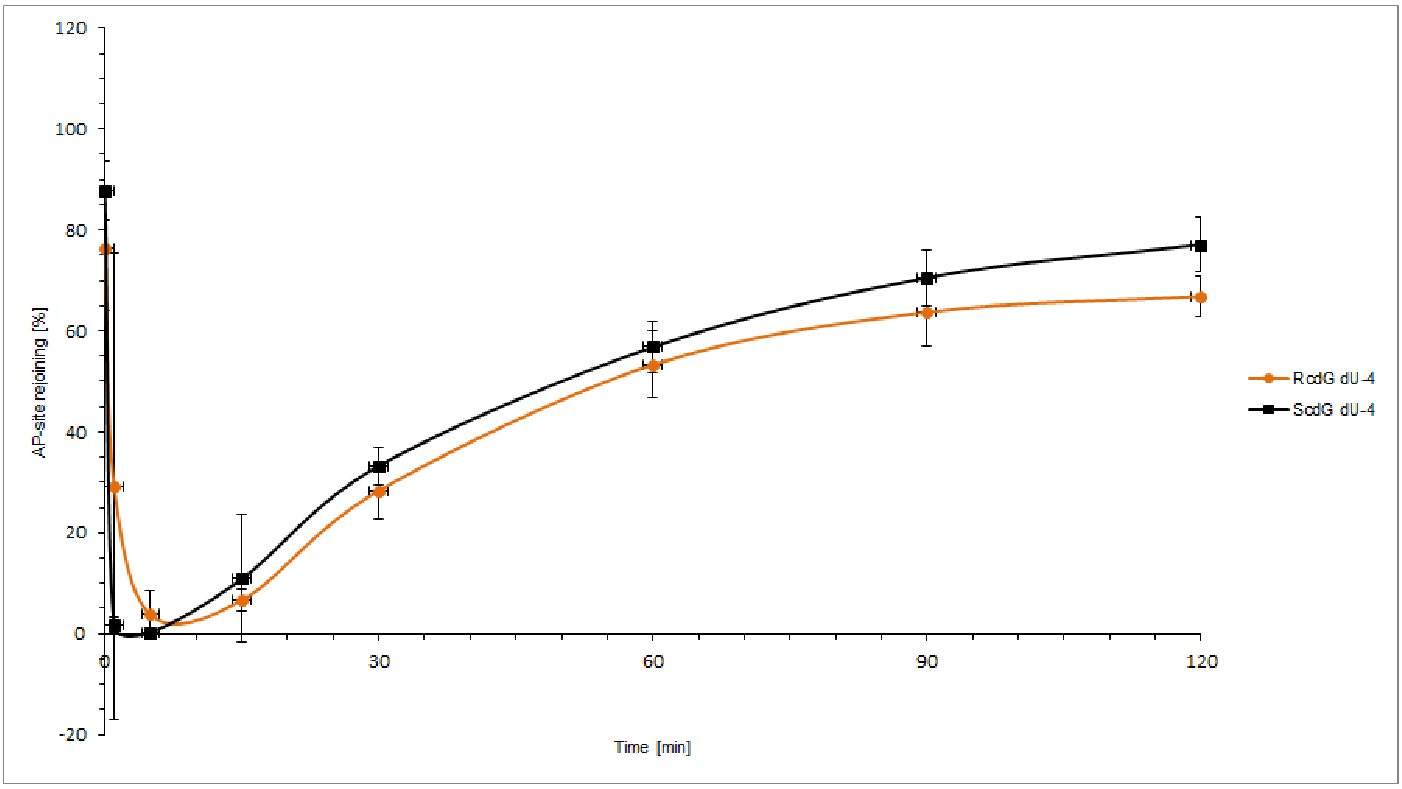

AP-site rejoining [%]
Time [min]
RcdG dU-4
ScdG dU-4

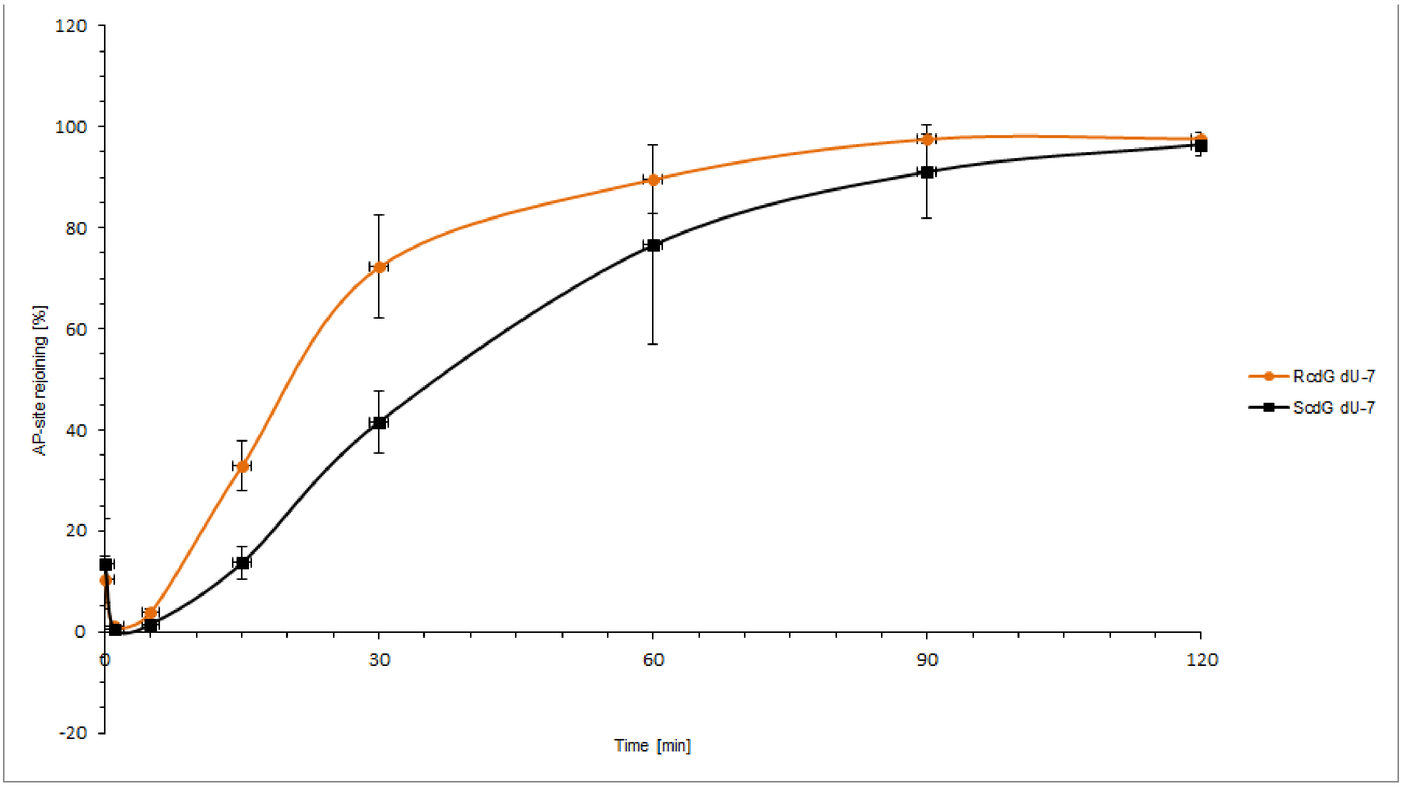
120
100
80
60
40
20
0
-20
AP-site rejoining [%]
0
30
60
90
120
Time [min]
RcdG dU-7
ScdG dU-7

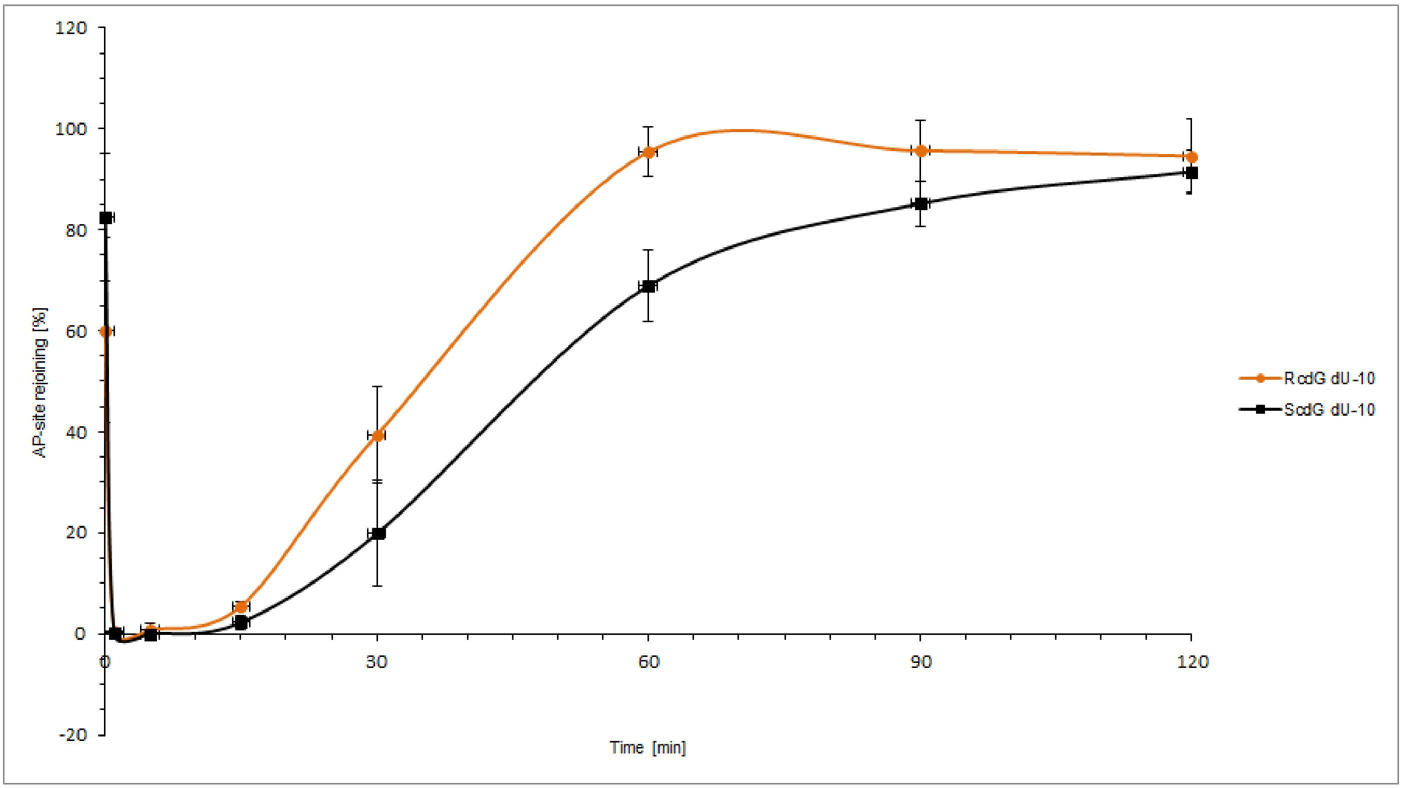
120
100
80
60
40
20
0
-20
AP-site rejoining [%]
0
30
60
90
120
Time [min]
RcdG dU-10
ScdG dU-10

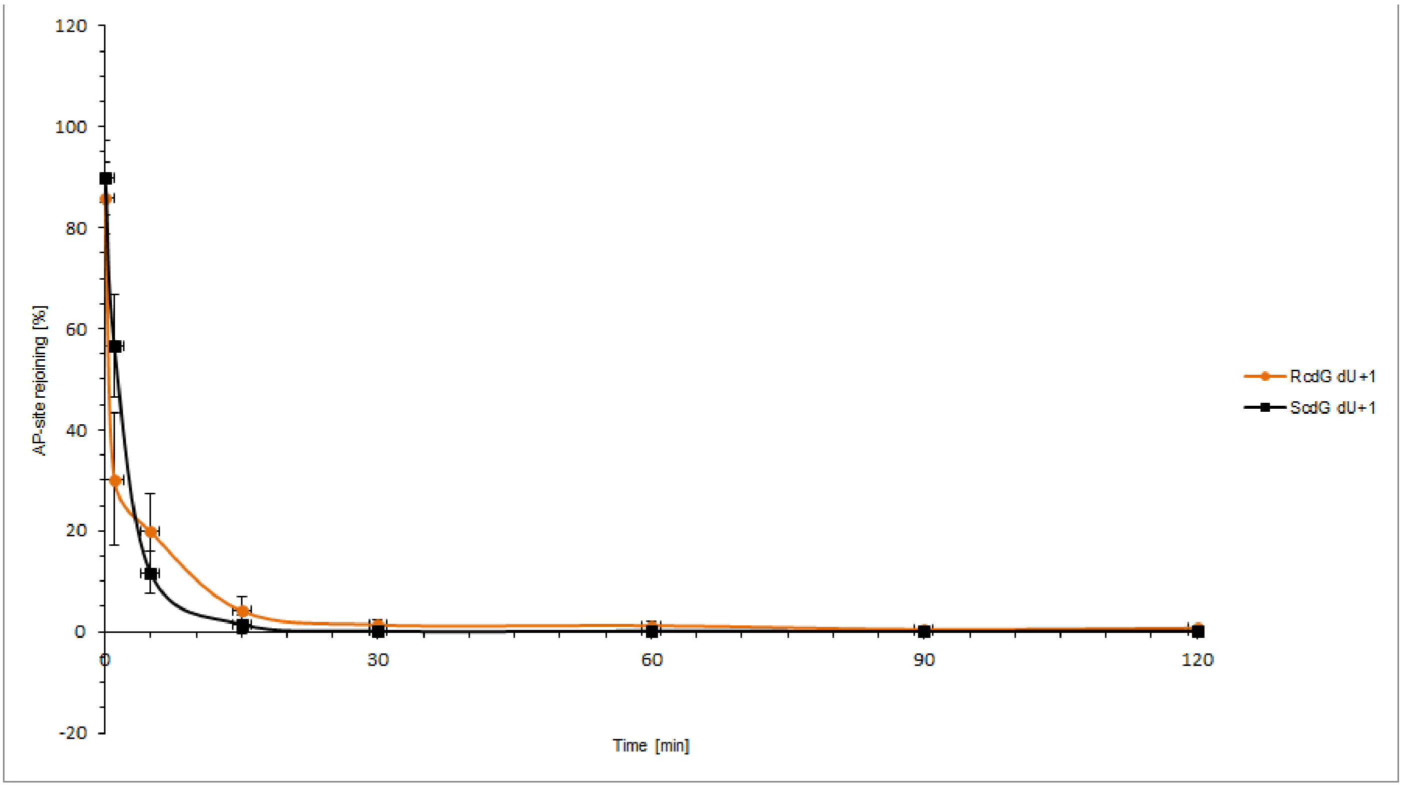

120
100
80
60
40
20
0
-20
AP-site rejoining [%]
0
30
60
90
120
Time [min]
RcdG dU+1
ScdG dU+1

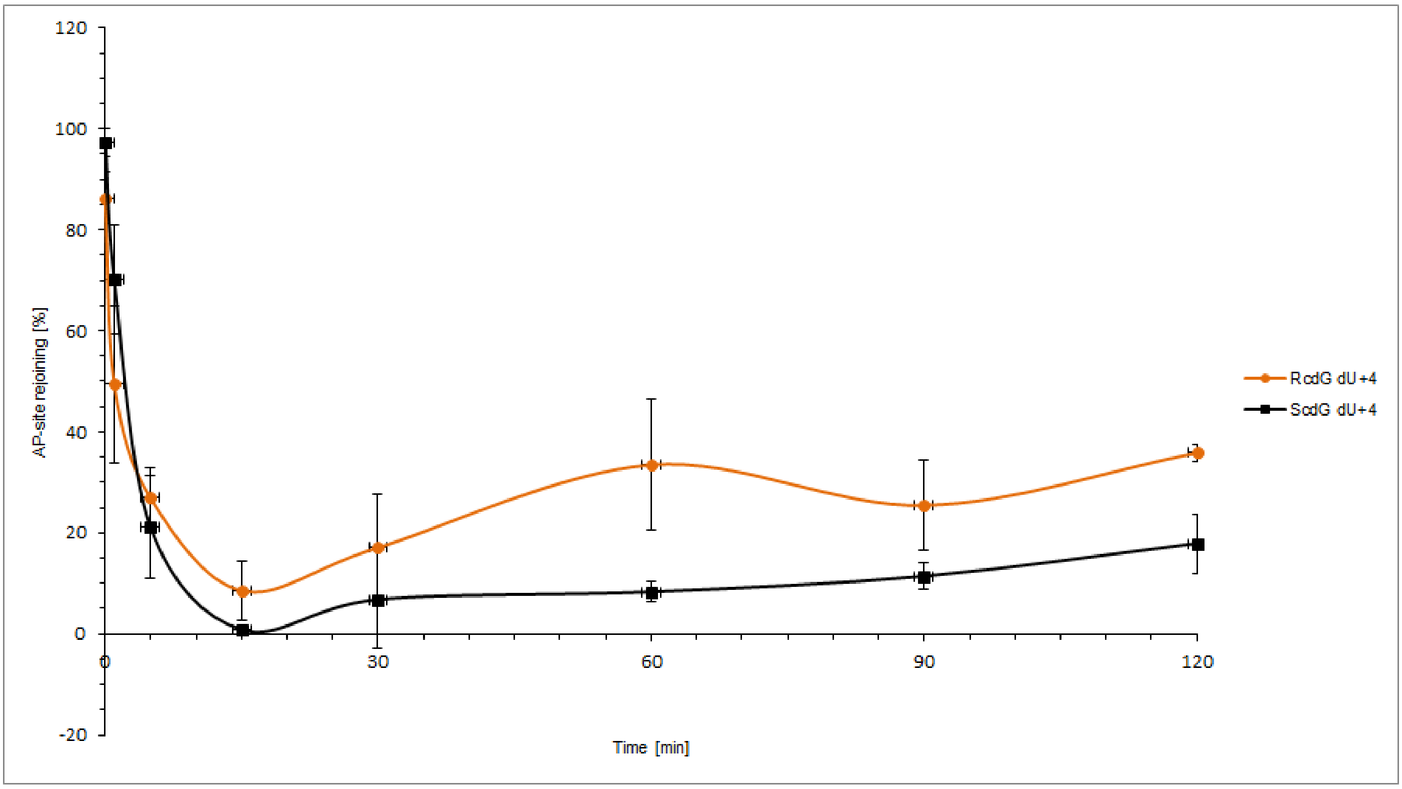

120
100
80
60
40
20
0
-20
AP-site rejoining [%]
0
30
60
90
120
Time [min]
RcdG dU+4
ScdG dU+4

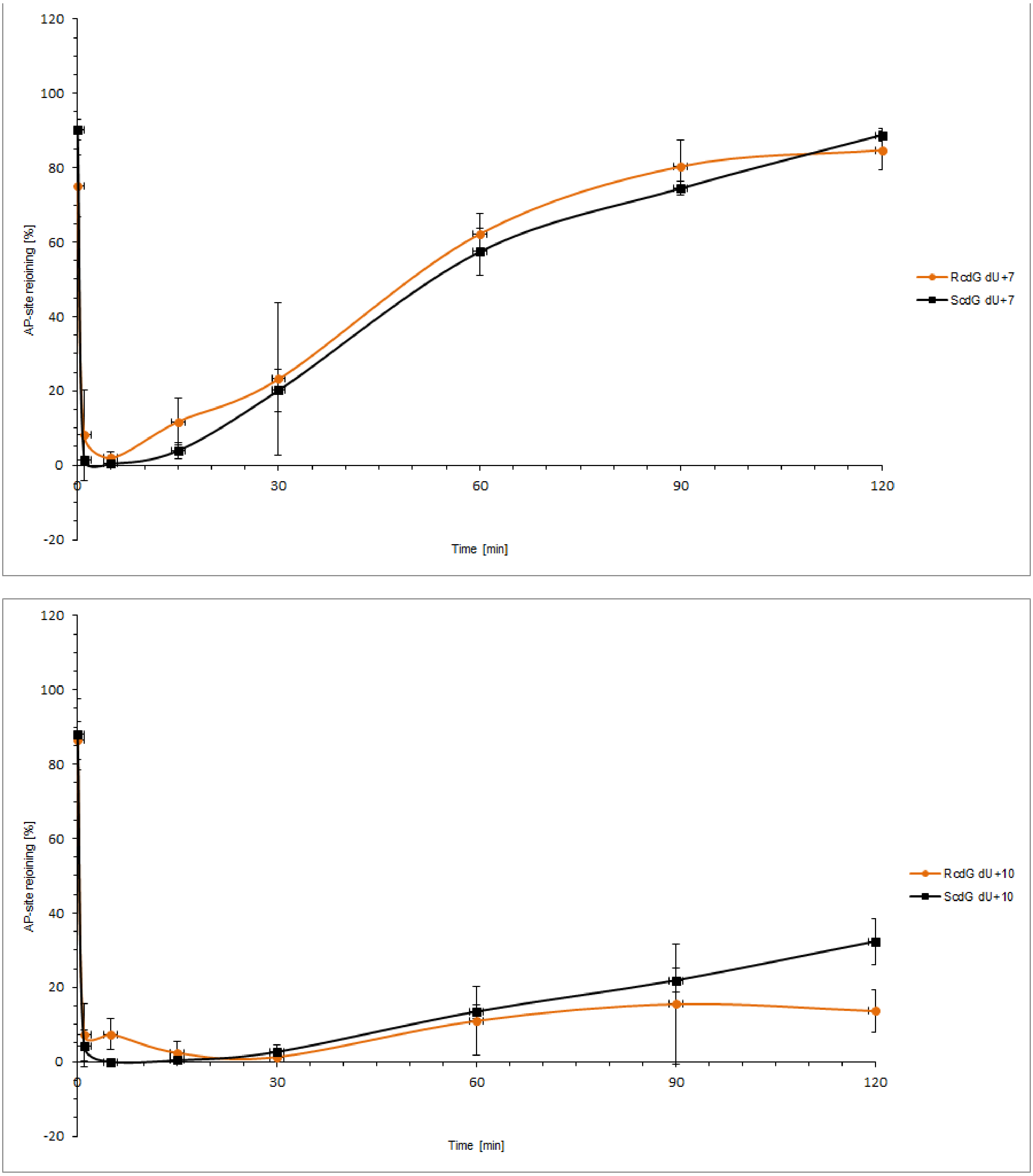

Figure S13. AP site rejoining [%] of ScdG vs. RcdG – comparison of individual strands

Table S13. AP site rejoining – Control 1. Raw numerical data of densitometry obtained from Quantity One software.

		Time [min]							
		0	1	5	15	30	60	90	120
Strand	Data set	AP site rejoining [%]							
Control 1	1.	61,63	0,39	0,17	2,08	12,89	41,61	59,58	75,83
	2.	85,45	3,35	0,78	5,33	27,84	45,55	72,31	83,88
	3.	98,97	0,30	0,10	0,63	12,11	53,11	69,45	80,91
	4.	86,90	32,00	8,23	14,58	29,67	57,96	61,26	61,84
	5.	89,90	25,74	7,63	12,52	38,16	35,94	65,84	74,31
	6.	66,90	11,62	0,21	0,48	4,14	18,51	35,18	69,33
	7.	81,69	0,53	0,39	5,19	24,59	58,50	70,37	79,70
	8.	89,72	0,21	0,05	1,48	21,77	54,82	76,64	86,67
	9.	82,58	0,08	0,10	0,65	15,51	54,49	78,58	88,67
	10.	76,65	0,09	0,04	0,69	19,89	63,55	88,44	87,04
	11.	38,96	3,51	2,81	9,91	46,43	46,53	62,68	64,57
	12.	54,31	0,31	0,14	2,35	27,50	62,23	74,98	86,94
	Avg	76,14	6,51	1,72	4,66	23,38	49,40	67,94	78,31
	SD	17,39	11,03	3,00	5,01	11,72	12,82	13,16	9,20

Table S14. Endonuclease activity – Control 1. Raw numerical data of densitometry obtained from Quantity One software.

		Time [min]							
		0	1	5	15	30	60	90	120
Strand	Data set	Endonuclease activity [%]							
Control 1	1.	24,92	87,92	79,31	73,74	66,79	41,08	26,32	14,68
	2.	6,42	63,54	64,70	52,16	36,40	18,65	10,96	5,46
	3.	0,02	86,20	84,61	76,48	58,31	23,49	15,64	8,16
	4.	5,28	49,73	64,54	51,54	34,38	15,32	12,59	11,85
	5.	3,64	52,90	63,11	51,47	25,43	14,69	9,87	6,84
	6.	0,49	61,64	76,55	72,47	67,63	51,70	38,08	11,39
	7.	3,87	88,67	79,70	60,18	44,08	18,44	12,34	11,68
	8.	2,02	94,11	82,69	75,76	52,12	23,97	15,47	7,23
	9.	12,40	99,41	95,39	82,52	65,54	34,19	17,27	8,22
	10.	20,41	96,74	88,63	76,85	56,34	20,28	7,04	8,19
	11.	39,02	74,02	71,44	60,21	31,85	30,94	18,00	12,40
	12.	36,41	89,23	83,42	73,94	52,89	23,55	13,66	6,39
	Avg	12,91	78,67	77,84	67,28	49,31	26,36	16,44	9,37
	SD	13,95	17,56	10,18	11,36	14,61	11,20	8,37	2,90

Table S15. Polymerase activity – Control 1. Raw numerical data of densitometry obtained from Quantity One software.

		Time [min]							
		0	1	5	15	30	60	90	120
Strand	Data set	Polymerase activity [%]							
Control 1	1.	6,26	3,96	8,73	13,32	12,51	12,42	10,77	7,36
	2.	3,22	11,94	21,86	32,12	28,34	33,68	15,50	9,82
	3.	0,15	2,63	7,17	14,95	22,61	18,91	10,88	8,47
	4.	5,38	7,34	11,65	20,64	25,42	19,35	18,89	20,14
	5.	2,89	6,09	10,93	19,21	26,79	43,14	19,71	15,76
	6.	5,14	6,40	7,57	11,86	12,87	15,79	14,22	9,80
	7.	0,48	5,21	5,16	28,84	29,92	22,62	16,52	7,11
	8.	0,11	5,21	16,17	22,23	25,42	20,26	7,86	5,70
	9.	0,00	0,13	4,50	16,76	18,85	11,30	4,12	2,94
	10.	0,95	0,85	8,20	20,90	22,64	15,94	4,39	4,48
	11.	10,02	11,51	18,52	23,89	12,86	14,00	10,35	10,95
	12.	3,16	3,84	11,29	16,86	14,44	10,42	6,44	3,85
	Avg	3,15	5,43	10,98	20,13	21,06	19,82	11,64	8,87
	SD	3,10	3,64	5,37	6,06	6,49	9,66	5,36	5,01

A.

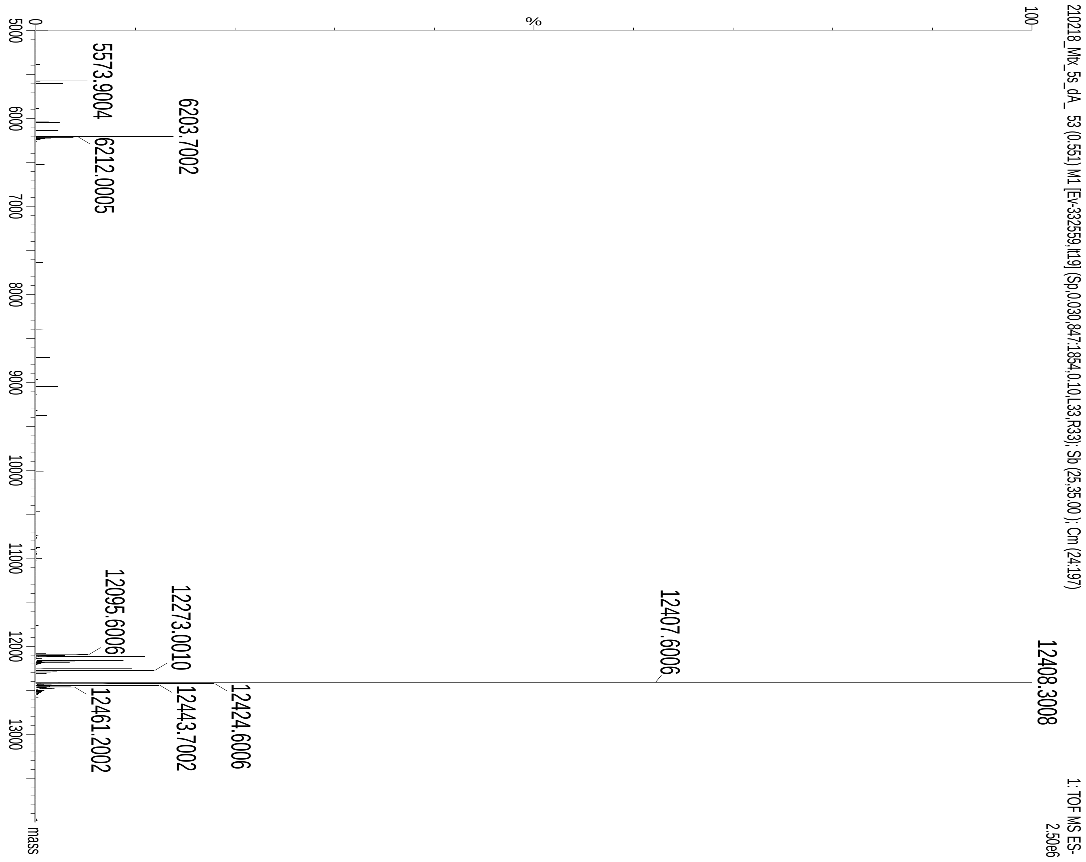
210218_Mtx_5s_dA_ 53 (0.551) M1 [Ev-332559,It19] (Sp,0.030,847:1854,0.10,L33,R33); Sb (25,35.00); Cm (24:197)
1: TOF MS ES-
2.50e6
100
%
0
12408.3008
12407.6006
12424.6006
12443.7002
12461.2002
12273.0010
12095.6006
6203.7002
6212.0005
5573.9004
5000
6000
7000
8000
9000
10000
11000
12000
13000
mass

B.

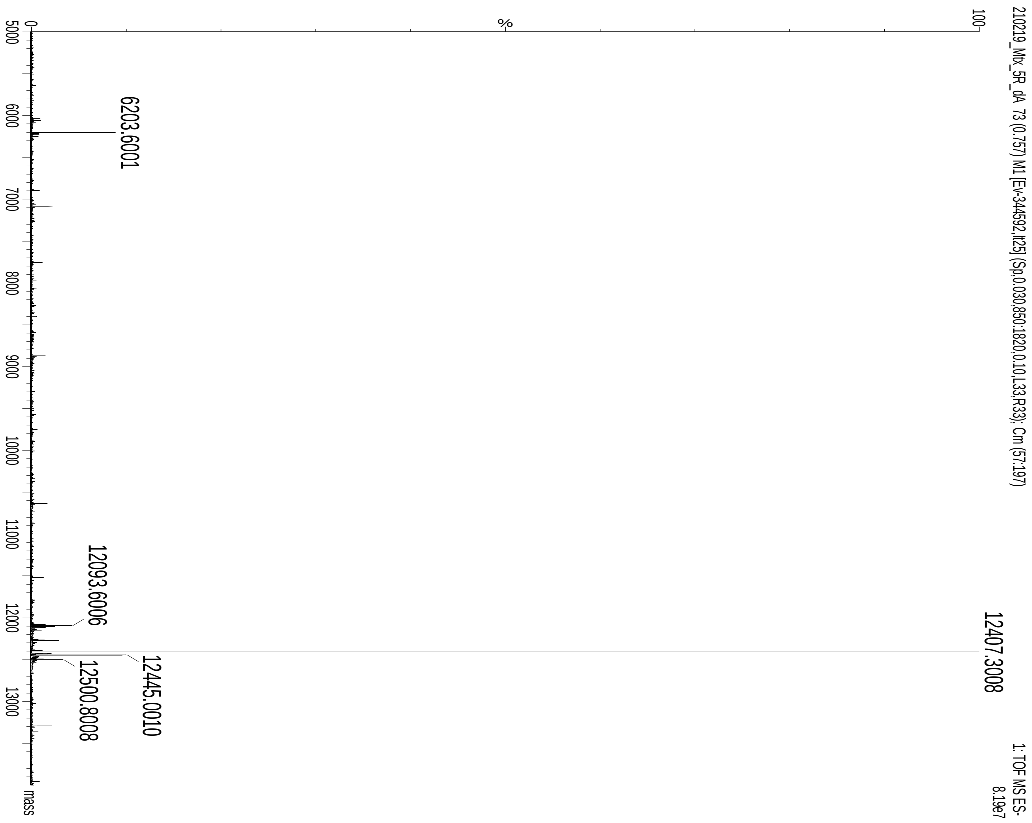
210219_Mtx_5R_dA_ 73 (0.757) M1 [Ev-344592,It25] (Sp,0.030,850:1820,0.10,L33,R33); Cm (57:197)
1: TOF MS ES-
8.19e7
100
%
0
12407.3008
12445.0010
12500.8008
12093.6006
6203.6001
5000
6000
7000
8000
9000
10000
11000
12000
13000
mass

C.

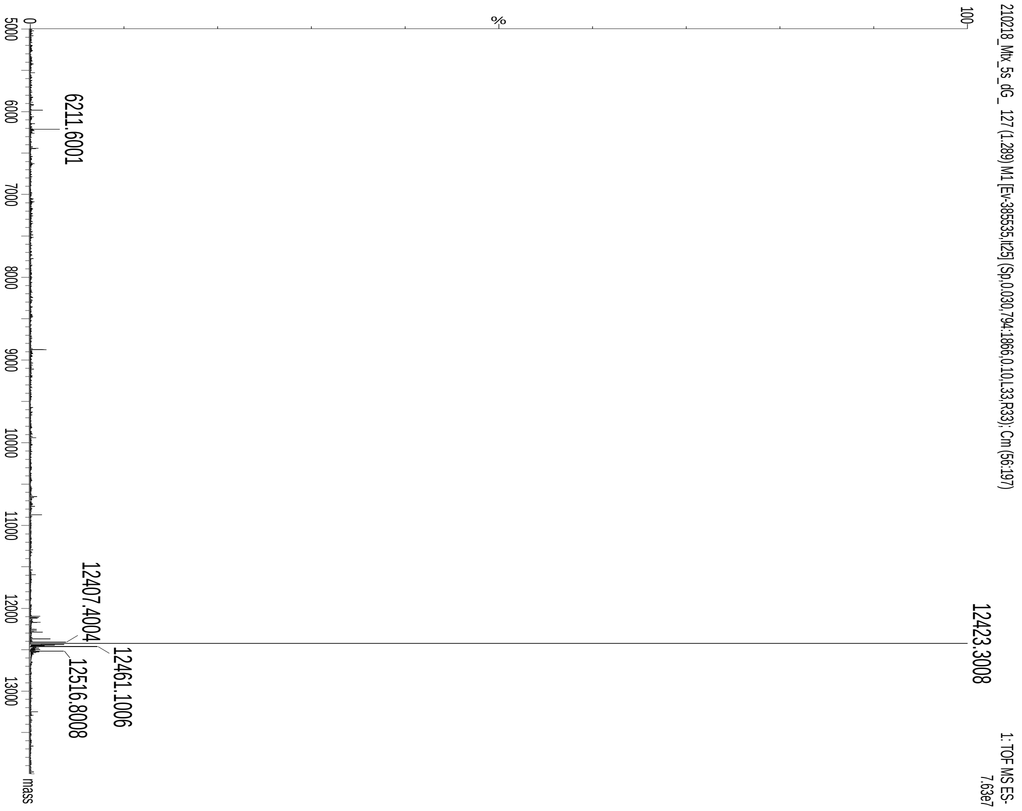
210218_Mtx_5s_dG_ 127 (1.289) M1 [Ev-365535,It25] (Sp,0.030,794:1866,0.10,L33,R33); Cm (56:197)
1: TOF MS ES-
7.63e7
100
%
0
12423.3008
12407.4004
12461.1006
12516.8008
6211.6001
5000
6000
7000
8000
9000
10000
11000
12000
13000
mass

D.

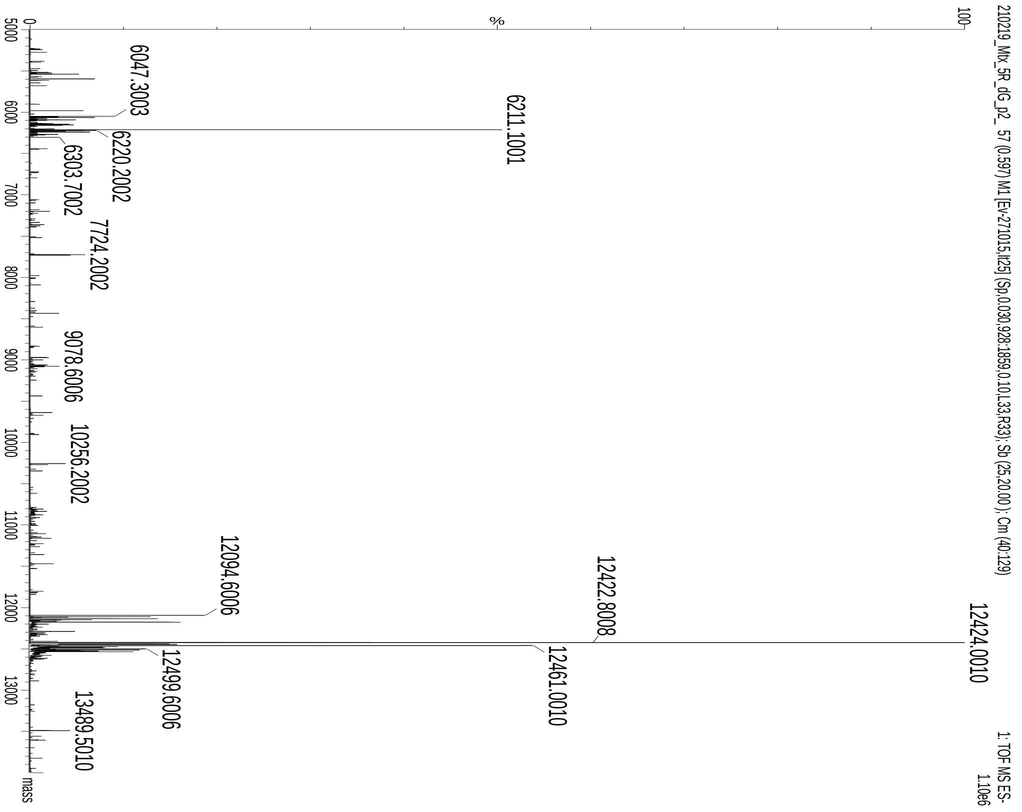
210219_Mtx_5R_dG_p2_ 57 (0.597) M1 [Ev-27101,It25] (Sp,0.030,928:1859,0.10,L33,R33); Sb (25,20.00); Cm (40:129)
1: TOF MS ES-
1.10e6
100
%
0
12424.0010
12422.8008
12461.0010
12094.6006
12499.6006
13489.5010
6211.1001
6047.3003
6220.2002
6303.7002
7724.2002
9078.6006
10256.2002
5000
6000
7000
8000
9000
10000
11000
12000
13000
mass

E.

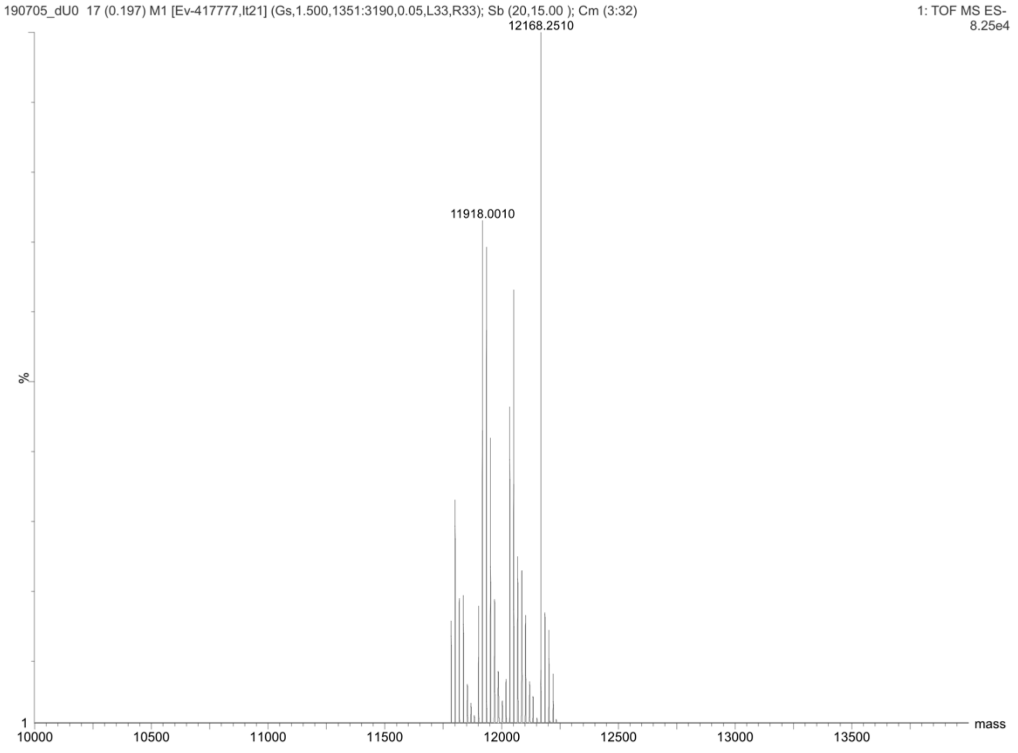

F.

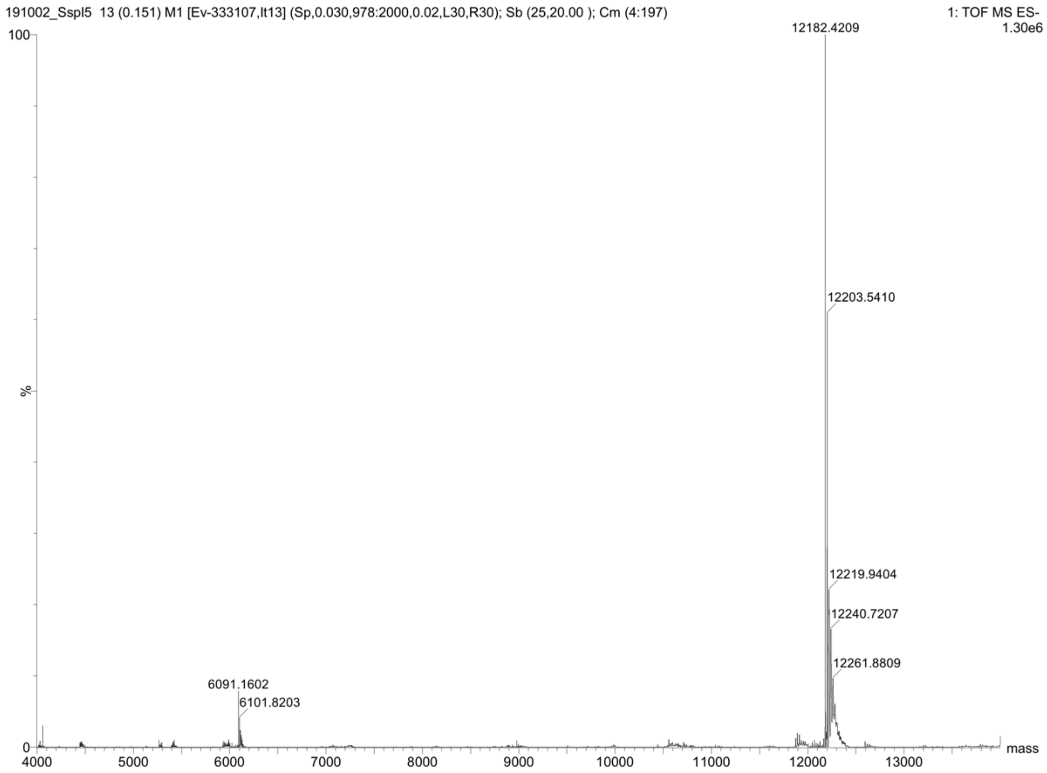

Figure S14. Mass spectra of substrate oligonucleotides containing cdPu. (A) ssDNA with (5′S)-5′,8-cyclo-2′-deoxyadenosine (Mtx-ScdA); (B) ssDNA with (5′R)-5′,8-cyclo-2′-deoxyadenosine (Mtx-RcdA); (C) ssDNA with (5′S)-5′,8-cyclo-2′-deoxyguanosine (Mtx-ScdG); (D) ssDNA with (5′R)-5′,8-cyclo-2′-deoxyguanosine (Mtx-RcdG); (E) ssDNA with 2′-deoxyuridine (Control 1 – dU strand); (F) native ssDNA (Control 1 – native strand)
